# Comparative outcomes of arteriovenous fistulas and grafts in haemodialysis: meta-analysis with subgroup analysis by fistula type and transposition status

**DOI:** 10.1093/bjsopen/zraf165

**Published:** 2026-04-01

**Authors:** Chiehlun Yang, Wenhsing Yang, Leijuan Xiao, Runzhang Zhu, Xiaofeng Li, Erqing Xiang, Hongying Wang, Jizhuang Lou, Zhanhui Gao

**Affiliations:** Department of Nephrology, Nanjing BenQ Medical Center, The Affiliated BenQ Hospital of Nanjing Medical University, Nanjing, Jiangsu Province 210019, China; Department of Nursing, New York City College of Technology, New York 11201, USA; Department of Nephrology, Nanjing BenQ Medical Center, The Affiliated BenQ Hospital of Nanjing Medical University, Nanjing, Jiangsu Province 210019, China; Department of Nephrology, Nanjing BenQ Medical Center, The Affiliated BenQ Hospital of Nanjing Medical University, Nanjing, Jiangsu Province 210019, China; Department of Nephrology, Nanjing BenQ Medical Center, The Affiliated BenQ Hospital of Nanjing Medical University, Nanjing, Jiangsu Province 210019, China; Department of Nephrology, Nanjing BenQ Medical Center, The Affiliated BenQ Hospital of Nanjing Medical University, Nanjing, Jiangsu Province 210019, China; Department of Nephrology, Nanjing BenQ Medical Center, The Affiliated BenQ Hospital of Nanjing Medical University, Nanjing, Jiangsu Province 210019, China; Department of Nephrology, Nanjing BenQ Medical Center, The Affiliated BenQ Hospital of Nanjing Medical University, Nanjing, Jiangsu Province 210019, China; Department of Nephrology, Nanjing BenQ Medical Center, The Affiliated BenQ Hospital of Nanjing Medical University, Nanjing, Jiangsu Province 210019, China

**Keywords:** arteriovenous access, failure rate, complications, vascular access, patency

## Abstract

**Background:**

Arteriovenous fistulas (AVFs) are preferred over arteriovenous grafts (AVGs) for haemodialysis, but effectiveness may vary by fistula subtype, access site, and transposition.

**Methods:**

In this systematic review, PubMed, Web of Science, Scopus, and CENTRAL were searched up to 11 January 2025 for comparative studies of AVF *versus* AVG in adults. Random-effects meta-analyses pooled odds ratios (ORs) with 95% confidence intervals, standardized to 1-year outcomes (2-year effects summarized secondarily). Prespecified subgroups were AVF site (forearm *versus* upper arm), AVG site (forearm, upper arm, lower limb), AVF type (radiocephalic, brachiocephalic, brachiobasilic, basilic, brachial–brachial), and transposition. Observational studies were appraised with the National Institute of Health tool; randomized clinical trials with RoB 2. Meta-regression evaluated age, diabetes, and hypertension; certainty was appraised with GRADE.

**Results:**

Sixty-three studies (357 333 patients: 226 078 AVF; 131 255 AVG) were included. At 1 year, AVF was associated with higher primary (OR = 1.61, 95% confidence interval 1.19 to 2.18), primary-assisted (OR = 1.69, 1.46 to 1.96), and secondary patency (OR = 1.69, 1.18 to 2.44), and lower overall complications (OR = 0.52, 0.34 to 0.78) and mortality (OR = 0.57, 0.34 to 0.98) *versus* AVG; primary failure and revision did not differ. Benefits were marked for transposed AVFs (primary-assisted OR = 2.07, 1.45 to 2.95; complications OR = 0.62, 0.43 to 0.88) and for upper-arm AVFs in primary patency (OR = 1.56, 1.05 to 2.31). Basilic AVF improved secondary patency (OR = 2.31, 1.17 to 4.57). Infection and thrombosis risks were lower for basilic and brachiobasilic AVF and upper-arm AVF. Meta-regression suggested diabetes modified effects (better secondary patency; fewer revisions), whereas age and hypertension did not. Certainty was generally low to moderate.

**Conclusion:**

When standardized at 1 year, AVFs outperform AVGs for patency, complications, and mortality, with magnitude varying by site, transposition, and fistula type. Findings support individualized access planning and the role of transposed upper-arm AVFs when feasible.

**Registration number:**

CRD420251125422 (https://www.crd.york.ac.uk/PROSPERO/home).

## Teaser

This systematic review and meta-analysis compared arteriovenous fistulas and grafts in over 260 000 haemodialysis patients, incorporating detailed subgroup analyses by arteriovenous fistula subtype, transposition status, and follow-up duration. Arteriovenous fistulas demonstrated significantly higher primary and secondary patency rates, fewer complications, and improved long-term survival, underscoring the need for individualized vascular access planning based on anatomical and procedural nuances.

## Introduction

Vascular access remains the lifeline for patients undergoing haemodialysis, directly influencing treatment efficacy, patient survival, and healthcare costs^1^. Among available options, arteriovenous fistulas (AVFs) are the preferred access due to their superior long-term patency and lower complication rates compared with arteriovenous grafts (AVGs)^1^. Despite this, AVFs are associated with prolonged maturation time and a high primary failure rate, which has led to increasing consideration of AVGs as an alternative, particularly in patients with poor vascular anatomy^2^. The decision between AVF and AVG placement is further complicated by the significant variability in reported patency rates, success rates, and complication profiles across different AVF subtypes, leading to inconsistent clinical recommendations.

Whereas previous meta-analyses^3–5^ have confirmed the overall advantages of AVFs over AVGs, most have treated AVFs as a single entity, without differentiating between subtypes such as brachiocephalic AVFs (BCAVFs), brachiobasilic AVFs (BBAVFs), radiocephalic AVFs (RCAVFs), basilic vein AVFs (BaAVFs), brachial–brachial AVFs (BrAVFs), and ulnar artery AVFs (UA-AVFs). Additionally, transposed (tAVF) and non-transposed (ntAVF) AVFs exhibit distinct haemodynamic and surgical characteristics, yet little is known about how these variations impact long-term outcomes. The lack of detailed comparisons across AVF subtypes and transposition status represents a major gap in the current literature, leaving clinicians with limited evidence to guide individualized vascular access decisions.

Another key challenge in vascular access research is the heterogeneity in follow-up duration across studies, making direct comparisons of AVF and AVG outcomes difficult^6^. Previous systematic reviews have attempted to address this by performing pooled analyses^4,7^, yet they often fail to account for differences in study design, patient populations, and reporting standards. Given these inconsistencies, a comprehensive, high-quality meta-analysis that stratifies outcomes based on AVF subtype, transposition status, and follow-up duration is critically needed to refine vascular access selection and optimize patient outcomes.

This systematic review and meta-analysis aims to address these gaps by providing the most detailed comparative analysis to date of AVFs and AVGs in patients undergoing haemodialysis. Unlike previous studies, subgroup analyses were conducted rather than a pooled meta-analysis to account for variations in follow-up duration and AVF subtype, ensuring a more clinically meaningful interpretation of the data. By systematically evaluating patency rates, complications, mortality, and functional outcomes across different AVF configurations, this study provides granular evidence to guide vascular access selection in patients undergoing haemodialysis.

## Methods

### Protocol and reporting

This systematic review and meta-analysis followed PRISMA 2020 guidance and the study protocol was registered on PROSPERO^8^.

### Eligibility criteria

Comparative studies of adult patients undergoing haemodialysis reporting outcomes for AVF *versus* AVG were included. Randomized clinical trials (RCTs) and observational cohorts (prospective or retrospective) were eligible. Single-arm studies, studies with < 10 participants, non-original reports (reviews, editorials, conference abstracts), studies reporting only baseline AVF failure cohorts, subgroup/*post hoc* reanalyses without a full comparator, and reports without group-stratified data were excluded.

### Outcomes

Primary outcomes were primary patency, primary-assisted patency, secondary patency, and functional patency (primary and secondary). Secondary outcomes were primary failure, ‘success’ (as reported by the original study), revision surgery, complications (overall and specific, including infection, thrombosis, stenosis, seroma, pseudoaneurysm, steal), and mortality. Owing to inconsistent and time-dependent reporting, all comparative syntheses were standardized to 1-year outcomes *a priori*; 2-year effects were summarized secondarily. Overall survival was not analysed.

### Information sources and search

PubMed, Web of Science, Scopus, and CENTRAL (Cochrane Library) were searched from inception to 11 January 2025, using medical subject headings (MeSH) and free-text terms for dialysis vascular access (‘fistula/AVF’, ‘graft/AVG’) combined with ‘dialysis/haemodialysis’; search strings for each database are in *Table S1*. Filters were applied to remove reviews and meta-analyses. Manual methods included backward/forward citation chasing, PubMed ‘Similar Articles’, and a targeted Google Scholar check. No language or date restrictions were imposed.

### Study selection and de-duplication

**Fig. 1:**
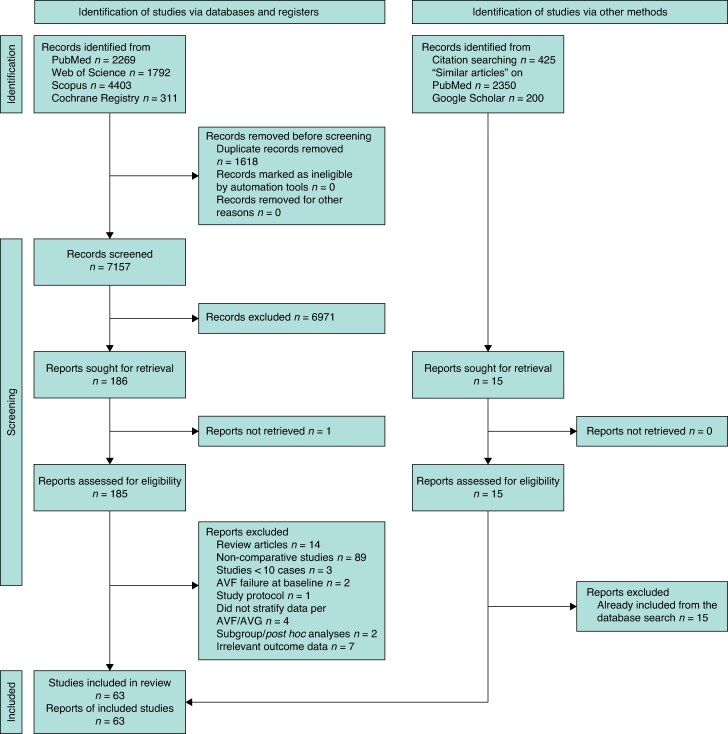
PRISMA flow diagram showing the results of the literature search and screening processes AVF, arteriovenous fistula; AVG, arteriovenous graft.

Records were de-duplicated using a citation manager (EndNote, Clarivate) and manually verified. Two reviewers independently screened titles/abstracts, then full texts against prespecified criteria; disagreements were resolved by consensus or a third reviewer. Reasons for exclusion at full text are documented in *Fig. 1*.

### Data extraction

Using a piloted template, two reviewers extracted study characteristics; population demographics; comparator details; AVF subtype (RCAVF, BCAVF, BBAVF, BaAVF, BrAVF), transposition status (transposed *versus* non-transposed), and access site (AVF: forearm *versus* upper-arm; AVG: forearm *versus* upper-arm *versus* lower-limb); follow-up duration; and all outcomes at 1 year (and 2 years where available). When outcomes were available only graphically, 1-year estimates were digitized with PlotDigitizer. Where outcome definitions varied (for example ‘primary failure’, ‘success’, ‘patency’), results were extracted as reported and study-level definitions were catalogued.

### Risk of bias assessment

Observational studies were appraised with the National Institute of Health tool^9^; RCTs with RoB 2^10^. Two reviewers assessed risk of bias independently with consensus resolution.

### Data synthesis and statistical analysis

Given clinical and methodological heterogeneity, odds ratios (ORs) were pooled with 95% confidence intervals (c.i.) using random-effects models, and heterogeneity was quantified with I^2^ (and τ^2^). Small-study effects were evaluated with Egger's test and funnel plots where ten or more studies were available. To minimize time-dependent bias, all primary syntheses were standardized to 1-year outcomes, with 2-year effects summarized secondarily. Prespecified subgroup analyses at 1 year compared AVF site (forearm *versus* upper arm), AVG site (forearm, upper arm, lower limb), AVF type (RCAVF, BCAVF, BBAVF, BaAVF, BrAVF), and transposition status (transposed *versus* non-transposed); subgroups represented by a single study were reported in figures but not in tables or emphasized narratively. Random-effects meta-regression was performed restricted to 1-year effects with mean age, diabetes mellitus prevalence, and hypertension prevalence as covariables; additional factors (first access, previous catheter, previous AVF/AVG) were considered but not modelled due to sparse reporting (fewer than 5 studies). Certainty of evidence was appraised using GRADE. All analyses used Stata^®^ v18 (StataCorp, LLC, College Station, TX, USA), and two-sided *P* < 0.05 denoted statistical significance.

## Results

### Literature search results

Database searches identified 8775 records (*Fig. 1*). Manual methods yielded 2975 additional records (citation chasing *n* = 425; PubMed ‘Similar Articles’ *n* = 2350; Google Scholar *n* = 200). After removing 1618 duplicates, 7157 records remained for screening. Some 6971 were excluded at title/abstract stage and 186 full texts were assessed (1 not retrievable, leaving 185). Of these, 122 were excluded. Fifteen additional records identified manually were duplicates of database hits. Sixty-three studies met inclusion criteria for the systematic review and meta-analysis^11–73^.

### Baseline characteristics of included studies

*Table S2* summarizes study features. Most studies originated from the United States (36 of 63; 57.1%), followed by the UK and Canada (each 4 of 63; 6.3%), and The Netherlands and Korea (each 3 of 63, 4.8%). Study designs were predominantly retrospective cohorts (49 of 63; 77.8%), with prospective cohorts (8 of 63; 12.7%) and RCTs (6 of 63; 9.5%). Across 357 333 patients, 226 078 received AVF and 131 255 received AVG. Mean age was reported by 56 studies (pooled mean 63.11 years). Previous AVF/AVG use (12 studies) and ‘first access’ status (13 studies) accounted for 46.8% and 43.1% of participants, respectively; time on dialysis was reported in four studies. Previous catheter use (11 studies) involved 54.7% of patients. Comorbidities were common: hypertension in 38 studies (pooled 66.4%) and diabetes in 53 studies (pooled 47.9%). Other conditions (coronary artery disease, peripheral vascular disease, stroke, heart failure, cancer, smoking) are detailed in *Table S3*.

### Methodological quality

**Fig. 2:**
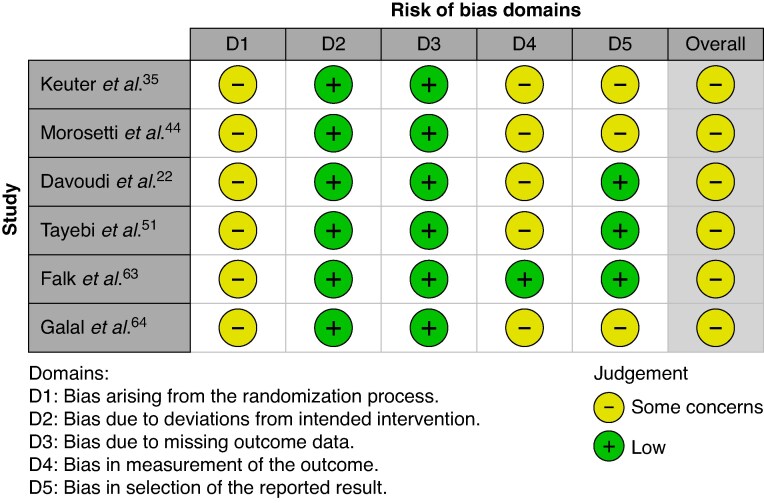
The risk of bias of included randomized clinical trials using the Cochrane's RoB-2 tool

Among the 57 observational studies, 44 (77.2%) were rated fair and 13 (22.8%) good (*Table S4*). All six RCTs were judged to have some concerns, mainly due to incomplete reporting of randomization and outcome measurement (*Fig. 2*).

### Outcomes

A summary of all pooled meta-analytic estimates with corresponding GRADE certainty ratings is provided in *Table 1* along with referenced *Figs 3–6* and *Figs S1–S30*.

**Fig. 3:**
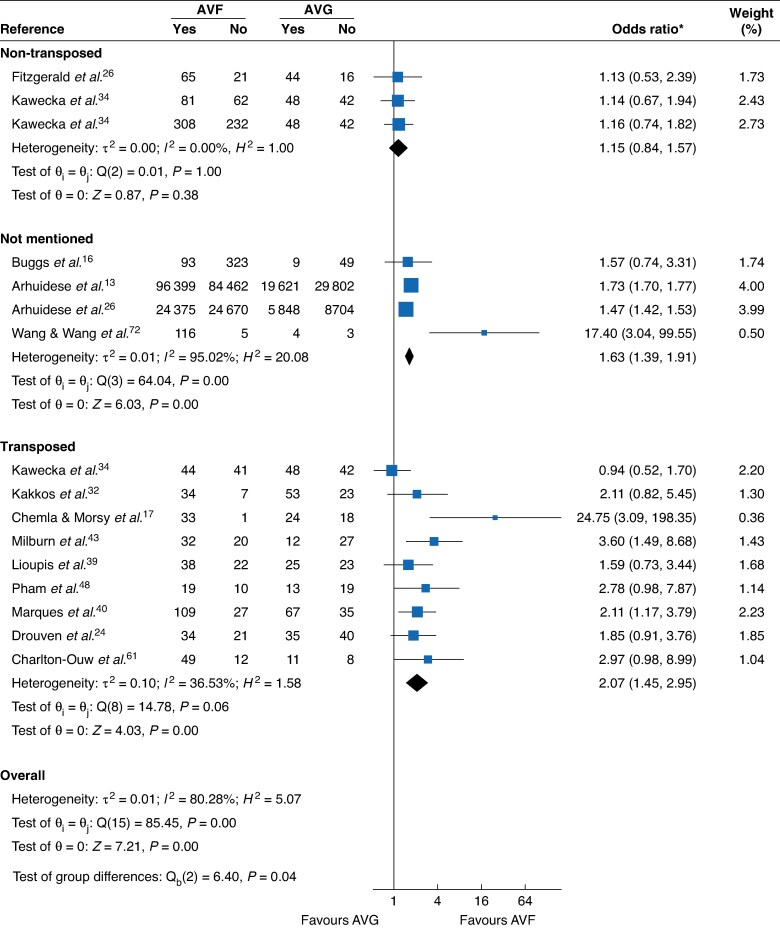
Primary-assisted patency by AVF transposition status (1 year) *Values in parentheses are 95% confidence intervals. AVF, arteriovenous fistula; AVG, arteriovenous graft; c.i., confidence interval.

**Fig. 4:**
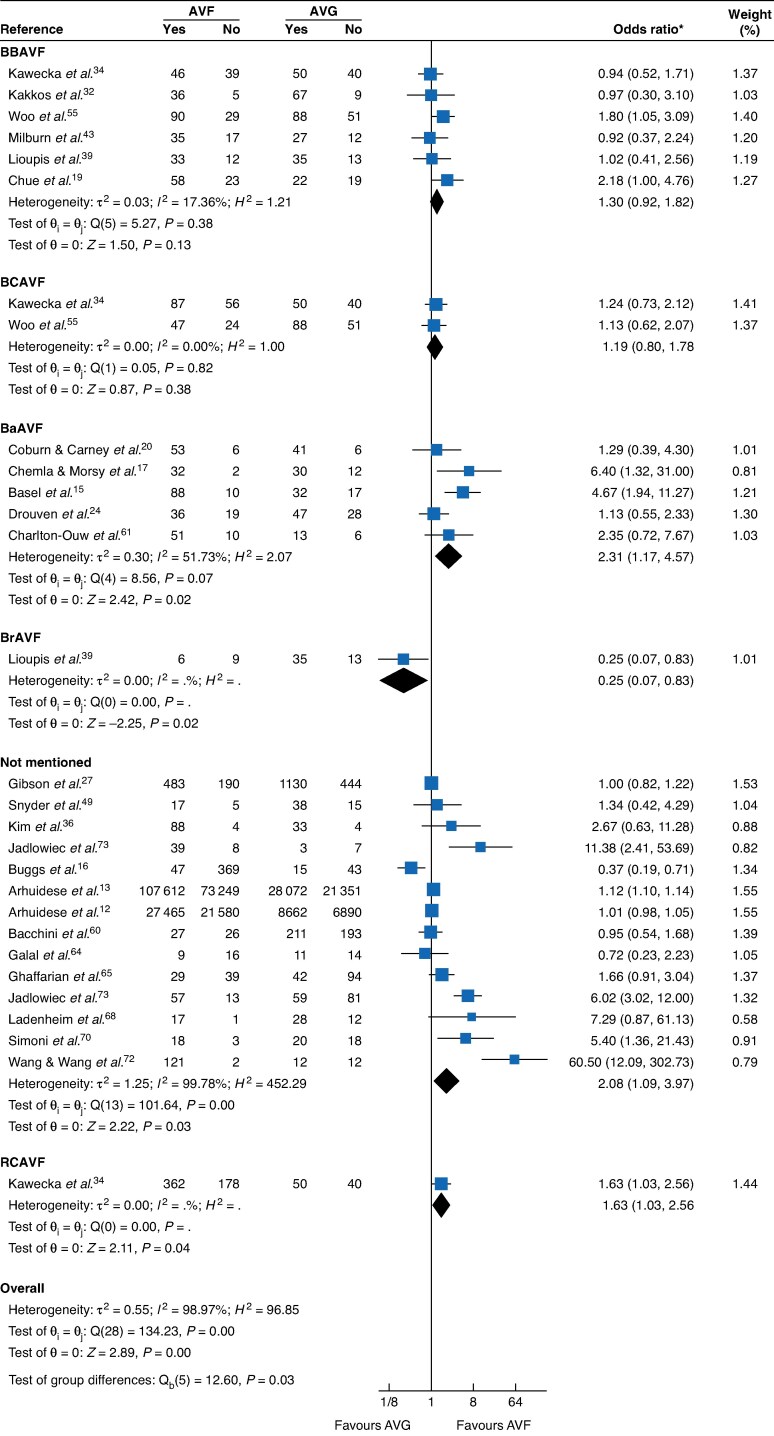
Secondary patency by AVF type (1 year) *Values in parentheses are 95% confidence intervals. AVF, arteriovenous fistula; AVG, arteriovenous graft; c.i., confidence interval.

**Fig. 5:**
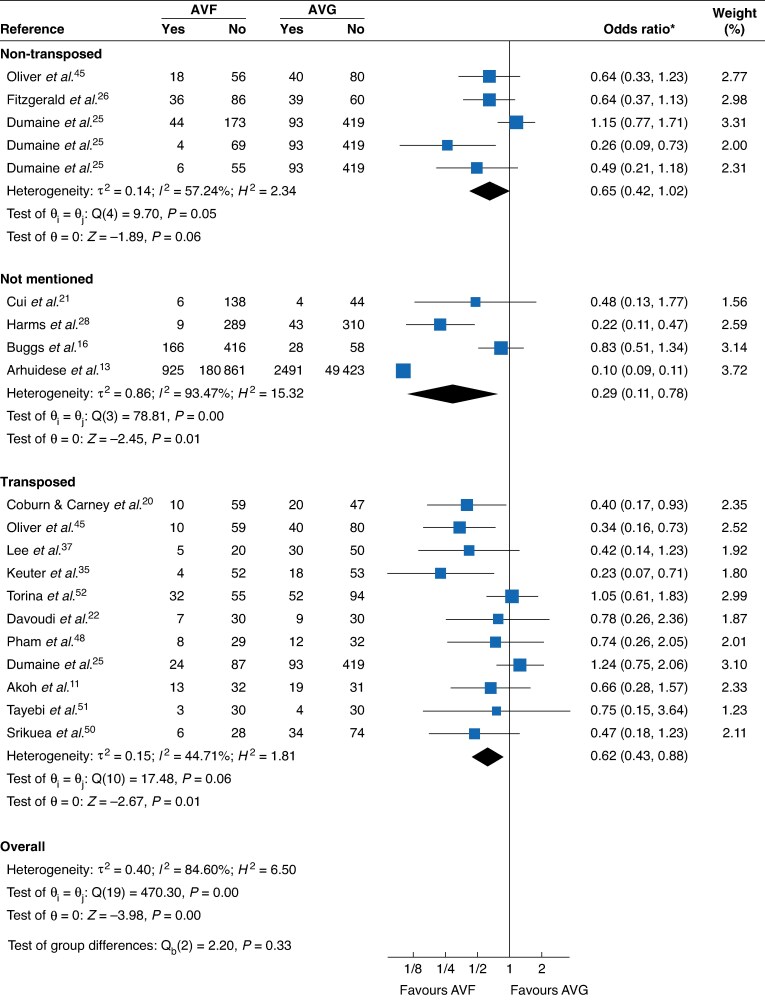
Complications by AVF transposition status (1 year) *Values in parentheses are 95% confidence intervals. AVF, arteriovenous fistula; AVG, arteriovenous graft; c.i., confidence interval.

**Fig. 6:**
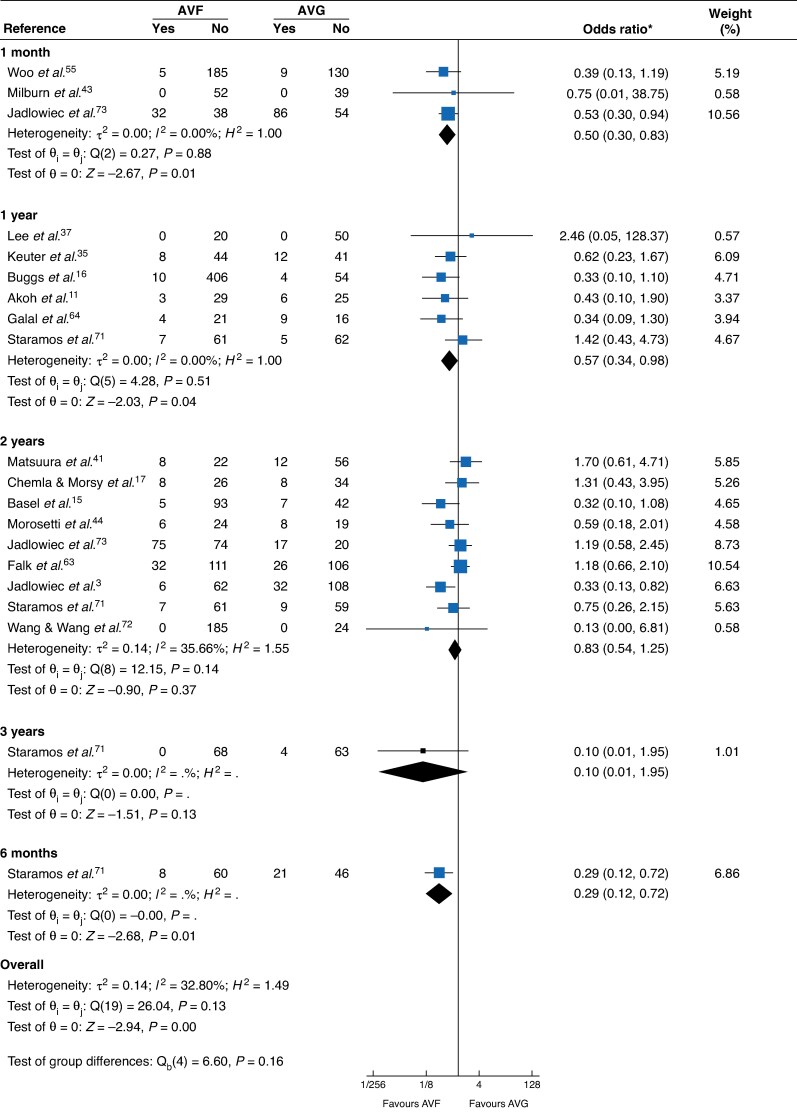
Mortality by time *Values in parentheses are 95% confidence intervals. AVF, arteriovenous fistula; AVG, arteriovenous graft; c.i., confidence interval.

**Table 1:** Summary of the meta-analytic estimates of the examined outcomes (and subgroups) with the level of evidence certainty

	Studies	OR	I^2^	GRADE	Figure
**Primary patency**					
Time					
1 year	32	1.61 (1.19, 2.18)	98.81%	Low	*Fig. S1*
2 years	20	2.38 (1.62, 3.49)	99.05%	Low	
AVF type					
Transposed	18	1.63 (1.04, 2.54)	85.57%	Low	*Fig. S2*
Non-transposed	11	1.98 (1.34, 2.90)	85.78%	Low	
BBAVF *versus* AVG	9	1.31 (0.58, 2.95)	88.51%	Very low	*Fig. S3*
BCAVF *versus* AVG	4	2.05 (0.82, 5.13)	91.07%	Very low	
BaAVF *versus* AVG	5	1.41 (0.83, 2.40)	45.58%	Low	
BrAVF *versus* AVG	2	2.16 (0.40, 11.59)	74.35%	Very low	
RCAVF *versus* AVG	3	1.62 (0.86, 3.06)	83.64%	Very Low	
AVF site					
Forearm AVF *versus* AVG	3	1.62 (0.86, 3.06)	83.64%	Very Low	*Fig. S4*
Upper-arm AVF *versus* AVG	20	1.56 (1.05, 2.31)	82.51%	Low	
AVF *versus* FA AVG	3	1.03 (0.68, 1.54)	0%	Moderate	*Fig. S5*
AVF *versus* UA AVG	10	1.16 (0.59, 2.28)	88.32%	Low	
AVF *versus* FA/UA AVG	9	2.17 (1.18, 3.97)	76.89%	Low	
**Primary-assisted patency**					
Time					
1 year	14	1.69 (1.46, 1.96)	85.73%	Low	*Fig. S6*
2 years	9	2.21 (2.09, 2.34)	46.81%	Low	
AVF type					
Transposed	9	2.07 (1.45, 2.95)	36.53%	Moderate	*Fig. 3*
Non-transposed	3	1.15 (0.84, 1.57)	0%	Moderate	
BBAVF *versus* AVG	5	1.95 (1.21, 3.14)	50.12%	Low	*Fig. S7*
BaAVF *versus* AVG	3	3.76 (1.09, 12.93)	69.33%	Very Low	
BrAVF *versus* AVG	2	1.16 (0.20, 6.76)	79.41%	Very Low	
AVF site					
Upper-arm AVF *versus* AVG	12	1.69 (1.24, 2.30)	42.40%	Low	*Fig. S8*
AVF *versus* FA AVG	2	1.47 (0.87, 2.46)	0%	Moderate	*Fig. S9*
AVF *versus* UA AVG	5	2.56 (1.29, 5.07)	71.47%	Very Low	
AVF *versus* FA/UA AVG	3	3.47 (1.01, 11.92)	72.41%	Very Low	
**Secondary patency**					
Time					
1 year	24	1.69 (1.18, 2.44)	99.22%	Low	*Fig. S10*
2 years	16	2.16 (1.27, 3.66)	99.72%	Low	
AVF type					
Transposed	12	1.39 (0.98, 1.97)	58.39%	Low	*Fig. S11*
Non-transposed	5	1.22 (0.98, 1.51)	14.37%	Moderate	
BBAVF *versus* AVG	6	1.30 (0.92, 1.82)	17.36%	Moderate	*Fig. 4*
BCAVF *versus* AVG	2	1.19 (0.80, 1.78)	0%	Moderate	
BaAVF *versus* AVG	5	2.31 (1.17, 4.57)	51.73%	Low	
AVF site					
Upper-arm AVF *versus* AVG	13	1.32 (0.93, 1.88)	56.87%	Low	*Fig. S12*
AVF *versus* FA AVG	2	1.54 (0.81, 2.94)	31.42%	Moderate	*Fig. S13*
AVF *versus* UA AVG	8	1.64 (1.10, 2.42)	52.47%	Low	
AVF *versus* FA/UA AVG	6	3 (0.91, 9.82)	86.67%	Very Low	
**Primary failure**					
Time					
1 year	14	1.32 (0.85, 2.03)	81.20%	Low	*Fig. S14*
2 years	2	1.36 (0.87, 2.11)	0%	Moderate	
AVF type					
Transposed	9	0.92 (0.61, 1.39)	30.51%	Moderate	*Fig. S15*
Non-transposed	2	3.33 (2.33, 4.75)	0%	Moderate	
BBAVF *versus* AVG	4	0.82 (0.38, 1.79)	65.92%	Low	*Fig. S16*
BCAVF *versus* AVG	2	2.77 (1.62, 4.74)	56.56%	Low	
BaAVF *versus* AVG	3	0.94 (0.42, 2.08)	0%	Moderate	
AVF site					
Upper-arm AVF *versus* AVG	10	1.16 (0.69, 1.93)	70.69%	Low	*Fig. S17*
AVF *versus* FA AVG	2	1.45 (0.62, 3.37)	0%	Moderate	*Fig. S18*
AVF *versus* UA AVG	8	1.33 (0.73, 2.45)	79.22%	Low	
AVF *versus* FA/UA AVG	3	2.06 (0.97, 4.38)	68.53%	Very Low	
**Success**					
Time					
1 year	13	1.15 (0.67, 1.98)	95.97%	Low	*Fig. S19*
2 years	3	3.20 (1.80, 5.70)	26.15%	Low	
AVF type					
Transposed	11	1.48 (0.88, 2.49)	71.48%	Low	*Fig. S20*
Non-transposed	5	0.38 (0.13, 1.07)	94.11%	Low	
BBAVF *versus* AVG	5	0.97 (0.68, 1.38)	0%	Moderate	*Fig. S21*
BCAVF *versus* AVG	3	0.60 (0.23, 1.60)	91.66%	Low	
BaAVF *versus* AVG	4	2.95 (1.15, 7.57)	72.70%)	Very low	
AVF site					
Upper-arm AVF *versus* AVG	13	1.23 (0.73, 2.08)	84.10%	Low	*Fig. S22*
AVF *versus* UA AVG	10	1.17 (0.56, 2.45)	87.73%	Low	*Fig. S23*
AVF *versus* FA/UA AVG	3	0.35 (0.22, 0.56)	23.77%	Moderate	
**Complications**					
Time					
1 year	14	0.52 (0.34, 0.78)	86.81%	Low	*Fig. S24*
2 years	10	0.63 (0.41, 0.97)	75.75%	Low	
AVF type					
Transposed	11	0.62 (0.43, 0.88)	44.71%	Moderate	*Fig. 5*
Non-transposed	5	0.65 (0.42, 1.02)	57.24%	Low	
BBAVF *versus* AVG	5	0.58 (0.30, 1.12)	56.68%	Low	*Fig. S25*
BaAVF *versus* AVG	4	0.70 (0.46, 1.06)	6.26%	Low	
BrAVF *versus* AVG	2	1.06 (0.54, 2.07)	0%	Low	
AVF site					
Upper-arm AVF *versus* AVG	12	0.73 (0.55, 0.98)	41.28%	Moderate	*Fig. S26*
AVF *versus* FA AVG	2	0.43 (0.16, 1.17)	61.05%	Low	*Fig. S27*
AVF *versus* UA AVG	6	0.53 (0.35, 0.80)	0%	Moderate	
AVF *versus* FA/UA AVG	3	0.89 (0.56, 1.41)	0%	Moderate	
**Primary functional**					
Time					
1 year	3	1.90 (0.78, 4.65)	84.02%	Very Low	*Fig. S28*
2 years	3	1.37 (0.85, 2.20)	61.66%	Low	
**Secondary functional**					
Time					
1 year	5	0.84 (0.59, 1.19)	0%	Moderate	*Fig. S29*
2 years	2	1.54 (1.03, 2.30)	0%	Moderate	
**Mortality**					
Time					
1 year	6	0.57 (0.34, 0.98)	0%	Moderate	*Fig. 6*
2 years	9	0.83 (0.54, 1.25)	35.66%	Moderate	
**Revision Surgery**					
Time					
1 year	6	0.54 (0.26, 1.12)	76.64%	Low	*Fig. S30*

Values in parentheses are 95% confidence intervals. OR, odds ratio; c.i., confidence interval; I^2^, measure of statistical heterogeneity; AVF, arteriovenous fistula; BBAVF, brachiobasilic AVF; AVG, arteriovenous graft; BCAVF, brachiocephalic AVF; BaAVF, basilic vein AVF; BrAVF, brachial–brachial AVF; RCAVF, radiocephalic AVF; FA, forearm; UA, upper arm.

### Primary patency

At 1 year, AVF was associated with higher primary patency than AVG (32 studies; OR = 1.61, 95% c.i. 1.19 to 2.18; I^2^ = 98.8%; low certainty). The effect persisted at 2 years (20 studies; OR = 2.38, 1.62 to 3.49; I^2^ = 99.1%; low certainty). By transposition, both transposed (18 studies; OR = 1.63, 1.04 to 2.58; I^2^ = 85.6%; low certainty) and non-transposed AVFs (11 studies; OR = 1.98, 1.34 to 2.90; I^2^ = 85.8%; low certainty) favoured AVF over AVG. By site, upper-arm AVF outperformed AVG (20 studies; OR = 1.56, 1.05 to 2.31; I^2^ = 82.5%; low certainty), whereas the pooled forearm AVF estimate was imprecise (3 studies; OR = 1.62, 0.86 to 3.06; I^2^ = 83.6%; very low certainty). When stratified by AVG site, effects were neutral *versus* forearm OR = upper-arm grafts individually but favoured AVF *versus* mixed forearm/upper-arm graft comparators (9 studies; OR = 2.17, 1.18 to 3.97; I^2^ = 76.89%; low certainty). Type-specific analyses (BBAVF, BCAVF, BaAVF, BrAVF, RCAVF) were inconclusive at 1 year (very low–low certainty).

There was no evidence that age (*P* = 0.65), diabetes (*P* = 0.44), or hypertension (*P* = 0.26) modified the effect of AVF *versus* AVG on primary patency (*Table 2*).

**Table 2:** Multivariable meta-regression findings of the determinants of reported outcomes (AVF *versus* AVG in haemodialysis)

Risk factor	Coefficient	SE	*Z*	P*	Low c.i.	High c.i.
**Success**						
Age (years) (per year increase), mean	0.044	0.218	0.200	0.841	−0.383	0.470
DM (per % increase)	0.019	0.136	0.140	0.891	−0.248	0.285
Hypertension (per % increase)	−0.012	0.036	−0.330	0.741	−0.082	0.058
**Primary failure**						
Age (years) (per year increase), mean	−0.052	0.073	−0.720	0.474	−0.194	0.090
Hypertension (per % increase)	−0.029	0.031	−0.940	0.349	−0.089	0.032
DM (per % increase)	0.031	0.055	0.570	0.570	−0.077	0.140
**Revision surgery**						
Age (years) (per year increase), mean	−0.058	0.043	−1.360	0.173	−0.141	0.025
DM (per % increase)	−0.087	0.025	−3.560	< 0.001	−0.135	−0.039
**Primary patency**						
Age (years) (per year increase), mean	0.004	0.008	0.450	0.651	−0.013	0.020
Hypertension (per % increase)	−0.011	0.010	−1.140	0.256	−0.031	0.008
DM (per % increase)	0.013	0.017	0.780	0.436	−0.020	0.045
**Primary-assisted patency**						
Age (years) (per year increase), mean	−0.002	0.004	−0.450	0.649	−0.009	0.005
Hypertension (per % increase)	0.009	0.006	1.520	0.128	−0.003	0.021
DM (per % increase)	0.010	0.007	1.400	0.161	−0.004	0.024
**Secondary patency**						
Age (years) (per year increase), mean	−0.011	0.008	−1.290	0.197	−0.027	0.006
Hypertension (per % increase)	−0.001	0.013	−0.080	0.939	−0.026	0.024
DM (per % increase)	0.039	0.018	2.110	0.035	0.003	0.075
**Complications**						
Age (years) (per year increase), mean	0.127	0.325	0.390	0.695	−0.509	0.764
Hypertension (per % increase)	0.002	0.032	0.070	0.941	−0.060	0.064
DM (per % increase)	0.117	0.143	0.820	0.411	−0.163	0.397
Previous catheter use (per % increase)	−0.013	0.060	−0.210	0.834	−0.130	0.105

Although other factors like first access, previous catheter use, and history of previous AVF/AVG use were considered, they were not added to this multivariable model either because relevant data were missing or because of the small sample (fewer than 5 studies per covariate). AVF, arteriovenous fistula; AVG, arteriovenous graft; SE, standard error; c.i., confidence interval; DM, diabetes mellitus; Z, Z-statistic. *P-values for individual predictors were obtained using Wald-type tests of the corresponding regression coefficients.

### Primary-assisted patency

At 1 year, AVF showed higher primary-assisted patency (14 studies; OR = 1.69, 95% c.i. 1.46 to 1.96; I^2^ = 85.7%; low certainty) with a larger effect at 2 years (9 studies; OR = 2.21, 2.09 to 2.34; I^2^ = 46.8%; low certainty). Transposed AVF demonstrated a clear advantage (9 studies; OR = 2.07, 1.45 to 2.95; I^2^ = 36.5%; moderate certainty), whereas non-transposed estimates were null (3 studies; OR = 1.15, 0.84 to 1.57; I^2^ = 0%; moderate certainty). By type, BBAVF (5 studies; OR = 1.95, 1.23 to 3.11; I^2^ = 50.1%; low certainty) and BaAVF (3 studies; OR = 3.76, 1.09 to 12.93; I^2^ = 69.3%; very low certainty) favoured AVF. Upper-arm AVF *versus* AVG also favoured AVF (12 studies; OR = 1.69, 1.24 to 2.30; I^2^ = 42.4%; low certainty). Compared with upper-arm grafts, AVF retained benefit (5 studies; OR = 2.56, 1.29 to 5.07; I^2^ = 71.5%; very low certainty).

No significant modification was observed by age (*P* = 0.65), diabetes (*P* = 0.16), or hypertension (*P* = 0.13) (*Table 2*).

### Secondary patency

AVF had higher 1-year secondary patency (24 studies; OR = 1.69, 95% c.i. 1.18 to 2.44; I^2^ = 99.2%; low certainty) and 2-year secondary patency (16 studies; OR = 2.16, 1.27 to 3.66; I^2^ = 99.7%; low certainty). BaAVF showed advantage over AVG (5 studies; OR = 2.31, 1.17 to 4.57; I^2^ = 51.7%; low certainty), whereas other types and upper-arm AVF site analyses were imprecise. Against upper-arm grafts, AVF favoured higher secondary patency (8 studies; OR = 1.64, 1.10 to 2.42; I^2^ = 52.5%; low certainty).

Diabetes mellitus significantly modified the association between AVF and AVG, with a higher prevalence of diabetes correlating with improved secondary patency in AVFs (coefficient 0.039, *P* = 0.035). Age (*P* = 0.20) and hypertension (*P* = 0.94) were not significant modifiers (*Table 2*).

### Primary failure

At 1 year, pooled differences in primary failure were not significant (14 studies; OR = 1.32, 95% c.i. 0.85 to 2.03; I^2^ = 81.2%; low), and remained non-significant at 2 years (2 studies; OR = 1.36, 0.87 to 2.11; I^2^ = 0%; moderate certainty). By transposition, non-transposed AVFs had a higher failure rate than AVG (2 studies; OR = 3.33, 2.33 to 4.75; I^2^ = 0%; moderate certainty), whereas transposed AVFs did not (9 studies; OR = 0.92, 0.61 to 1.39; I^2^ = 30.5%; moderate certainty). Type-specific signals were inconsistent; BCAVF showed increased failure *versus* AVG (2 studies; OR = 2.77, 1.62 to 4.74; I^2^ = 56.6%; low certainty).

The effect of AVF *versus* AVG on primary failure was not significantly modified by age (*P* = 0.47), diabetes (*P* = 0.57), OR = hypertension (*P* = 0.35) (*Table 2*).

### ‘Success’ (as reported by studies)

At 1 year, pooled success did not differ (13 studies; OR = 1.15, 95% c.i. 0.67 to 1.98; I^2^ = 96.0%; low certainty) but favoured AVF at 2 years (3 studies; OR = 3.20, 1.80 to 5.70; I^2^ = 26.2%; low certainty). By type, BaAVF (4 studies; OR = 2.95, 1.15 to 7.57; I^2^ = 72.7%; very low) suggested higher success *versus* AVG, whereas other types were neutral.

Meta-regression did not identify any significant modifiers of success. Neither age (*P* = 0.84), diabetes (*P* = 0.89), nor hypertension (*P* = 0.74) significantly influenced the effect of AVF *versus* AVG (*Table 2*).

### Complications (overall)

AVF had lower overall complications at 1 year (14 studies; OR = 0.52, 95% c.i. 0.34 to 0.78; I^2^ = 86.8%; low certainty) and 2 years (10 studies; OR = 0.63, 0.41 to 0.97; I^2^ = 75.8%; low certainty). Benefit was evident for transposed AVF (11 studies; OR = 0.62, 0.43 to 0.88; I^2^ = 44.7%; moderate certainty) and for upper-arm AVF *versus* AVG (12 studies; OR = 0.73, 0.55 to 0.98; I^2^ = 41.3%; moderate certainty). Against upper-arm grafts, AVF reduced complications (6 studies; OR = 0.53, 0.35 to 0.80; I^2^ = 0%; moderate certainty). Type-specific estimates (BBAVF, BaAVF, BrAVF) were largely imprecise at 1 year.

None of the examined factors significantly modified the difference in complication rates between AVF and AVG (all *P* > 0.39) (*Table 2*).

### Complications (type-specific)

No differences were observed between AVF and AVG in terms of aneurysm/pseudoaneurysm, bleeding, limb oedema, seroma, steal syndrome, and vascular hypertension (*Table S5*). The risk of haematoma was significantly higher in BrAVF compared with AVG (2 studies, OR = 13.93; 95% c.i. 1.76 to 110.36; I^2^ = 0%, low certainty). Similarly, the risk of stenosis was higher in BaAVF compared with AVG (2 studies; OR = 2.16; 1.04 to 4.49, I^2^ = 0%, low certainty).

The risk of infection was lower in BaAVF (5 studies; OR = 0.19; 0.08 to 0.45; I^2^ = 0%, moderate certainty), BBAVF (8 studies; OR = 0.18; 0.08 to 0.43, I^2^ = 0%, moderate certainty), and UA-AVF (2 studies; OR = 0.35; 0.17 to 0.74, I^2^ = 0%, moderate certainty) when compared with AVG, respectively. The same was observed with thrombosis risk in BaAVF (4 studies; OR = 0.32; 0.15 to 0.67, I^2^ = 55.92%, low certainty), BBAVF (8 studies; OR = 0.27; 0.09 to 0.80, I^2^ = 85.03%, low certainty), and UA-AVF (2 studies; OR = 0.24; 0.16 to 0.38, I^2^ = 0%, moderate certainty) compared with AVG, respectively.

### Functional patency

Primary functional patency did not differ at 1 year (3 studies; OR = 1.90, 95% c.i. 0.78 to 4.65; I^2^ = 84.0%; very low certainty) or 2 years (3 studies; OR = 1.37, 0.85 to 2.20; I^2^ = 61.7%; low certainty). Secondary functional patency was similar at 1 year (5 studies; OR = 0.84, 0.59 to 1.19; I^2^ = 0%; moderate certainty) but favoured AVF at 2 years (2 studies; OR = 1.54, 1.03 to 2.30; I^2^ = 0%; moderate certainty).

### Mortality

At 1 year, AVF was associated with lower mortality than AVG (6 studies; OR = 0.57, 95% c.i. 0.34 to 0.98; moderate certainty). The pooled 2-year estimate was neutral (9 studies; OR = 0.83, 0.54 to 1.25; moderate certainty).

### Revision surgery

At 1 year, revision requirements were not different (6 studies; OR = 0.54, 95% c.i. 0.26 to 1.12; I^2^ = 76.6%; low certainty).

Diabetes mellitus significantly modified the risk of revision, with a higher prevalence of diabetes associated with lower odds of AVF *versus* AVG requiring revision (coefficient −0.087; *P* < 0.001). Age (*P* = 0.17) and hypertension (*P* = 0.29) were not significant modifiers (*Table 2*).

## Discussion

In this contemporary synthesis of 63 comparative studies (357 333 patients), AVFs demonstrated superiority over AVGs at a standardized 1-year horizon with higher primary, primary-assisted, and secondary patency, fewer overall complications, and lower mortality. Differences in primary failure and revision surgery were not significant overall. These advantages were not uniform: transposed configurations showed the clearest gains for primary-assisted patency and complication reduction, and upper-arm AVFs were associated with better primary patency. Among types, BaAVF exhibited better secondary patency, whereas BrAVF carried a higher haematoma risk and BaAVF a higher stenosis risk, emphasizing that configuration matters as much as the choice of autogenous *versus* prosthetic conduit.

Previous syntheses did not either provide true comparative estimates or pooled effects without anatomical stratification. Almasri *et al*.^74^ primarily delivered incidence rates across access types including many non-comparative studies and explicitly noted that the evidence was limited for comparative effectiveness, focusing instead on population baseline risks (not pooled ORs/risk ratios/hazard ratios). Hajibandeh *et al*.^4^ pooled 15 comparative studies but restricted outcomes largely to primary failure and patency at 1, 2, and 5 years, and highlighted limited reporting of baseline characteristics and confounding by indication, precluding deeper exploration of modifiers. In contrast, the present review updates the evidence to January 2025 (63 studies), standardizes all primary syntheses at 1 year to avoid time-mixing, and provides the first comprehensive stratification by AVF type (RCAVF, BCAVF, BBAVF, BaAVF, BrAVF), AVF site (forearm *versus* upper arm), AVG site (forearm, upper arm, lower limb), and transposition status, alongside random-effects meta-regression for age, diabetes, and hypertension, and GRADE assessment. This design yields actionable, configuration-specific comparative estimates that extend beyond previous work by incorporating complications and mortality as well as patency, thereby offering clinicians a more granular evidence base for individualized access planning.

The complication advantage for AVFs, particularly lower infection and thrombosis in BaAVF/BBAVF and upper-arm placements, likely reflects the absence of prosthetic material (reduced biofilm/infective burden) and more favourable haemodynamics (larger calibre, higher flow, shorter cannulation path after superficialization). Transposition plausibly improves cannulation conditions and reduces shear-related stenosis at the puncture segment, explaining the stronger primary-assisted patency signal. Conversely, the haematoma excess with BrAVF is consistent with cannulating a deep-vein construct that often requires extensive superficialization and may be technically demanding; the stenosis signal with BaAVF aligns with a propensity for neointimal hyperplasia at swing-segment or anastomotic sites. These trade-offs argue against viewing ‘AVF’ as a single exposure.

At a standardized 1-year horizon, ‘success’ (as reported by individual studies) did not differ between AVF and AVG, whereas a 2-year advantage emerged for AVF. This pattern is consistent with the clinical trajectory of autogenous access: early maturation challenges blunt short-term success signals, but those AVFs that do mature tend to yield durable, cannulation-ready circuits by year 2. In contrast, primary failure, the mirror of early success, was not different overall at 1 year; however, non-transposed AVFs exhibited higher failure *versus* AVG, and BCAVF showed an increased failure signal in limited data. These findings support two practical points: (i) maturation risk is configuration-dependent^4^ and (ii) superficialization/transposition is not merely technical preference but a determinant of early usability^75^. Because ‘success’ and ‘primary failure’ were variably defined across studies, study-level definitions were catalogued and over-interpretation of single-study strata was avoided; the overall message is that programmatic vein mapping, selective transposition, and early endovascular optimization remain central to minimizing early AVF attrition.

AVFs demonstrated superiority over AVGs at 1 year for primary, primary-assisted, and secondary patency, with effects generally having low-to-moderate certainty. The primary-assisted advantage was most pronounced in transposed AVFs, supporting the notion that superficialized outflow segments are easier to maintain with surveillance and minor reintervention. Upper-arm AVFs were associated with higher primary patency, likely reflecting a larger calibre and more favourable haemodynamics^3,4^; BaAVF showed a signal for improved secondary patency, albeit with a trade-off of increased stenosis risk that argues for targeted duplex surveillance^3,75^. Functional patency analyses were less definitive: primary functional patency did not differ, and secondary functional patency favoured AVF only at 2 years—consistent with AVF's delayed but durable performance. Meta-regression did not show modification of patency outcomes by age or hypertension; diabetes correlated with better secondary patency for AVF *versus* AVG at the study level, a finding that may reflect centre pathways and surveillance intensity rather than biology and warrants patient-level confirmation^5^.

Overall revision requirements did not differ at 1 year between AVF and AVG, a neutral finding that likely reflects opposing mechanisms: AVGs demand more frequent thrombectomy/infection-related interventions, whereas AVFs accrue early reinterventions tied to maturation and focal stenosis. Notably, higher cohort-level diabetes prevalence was associated with fewer revisions for AVF *versus* AVG in meta-regression. This should be interpreted cautiously (ecological confounding), but it suggests that structured surveillance and early endovascular care can offset revision burden in autogenous access, even in comorbidity-dense populations^75^.

AVF use was associated with lower 1-year mortality, with the 2-year pooled estimate neutral. The near-term survival advantage plausibly tracks with the lower infection and thrombosis burden observed for AVFs (particularly BaAVF/BBAVF and upper-arm configurations) and with reduced catheter dependence^4^. The attenuation at 2 years may reflect survivor effects, crossover, or unmeasured confounding. Because overall survival was not analysed and certainty was moderate at best, these mortality signals should be viewed as supportive rather than definitive, reinforcing guideline-consistent preference for autogenous access when feasible^5^.

The overall complication burden was lower with AVFs at 1 and 2 years, driven in part by lower infection and thrombosis in specific fistula types and sites. Procedure-specific risks (for example, haematoma with BrAVF, stenosis with BaAVF) highlight the need for targeted postoperative protocols—ultrasound-guided surveillance and early endovascular optimization may further tilt outcomes towards autogenous access. Previous research^76^ has emphasized the higher infection risk associated with AVGs, which aligns with the current findings. Specifically, BBAVF, BaAVF, and RCAVF had significantly lower overall complication rates than AVGs, reinforcing the preference for these AVF types. Meanwhile, the increased risk of stenosis in BaAVF, observed in the present study, aligns with previous reports, suggesting a higher incidence of neointimal hyperplasia in this fistula type^75^.

Meta-regression identified diabetes as a significant modifier: centres/patient cohorts with higher diabetes prevalence showed better secondary patency and fewer revisions for AVF *versus* AVG, whereas age and hypertension did not modify effects. Because these are study-level analyses, the findings may reflect case-mix, surveillance intensity, and access selection pathways (ecological confounding) rather than biology. They highlight where individual patient data (IPD) meta-analysis could refine patient-specific estimates and validate effect modification.

The strengths of this study include a large, contemporary evidence base, rigorous subgrouping by site/type/transposition (including AVG site, often ignored), timepoint standardization at 1-year, preplanned meta-regression, and GRADE assessment. Limitations include substantial between-study heterogeneity, variable outcome definition, reliance on observational designs for most comparisons (residual confounding), sparse covariate reporting that constrained meta-regression beyond three variables, occasional use of digitized estimates for 1-year effects, and imprecision in several type-specific or single-study subgroups (down-weighted in narrative emphasis). Timepoint standardization, while reducing multiplicity, trades off long-horizon information; therefore 2-year effects were summarized secondarily.

At a 1-year decision horizon, the data support prioritizing AVF over AVG when anatomy permits, with transposed upper-arm configurations offering the most consistent balance of patency and safety. BaAVF's secondary-patency advantage should be paired with proactive stenosis surveillance; BrAVF is best reserved for salvage, given haematoma risk and technical demands. Where prosthetic access is unavoidable, explicit site selection (forearm/upper arm/lower limb) and infection-prevention measures are essential, given heterogeneity within AVG comparators. Research should adopt harmonized core definitions, report configuration explicitly, prefer time-to-event endpoints, and pursue IPD meta-analysis or pragmatic comparative studies embedding ultrasound mapping, structured surveillance, and predefined reintervention strategies, particularly for transposed upper-arm AVFs *versus* upper-arm grafts.

When evaluated on a standardized 1-year basis, AVFs provide superior patency, fewer complications, and lower mortality than AVGs, with configuration-specific trade-offs that can be mitigated through transposition, surveillance, and early endovascular optimization. These findings support granular, patient- and anatomy-tailored access planning and define priorities for harmonized reporting and IPD-driven comparative effectiveness work.

## Supplementary Material

zraf165_Supplementary_Data

## Data Availability

The data underlying this article are available in the article and in its online *Supplementary material.*

## References

[1] Santoro D, Benedetto F, Mondello P, Pipitò N, Barillà D, Spinelli F et al Vascular access for hemodialysis: current perspectives. Int J Nephrol Renovasc Dis 2014;7:281–294 25045278 10.2147/IJNRD.S46643 PMC4099194

[2] Allon M . Vascular access for hemodialysis patients: new data should guide decision making. Clin J Am Soc Nephrol 2019;14:954–961 30975657 10.2215/CJN.00490119 PMC6556719

[3] Bylsma LC, Gage SM, Reichert H, Dahl SLM, Lawson JH. Arteriovenous fistulae for haemodialysis: a systematic review and meta-analysis of efficacy and safety outcomes. Eur J Vasc Endovasc Surg 2017;54:513–522 28843984 10.1016/j.ejvs.2017.06.024

[4] Hajibandeh S, Burton H, Gleed P, Hajibandeh S, Wilmink T. Impact of arteriovenous fistulas versus arteriovenous grafts on vascular access performance in haemodialysis patients: a systematic review and meta-analysis. Vascular 2022;30:1021–1033 34461784 10.1177/17085381211041473

[5] Ravani P, Palmer SC, Oliver MJ, Quinn RR, MacRae JM, Tai DJ et al Associations between hemodialysis access type and clinical outcomes: a systematic review. J Am Soc Nephrol 2013;24:465–473 23431075 10.1681/ASN.2012070643 PMC3582202

[6] Scholz SS, Vukadinović D, Lauder L, Ewen S, Ukena C, Townsend RR et al Effects of arteriovenous fistula on blood pressure in patients with end-stage renal disease: a systematic meta-analysis. J Am Heart Assoc 2019;8:e011183 10.1161/JAHA.118.011183 PMC6405662 30764686

[7] Antoniou GA, Lazarides MK, Georgiadis GS, Sfyroeras GS, Nikolopoulos ES, Giannoukas AD. Lower-extremity arteriovenous access for haemodialysis: a systematic review. Eur J Vasc Endovasc Surg 2009;38:365–372 19596598 10.1016/j.ejvs.2009.06.003

[8] Page MJ, McKenzie JE, Bossuyt PM, Boutron I, Hoffmann TC, Mulrow CD et al The PRISMA 2020 statement: an updated guideline for reporting systematic reviews. BMJ 2021;372:n71 33782057 10.1136/bmj.n71 PMC8005924

[9] Wells G, Shea B, O′Connell D, Peterson J, Welch V, Losos M et al Newcastle–Ottawa Quality Assessment Scale Cohort Studies. University of Ottawa, 2014

[10] Minozzi S, Cinquini M, Gianola S, Gonzalez-Lorenzo M, Banzi R. The revised Cochrane risk of bias tool for randomized trials (RoB 2) showed low interrater reliability and challenges in its application. J Clin Epidemiol 2020;126:37–44 32562833 10.1016/j.jclinepi.2020.06.015

[11] Akoh JA . Adoption of transposed basilic vein as access for hemodialysis. Saudi J Kidney Dis Transpl 2018;29:381–385 29657207 10.4103/1319-2442.229296

[12] Arhuidese IJ, Cooper MA, Rizwan M, Nejim B, Malas MB. Vascular access for hemodialysis in the elderly. J Vasc Surg 2019;69:517–25.e1 30683199 10.1016/j.jvs.2018.05.219

[13] Arhuidese IJ, Orandi BJ, Nejim B, Malas M. Utilization, patency, and complications associated with vascular access for hemodialysis in the United States. J Vasc Surg 2018;68:1166–1174 30244924 10.1016/j.jvs.2018.01.049

[14] Ascher E, Gade P, Hingorani A, Mazzariol F, Gunduz Y, Fodera M et al Changes in the practice of angioaccess surgery: impact of dialysis outcome and quality initiative recommendations. J Vasc Surg 2000;31:84–92 10642711 10.1016/s0741-5214(00)70070-x

[15] Basel H, Ekim H, Odabasi D, Kiymaz A, Aydin C, Dostbil A. Basilic vein transposition fistulas versus prosthetic bridge grafts in patients with end-stage renal failure. Ann Vasc Surg 2011;25:634–639 21531117 10.1016/j.avsg.2011.02.016

[16] Buggs J, Tanious A, Camba V, Albertson C, Rogers E, Lahiff D et al Effective arteriovenous fistula alternative for hemodialysis access. Am J Surg 2018;216:1144–1147 30146087 10.1016/j.amjsurg.2018.08.004

[17] Chemla ES, Morsy MA. Is basilic vein transposition a real alternative to an arteriovenous bypass graft? A prospective study. Semin Dial 2008;21:352–356 18564966 10.1111/j.1525-139X.2008.00449.x

[18] Cheng CT, Chang YC, Tam KW, Yen YC, Ko YC. Comparison between transposed brachiobasilic fistula and arteriovenous graft for upper limb arteriovenous access in patients on hemodialysis. Vasc Endovascular Surg 2021;55:164–170 33228455 10.1177/1538574420969252

[19] Chue KM, Thant KZ, Luo HD, Soh YH, Ho P. Comprehensive comparison of the performance of autogenous brachial-basilic transposition arteriovenous fistula and prosthetic forearm loop arteriovenous graft in a multiethnic Asian hemodialysis population. Biomed Res Int 2016;2016:8693278 27840832 10.1155/2016/8693278 PMC5093232

[20] Coburn MC, Carney WI Jr. Comparison of basilic vein and polytetrafluoroethylene for brachial arteriovenous fistula. J Vasc Surg 1994;20:896–902; discussion 3–4 7990184 10.1016/0741-5214(94)90226-7

[21] Cui J, Steele D, Wenger J, Kawai T, Liu F, Elias N et al Hemodialysis arteriovenous fistula as first option not necessary in elderly patients. J Vasc Surg 2016;63:1326–1332 26776449 10.1016/j.jvs.2015.11.036

[22] Davoudi M, Tayebi P, Beheshtian A. Primary patency time of basilic vein transposition versus prosthetic brachioaxillary access grafts in hemodialysis patients. J Vasc Access 2013;14:111–115 23080334 10.5301/jva.5000109

[23] Dhingra RK, Young EW, Hulbert-Shearon TE, Leavey SF, Port FK. Type of vascular access and mortality in U.S. hemodialysis patients. Kidney Int 2001;60:1443–1451 11576358 10.1046/j.1523-1755.2001.00947.x

[24] Drouven JW, de Bruin C, van Roon AM, Oldenziel J, Zeebregts CJ. Outcomes of basilic vein transposition versus polytetrafluoroethylene forearm loop graft as tertiary vascular access. J Vasc Surg 2019;69:1180–1186 30528405 10.1016/j.jvs.2018.06.220

[25] Dumaine C, Espino-Hernandez G, Romann A, Luscombe R, Kiaii M. Femoral arteriovenous grafts for hemodialysis: retrospective comparison with upper extremity grafts and fistulas. Can J Kidney Health Dis 2017;4 10.1177/2054358117719747 PMC8851105 35186301

[26] Fitzgerald JT, Schanzer A, McVicar JP, Chin AI, Perez RV, Troppmann C. Upper arm arteriovenous fistula versus forearm looped arteriovenous graft for hemodialysis access: a comparative analysis. Ann Vasc Surg 2005;19:843–850 16177869 10.1007/s10016-005-7419-y

[27] Gibson KD, Gillen DL, Caps MT, Kohler TR, Sherrard DJ, Stehman-Breen CO. Vascular access survival and incidence of revisions: a comparison of prosthetic grafts, simple autogenous fistulas, and venous transposition fistulas from the United States renal data system dialysis morbidity and mortality study. J Vasc Surg 2001;34:694–700 11668326 10.1067/mva.2001.117890

[28] Harms JC, Rangarajan S, Young CJ, Barker-Finkel J, Allon M. Outcomes of arteriovenous fistulas and grafts with or without intervention before successful use. J Vasc Surg 2016;64:155–162 27066945 10.1016/j.jvs.2016.02.033 PMC4925201

[29] Hicks CW, Wang P, Kernodle A, Lum YW, Black JH 3rd, Makary MA. Assessment of use of arteriovenous graft vs arteriovenous fistula for first-time permanent hemodialysis access. JAMA Surg 2019;154:844–851 31188411 10.1001/jamasurg.2019.1736 PMC6563590

[30] Itoga NK, Virgin-Downy W, Mell MW. Forearm loop arteriovenous grafts preserve and may create new upper arm access sites. J Vasc Access 2019;20:691–696 31006339 10.1177/1129729819835137 PMC8439284

[31] Jadlowiec CC, Mannion EM, Lavallee M, Brown MG. Hemodialysis access in the elderly: outcomes among patients older than seventy. Ann Vasc Surg 2016;31:77–84 26616499 10.1016/j.avsg.2015.08.013

[32] Kakkos SK, Andrzejewski T, Haddad JA, Haddad GK, Reddy DJ, Nypaver TJ et al Equivalent secondary patency rates of upper extremity Vectra vascular access grafts and transposed brachial-basilic fistulas with aggressive access surveillance and endovascular treatment. J Vasc Surg 2008;47:407–414 18155874 10.1016/j.jvs.2007.09.061

[33] Kalman PG, Pope M, Bhola C, Richardson R, Sniderman KW. A practical approach to vascular access for hemodialysis and predictors of success. J Vasc Surg 1999;30:727–733 10514212 10.1016/s0741-5214(99)70112-6

[34] Kawecka A, Debska-Slizień A, Prajs J, Król E, Zdrojewski Z, Przekwas M et al Remarks on surgical strategy in creating vascular access for hemodialysis: 18 years of one center's experience. Ann Vasc Surg 2005;19:590–598 15995788 10.1007/s10016-005-5020-z

[35] Keuter XH, De Smet AA, Kessels AG, van der Sande FM, Welten RJ, Tordoir JH. A randomized multicenter study of the outcome of brachial-basilic arteriovenous fistula and prosthetic brachial-antecubital forearm loop as vascular access for hemodialysis. J Vasc Surg 2008;47:395–401 18155872 10.1016/j.jvs.2007.09.063

[36] Kim DS, Kim SW, Kim JC, Cho JH, Kong JH, Park CR. Clinical analysis of hemodialysis vascular access: comparison of autogenous arteriovenous fistula & arteriovenous prosthetic graft. Korean J Thorac Cardiovasc Surg 2011;44:25–31 22263120 10.5090/kjtcs.2011.44.1.25 PMC3249269

[37] Lee CH, Ko PJ, Liu YH, Hsieh HC, Liu HP. Brachiobasilic fistula as a secondary access procedure: an alternative to a dialysis prosthetic graft. Chang Gung Med J 2004;27:816–823 15796257

[38] Lee T, Barker J, Allon M. Comparison of survival of upper arm arteriovenous fistulas and grafts after failed forearm fistula. J Am Soc Nephrol 2007;18:1936–1941 17475812 10.1681/ASN.2006101119

[39] Lioupis C, Mistry H, Rix T, Chandak P, Tyrrell M, Valenti D. Comparison among transposed brachiobasilic, brachiobrachial arteriovenous fistulas and Flixene™ vascular graft. J Vasc Access 2011;12:36–44 21104668 10.5301/jva.2010.6065

[40] Marques G, Sadaghianloo N, Fouilhé L, Jean-Baptiste E, Declemy S, Clément C et al Higher patency of transposed brachio-basilic arteriovenous fistulas compared to brachio-axillary grafts for hemodialysis patients. J Vasc Access 2015;16:486–492 26109547 10.5301/jva.5000433

[41] Matsuura JH, Rosenthal D, Clark M, Shuler FW, Kirby L, Shotwell M et al Transposed basilic vein versus polytetrafluorethylene for brachial-axillary arteriovenous fistulas. Am J Surg 1998;176:219–221 9737637 10.1016/s0002-9610(98)00122-6

[42] Maya ID, O'Neal JC, Young CJ, Barker-Finkel J, Allon M. Outcomes of brachiocephalic fistulas, transposed brachiobasilic fistulas, and upper arm grafts. Clin J Am Soc Nephrol 2009;4:86–92 18945990 10.2215/CJN.02910608 PMC2615695

[43] Milburn JA, Lo ST, Szucs ZJ, Humphrey A, Macaulay EM. Transposed brachiobasilic fistula or PTFE arm graft—alternative or complementary? J Vasc Access 2008;9:117–121 18609527

[44] Morosetti M, Cipriani S, Dominijanni S, Pisani G, Frattarelli D, Bruno F. Basilic vein transposition versus biosynthetic prosthesis as vascular access for hemodialysis. J Vasc Surg 2011;54:1713–1719 21803519 10.1016/j.jvs.2011.06.030

[45] Oliver MJ, McCann RL, Indridason OS, Butterly DW, Schwab SJ. Comparison of transposed brachiobasilic fistulas to upper arm grafts and brachiocephalic fistulas. Kidney Int 2001;60:1532–1539 11576369 10.1046/j.1523-1755.2001.00956.x

[46] Park HS, Kim WJ, Kim YK, Kim HW, Choi BS, Park CW et al Comparison of outcomes with arteriovenous fistula and arteriovenous graft for vascular access in hemodialysis: a prospective cohort study. Am J Nephrol 2016;43:120–128 27022896 10.1159/000444889

[47] Pflederer TA, Kwok S, Ketel BL, Pilgram T. A comparison of transposed brachiobasilic fistulae with nontransposed fistulae and grafts in the Fistula First era. Semin dial 2008;21:357–363 18564963 10.1111/j.1525-139X.2008.00451.x

[48] Pham XD, Kim JJ, Ihenachor EJ, Parrish AB, Bleck JD, Kaji AH et al A comparison of brachial artery-brachial vein arteriovenous fistulas with arteriovenous grafts in patients with poor superficial venous anatomy. J Vasc Surg 2017;65:444–451 27986484 10.1016/j.jvs.2016.09.037

[49] Snyder DC, Clericuzio CP, Stringer A, May W. Comparison of outcomes of arteriovenous grafts and fistulas at a single veterans’ affairs medical center. Am J Surg 2008;196:641–646 18823616 10.1016/j.amjsurg.2008.07.013

[50] Srikuea K, Prajumsukh K, Orrapin S, Benyakorn T, Ho P, Rerkasem K et al One-staged brachial-basilic vein transposition versus arm straight arteriovenous graft for hemodialysis. Vascular 2025;33:335–341 38576306 10.1177/17085381241245068

[51] Tayebi P, Kazemzadeh G, Modaghegh MHS, Kamyar MM, Ravari H. Brachio-basilic upper arm transposition fistulas vs. prosthetic brachio-axillary vascular access grafts-which one is preferred for hemodialysis? Hemodial Int 2020;24:182–187 32052592 10.1111/hdi.12817

[52] Torina PJ, Westheimer EF, Schanzer HR. Brachial vein transposition arteriovenous fistula: is it an acceptable option for chronic dialysis vascular access? J Vasc Access 2008;9:39–44 18379979

[53] Voorzaat BM, Janmaat CJ, van der Bogt KEA, Dekker FW, Rotmans JI. Patency outcomes of arteriovenous fistulas and grafts for hemodialysis access: a trade-off between nonmaturation and long-term complications. Kidney360 2020;1:916–924 35369548 10.34067/KID.0000462020 PMC8815607

[54] Weale AR, Bevis P, Neary WD, Lear PA, Mitchell DC. A comparison between transposed brachiobasilic arteriovenous fistulas and prosthetic brachioaxillary access grafts for vascular access for hemodialysis. J Vasc Surg 2007;46:997–1004 17980286 10.1016/j.jvs.2007.07.023

[55] Woo K, Farber A, Doros G, Killeen K, Kohanzadeh S. Evaluation of the efficacy of the transposed upper arm arteriovenous fistula: a single institutional review of 190 basilic and cephalic vein transposition procedures. J Vasc Surg 2007;46:94–99; discussion 100 17543490 10.1016/j.jvs.2007.02.057

[56] Yan Y, Clark TW, Mondschein JI, Shlansky-Goldberg RD, Dagli MS, Soulen MC et al Outcomes of percutaneous interventions in transposed hemodialysis fistulas compared with nontransposed fistulas and grafts. J Vasc Interv Radiol 2013;24:1765–1772; quiz 73 24409470 10.1016/j.jvir.2013.08.025

[57] Yuo TH, Chaer RA, Dillavou ED, Leers SA, Makaroun MS. Patients started on hemodialysis with tunneled dialysis catheter have similar survival after arteriovenous fistula and arteriovenous graft creation. J Vasc Surg 2015;62:1590–1597.e2 26372193 10.1016/j.jvs.2015.07.076 PMC4659732

[58] Allemang MT, Schmotzer B, Wong VL, Lakin RO, Woodside KJ, Schulak JA et al Arteriovenous grafts have higher secondary patency in the short term compared with autologous fistulae. Am J Surg 2014;208:800–805 24811929 10.1016/j.amjsurg.2014.01.010

[59] Asif A, Gadalean FN, Merrill D, Cherla G, Cipleu CD, Epstein DL et al Inflow stenosis in arteriovenous fistulas and grafts: a multicenter, prospective study. Kidney Int 2005;67:1986–1992 15840048 10.1111/j.1523-1755.2005.00299.x

[60] Bacchini G, Del Vecchio L, Andrulli S, Pontoriero G, Locatelli F. Survival of prosthetic grafts of different materials after impairment of a native arteriovenous fistula in hemodialysis patients. ASAIO J 2001;47:30–33 11199311 10.1097/00002480-200101000-00008

[61] Charlton-Ouw KM, Nosrati N, Miller CC 3rd, Coogan SM, Safi HJ, Azizzadeh A. Outcomes of arteriovenous fistulae compared with heparin-bonded and conventional grafts for hemodialysis access. J Vasc Access 2012;13:163–167 21983827 10.5301/JVA.2011.8715

[62] Danese MD, Liu Z, Griffiths RI, Dylan M, Yu H-T, Dubois AR et al Catheter use is high even among hemodialysis patients with a fistula or graft. Kidney Int 2006;70:1482–1485 16941025 10.1038/sj.ki.5001786

[63] Falk A, Maya ID, Yevzlin AS; RESCUE Investigators. A prospective, randomized study of an expanded polytetrafluoroethylene stent graft versus balloon angioplasty for in-stent restenosis in arteriovenous grafts and fistulae: two-year results of the RESCUE study. J Vasc Interv Radiol 2016;27:1465–1476 27514445 10.1016/j.jvir.2016.06.014

[64] Galal AM, Ismail MA, Abdrabo MS, Mahmoud AK. Saphenous vein versus synthetic graft in arteriovenous fistula for hemodialysis in patient with inaccessible veins. Int Angiol 2024;43:615–620 39666272 10.23736/S0392-9590.24.05287-8

[65] Ghaffarian AA, Al-Dulaimi R, Kraiss LW, Sarfati M, Griffin CL, Smith BK et al Clinical effectiveness of open thrombectomy for thrombosed autogenous arteriovenous fistulas and grafts. J Vasc Surg 2018;68:189–196 29526376 10.1016/j.jvs.2017.12.050

[66] Kherlakian GM, Roedershelmer LR, Arbaugh JJ, Newmark KJ, King LR. Comparison of autogenous fistula versus expanded polytetrafluoroethylene graft fistula for angioaccess in hemodialysis. Am J Surg 1986;152:238–243 3740363 10.1016/0002-9610(86)90249-7

[67] Kim H, Ahn S, Kim M, Chung CTY, Choi KW, Ko H et al Comparison between autogenous brachial–brachial upper-arm elevated direct arteriovenous fistulas and prosthetic brachial-antecubital indirect forearm arteriovenous grafts. J Vasc Access 2022;23:936–945 34058911 10.1177/11297298211018020

[68] Ladenheim ED, Lulic D, Lum C, Agrawal S. Primary and secondary patencies of transposed femoral vein fistulas are significantly greater than with the HeRO graft. J Vasc Access 2017;18:232–237 28478626 10.5301/jva.5000697

[69] Lok CE, Sontrop JM, Tomlinson G, Rajan D, Cattral M, Oreopoulos G et al Cumulative patency of contemporary fistulas versus grafts (2000–2010). Clin J Am Soc Nephrol 2013;8:810–818 23371955 10.2215/CJN.00730112 PMC3641610

[70] Simoni E, Blitz L, Lookstein R. Outcomes of AngioJet® thrombectomy in hemodialysis vascular access grafts and fistulas: PEARL I registry. J Vasc Access 2013;14:72–76 22865534 10.5301/jva.5000102

[71] Staramos DN, Lazarides MK, Tzilalis VD, Ekonomou CS, Simopoulos CE, Dayantas JN. Patency of autologous and prosthetic arteriovenous fistulas in elderly patients. Eur J Surg 2000;166:777–781 11071164 10.1080/110241500447407

[72] Wang S, Wang MS. Successful use of partial aneurysmectomy and repair approach for managing complications of arteriovenous fistulas and grafts. J Vasc Surg 2017;66:545–553 28579291 10.1016/j.jvs.2017.03.429

[73] Jadlowiec CC, Lavallee M, Mannion EM, Brown MG. An outcomes comparison of native arteriovenous fistulae, polytetrafluorethylene grafts, and cryopreserved vein allografts. Ann Vasc Surg 2015;29:1642–1647 26319146 10.1016/j.avsg.2015.07.009

[74] Almasri J, Alsawas M, Mainou M, Mustafa RA, Wang Z, Woo K et al Outcomes of vascular access for hemodialysis: a systematic review and meta-analysis. J Vasc Surg 2016;64:236–243 27345510 10.1016/j.jvs.2016.01.053

[75] Al-Jaishi AA, Liu AR, Lok CE, Zhang JC, Moist LM. Complications of the arteriovenous fistula: a systematic review. J Am Soc Nephrol 2017;28:1839–1850 28031406 10.1681/ASN.2016040412 PMC5461784

[76] Tanner N, Da Silva A. Medical adjuvant treatment to improve the patency of arteriovenous fistulae and grafts: a systematic review and meta-analysis. Eur J Vasc Endovasc Surg 2016;52:243–252 27289558 10.1016/j.ejvs.2016.04.016

