# Supplementary material for: Comparative outcomes of arteriovenous fistulas and grafts in haemodialysis: meta-analysis with subgroup analysis by fistula type and transposition status
Source: BJS Open. 2026 Apr 1;10(2):zraf165. doi: 10.1093/bjsopen/zraf165 (PMC13042302; doi:10.1093/bjsopen/zraf165)
Supplement: zraf165_Supplementary_Data [file zraf165_supplementary_data.docx]

**Comparative Outcomes of Arteriovenous Fistulas and Grafts in** **Haemodialysis: Systematic Review and Meta Analysis with Subgroup Analysis by Fistula Type and Transposition Status**

Jielun Yang1, Wenhsing Yang2, Leijuan Xiao1, Runzhang Zhu1, Xiaofeng Li1, Erqing Xiang1, Hongying Wang1, Jizhuang Lou1, Zhanhui Gao1*

^1^Department of Nephrology, Nanjing BenQ Medical Center, The Affiliated BenQ Hospital of Nanjing Medical University, Nanjing, Jiangsu Province, 210019, China

^2^New York City College of Technology, 11201, USA

**Corresponding author.** Zhanhui Gao. Department of Nephrology, Nanjing BenQ Medical Center, The Affiliated BenQ Hospital of Nanjing Medical University, Nanjing, Jiangsu Province, 210019, China**.**

**Supplementary Materials - Index**

| **Supplementary Figures and Tables** |  |
| --- | --- |
| Supplementary Table 1. The search syntax employed in the database search | *page 3* |
| Supplementary Table 2. Baseline characteristics of studies comparing AVF to AVG in hemodialysis patients | *page 4* |
| Supplementary Table 3. A summary of the medical conditions of patients receiving either AVF or AVG during hemodialysis | *page 12* |
| Supplementary Table 4. A summary of the methodological quality of included observational studies using the NIH tool | *page 18* |
| Supplementary Table 5. A summary of complications reported with AVF vs. AVG use in hemodialysis patient stratified by complication type and AVF type | *page 21* |
| Figure S1. Primary patency by time. | *page 23* |
| Figure S2. Primary patency by AVF transposition status (1 year). | *page 24* |
| Figure S3. Primary patency by AVF type (1 year). | *page 25* |
| Figure S4. Primary patency by AVF site (1 year). | *page 26* |
| Figure S5. Primary patency by AVG site (1 year). | *page 27* |
| Figure S6. Primary-assisted patency by time. | *page 28* |
| Figure S7. Primary-assisted patency by AVF type (1 year). | *page 29* |
| Figure S8. Primary-assisted patency by AVF site (1 year). | *page 30* |
| Figure S9. Primary-assisted patency by AVG site (1 year). | *page 31* |
| Figure S10. Secondary patency by time. | *page 32* |
| Figure S11. Secondary patency by AVF transposition status (1 year). | *page 33* |
| Figure S12. Secondary patency by AVF site (1 year). | *page 34* |
| Figure S13. Secondary patency by AVG site (1 year). | *page 35* |
| Figure S14. Primary failure by time. | *page 36* |
| Figure S15. Primary failure by AVF transposition status (1 year). | *page 37* |
| Figure S16. Primary failure by AVF type (1 year). | *page 38* |
| Figure S17. Primary failure by AVF site (1 year). | *page 39* |
| Figure S18. Primary failure by AVG site (1 year). | *page 40* |
| Figure S19. Success by time. | *page 41* |
| Figure S20. Success by AVF transposition status (1 year). | *page 42* |
| Figure S21. Success by AVF type (1 year). | *page 43* |
| Figure S22. Success by AVF site (1 year). | *page 44* |
| Figure S23. Success by AVG site (1 year). | *page 45* |
| Figure S24. Complications by time. | *page 46* |
| Figure S25. Complications by AVF type (1 year). | *page 47* |
| Figure S26. Complications by AVF site (1 year). | *page 48* |
| Figure S27. Complications by AVG site (1 year). | *page 49* |
| Figure S28. Primary functional patency by time. | *Page 50* |
| Figure S29. Secondary functional patency by time. | *page 51* |
| Figure S30. Revision surgery by time. | *page 52* |
| **References** | *page 53* |

**Supplementary Figures and Tables**

**Supplementary Table 1.** The search syntax employed in the database search

| PubMed (n=2269) |
| --- |
| (graft*[tiab] OR AVG[tiab]) AND (fistula*[tiab] OR AVF[tiab]) AND (dialysis[tiab] OR hemodialysis[tiab]) AND (patients[tiab] OR cases[tiab] OR individuals[tiab] OR people[tiab]) NOT ("systematic review"[tiab] OR "meta-analysis"[tiab] OR "meta analysis"[tiab]) |
| Web of Science (n=1972) |
| (AB=graft* OR AB=AVG) AND (AB=fistula* OR AB=AVF) AND (AB=dialysis OR AB=hemodialysis) AND (AB=patients OR AB=cases OR AB=individuals OR AB=people) NOT (AB="systematic review" OR AB="meta-analysis" OR AB="meta analysis") |
| CENTRAL (n=311) |
| (graft OR AVG) AND (fistula OR AVF) AND (dialysis OR hemodialysis) AND (patients OR cases OR individuals OR people) NOT ("systematic review" OR "meta-analysis" OR "meta analysis") |
| Scopus (n=4403) |
| (TITLE-ABS-KEY (graft*) OR TITLE-ABS-KEY (AVG)) AND (TITLE-ABS-KEY (fistula*) OR TITLE-ABS-KEY (AVF)) AND (TITLE-ABS-KEY (dialysis) OR TITLE-ABS-KEY (hemodialysis)) AND (TITLE-ABS-KEY (patients) OR TITLE-ABS-KEY (cases) OR TITLE-ABS-KEY (individuals) OR TITLE-ABS-KEY (people)) AND NOT (TITLE-ABS-KEY ("systematic review") OR TITLE-ABS-KEY ("meta-analysis") OR TITLE-ABS-KEY ("meta analysis")) |
| Total (63 included studies)^1-63^ |

**Supplementary Table 2.** Baseline characteristics of studies comparing AVF to AVG in hemodialysis patients

| *Study ID* | *Country* | *Design* | *Arm* | AVF/AVG site | *Sample* | *Age; m (SD)* | *Gender (M/F)* | *History of previous AVF/AVG; n (%)* | *First access; n (%)* | *Time on dialysis (day); m (SD)* | *Previous catheter access; n (%)* |
| --- | --- | --- | --- | --- | --- | --- | --- | --- | --- | --- | --- |
|  |  |  |  |  |  |  |  |  |  |  |  |
| Coburn 1994 | Canada | RC | BaAVF | UA | 59 | 64.1 | 30/29 | - | - | - | - |
|  |  |  | AVG | UA | 47 | 65.7 | 15/32 | - | - | - | - |
| Matsuura1998 | USA | RC | BaAVF | UA | 30 | 59 | 14/16 |  |  |  |  |
|  |  |  | AVG | UA | 68 | 62 | 36/32 |  |  |  |  |
| Ascher 1999 | USA | RC | nAVF | - | 191 | 69(0.59) | 133/114 | - | - | - | - |
|  |  |  | AVG | FA/UA | 56 |  |  | - | - | - | - |
| Kalman 1999 | Canada | RC | AVF | - | 235 | 59 | 230/154 | - | - | - | - |
|  |  |  | AVG | FA/UA/LL | 231 |  |  | - | - | - | - |
| Gibson 2001 | USA | PC | nAVF | - | 673 | 66(15) | 1182/986 | 2247(100) | 1566 (69.7) | - | - |
|  |  |  | AVG | - | 1574 |  |  |  |  | - | - |
| OLIVER 2001 | USA | RC | BBAVF | UA | 59 | 53 | 59/0 | 39(66.2) | 22(37) | 683 | - |
|  |  |  | BCAVF | UA | 60 | 59 | 56/0 | 53(66.3) | 27(34) | 798 | - |
|  |  |  | AVG | UA | 80 | 59 | 45/35 | 30(53.6) | 40(71) | 198 | - |
| Dhingra 2001 | USA | RC | AVF | - | 1340 | 52 | 892/448 | - | - | - | - |
|  |  |  | AVG | - | 3129 | 59 | 1389/1740 | - | - | - | - |
| Lee 2004 | USA | RC | BBAVF | UA | 20 | 59.2(19) | 7/13 | - | - | - | - |
|  |  |  | AVG | UA | 50 | 69 (11.75) | 20/30 | - | - | - | - |
| Fitzgerald 2005 | USA | RC | nAVF | - | 86 | 56(16) | 48/38 | 5 (5.8) | - | 5.7(6.9) | 27 (31) |
|  |  |  | AVG | FA | 60 | 55(15) | 20/40 | - | - | 8.9(15) | 19 (32) |
| Kawecka 2005 | Poland | RC | BBAVF | UA | 85 | 43.7 (17.7) / children (13.2 (3.2) | 399/323 | 64(17.1) | 21(3.4) | - | - |
|  |  |  | BCAVF | UA | 143 |  |  | 69(18.5) | 74(12) | - | - |
|  |  |  | RCAVF | FA | 540 |  |  | 73(19.5) | 467(75.6) | - | - |
|  |  |  | AVG | UA | 90 |  |  |  |  |  |  |
| Keuter 2007 | The Netherlands | RCT | BBAVF | UA | 52 | 60 | 26/26 | - | 27 (52) | - | 33 (63) |
|  |  |  | AVG | FA | 53 | 66 | 30/23 | - | 8(15) | - | 29 (55) |
| Kakkos 2007 | USA | RC | BBAVF | UA | 41 | 59.4(16.5) | 18/23 | 32 (78) | - | - | - |
|  |  |  | AVG | - | 76 | 61.6(16) | 39/37 | 47 (62) | - | - | - |
| Lee 2007 | USA | RC | AVF | - | 63 | 56(13) | 36/23 | - | - | - | - |
|  |  |  | AVG | UA | 51 | 55(14) | 21/30 | - | - | - | - |
| Woo 2007 | USA | RC | tAVF | - | 190 | 63(18) | 120/70 | - | - | - | 37 (19) |
|  |  |  | AVG | UA | 139 | 67(16) | 78/61 | - | - | - | 53 (32) |
| Weale 2007 | UK | RC | BBAVF | UA | 71 | 61(18.5) | 33/38 | 16 (22.5) | - | - | - |
|  |  |  | AVG | UA | 104 | 64.57(16.22) | 50/64 | 34 (29.8) | - | - | - |
| Chemla 2008 | UK | PC | BaAVF | UA | 34 | 62 | 12/22 | - | - | - | - |
|  |  |  | AVG | UA | 42 | 42 | 22/20 | - | - | - | - |
| MILBURN 2008 | Scotland | PC | BBAVF | UA | 52 | 65.1(17) | 32/20 | 27 (52) | 25 (48) | - | - |
|  |  |  | AVG | UA | 39 | 62.1(14) | 17/22 | 31 (79) | 8 (21) | - | - |
| Pflederer 2008 | USA | RC | Transposed | - | 161 | 63.3(15.3) | 98/63 | - | 87(54) | - | - |
|  |  |  | Non-transposed | - | 321 | 64.5(15.1) | 210/111 | - | 178(67) | - | - |
|  |  |  | RCAVF | FA | 118 | 63.5(15.8) | 80/38 | - | 65(70) | - | - |
|  |  |  | BCAVF | UA | 203 | 65(14.7) | 73/130 | - | 113(65) | - | - |
|  |  |  | AVG | FA/UA/LL | 285 | 65.1(15.7) | 127/158 | - | 185(65) | - | - |
| Snyder 2008 | USA | RC | AVG | - | 53 | 60.2 | 52/1 | - | - | - | - |
|  |  |  | AVF | FA/UA | 22 | 60.6 | 20/2 | - | - | - | - |
| TORINA 2008 | USA | RC | BCAVF | UA | 13 | 67(8.75) | 7/6 | - | - | - | 9 (69%) |
|  |  |  | BVAVF | UA | 42 | 62(16.5) | 14/28 | - | - | - | 33 (79%) |
|  |  |  | AVG | FA/UA | 94 | 62(16.25) | 37/57 | - | - | - | 73 (78%) |
| Maya 2009 | USA | PC | BBAVF | UA | 67 | 56(15) | 35/32 | - | - | - | 31 (46%) |
|  |  |  | BCAVF | UA | 322 | 56(14) | 155/167 | - | - | - | 146 (45%) |
|  |  |  | AVG | UA | 289 | 56(14) | 122/167 | - | - | - | 163 (56%) |
| Basel 2011 | Turkey | RC | BaAVF | UA | 108 | 55 | - | - | - | - | - |
|  |  |  | PBG | UA | 49 | 55 | - | - | - | - | - |
| Lioupis 2011 | UK | PC | BBAVF | UA | 45 | 62(13.25) | 24/21 | - | - | - | - |
|  |  |  | BrAVF | UA | 15 | 60(10.75) | 8/7 | - | - | - | - |
|  |  |  | AVG | FA/UA | 48 | 59(13.5) | 31/17 | - | - | - | - |
| Kim 2011 | Korea | RC | AVF | - | 92 | 55.4(13.7) | 57/35 | - | - | - | - |
|  |  |  | AVG | FA/UA | 37 | 59.8(10.4) | 16/21 | - | - | - | - |
| Morosetti 2011 | Italy | RCT | AVG | - | 27 | 69(12) | 8/19 | - | - | - | - |
|  |  |  | AVF | - | 30 | 63(18) | 14/16 | - | - | - | - |
| Yan 2013 | USA | RC | tAVF | - | 195 | 57 (16) | 103/92 | - | - | - | - |
|  |  |  | NAVF | - | 205 | 57 (15) | 60/145 | - | - | - | - |
|  |  |  | AVG | FA/UA/LL | 191 | 59 (15) | 126/65 | - | - | - | - |
| Davoudi 2013 | Iran | RCT | BaAVF | UA | 30 | 64.93 (15.42) | 8/22 | - | - | - | - |
|  |  |  | AVG | UA | 30 | 60.10 (16.53) | 13/17 | - | - | - | - |
| Cui 2015 | USA | RC | AVF | - | 138 | - | 90/48 | - | - | - | - |
|  |  |  | AVG | FA/UA | 44 | - | 25/19 | - | - | - | - |
| Pham 2015 | USA | RC | BVAVF | - | 29 | 55.67 (7.02) | 15/14 | - | 8 (28) | - | 10 (34) |
|  |  |  | AVG | FA/UA | 32 | 44.7(13.97) | 11/21 | - | 16 (50) | - | 28 (88) |
| Jadlowiec 2015 | USA | RC | AVF | - | 149 | 78.8 (5.3) | 89/60 | - | - | - | - |
|  |  |  | AVG | - | 37 | 78.0 (6.2) | 15/22 | - | - | - | - |
| Marques 2015 | France | RC | BBAVF | UA | 136 | 63.4(16.4) | 83/53 | 75(55) | - | - | - |
|  |  |  | AVG | UA | 102 | 68.3 (15.482) | 44/58 | 71(70) | - | - | - |
| Yuo 2015 | USA | RC | AVF | - | 31493 | 68.9 (12.5) | 18360/13133 | - | - | - | - |
|  |  |  | AVG | FA/UA | 10492 | 70.2 (12.0) | 4470/6022 | - | - | - | - |
| Chue 2016 | Singapore | RC | BBAVF | UA | 81 | 59.14 | 49/32 | - | - | - | - |
|  |  |  | AVG | FA | 41 | 57.24 | 23/18 | - | - | - | - |
| Harms 2016 | USA | RC | AVF | - | 518 | - | 329/271 | - | - | - | - |
|  |  |  | AVG | FA/UA/LL | 558 | - |  | - | - | - | - |
| Park 2016 | Korea | PC | AVF | - | 747 | 57(13) | 482/265 | - | - | - | - |
|  |  |  | AVG | - | 199 | 61(12) | 112/87 | - | - | - | - |
| Buggs 2017 | USA | RC | AVF | - | 416 | 60 (15.6) | 253/163 | - | - | - | - |
|  |  |  | AVG | - | 58 | 60 (15.2) | 22/36 | - | - | - | - |
| Dumaine 2017 | Canada | RC | BBAVF | UA | 87 | 67(17.3) | 57/30 | 28 (32.2%) | - | - | - |
|  |  |  | BCAVF | UA | 173 | 68.3(14.2) | 107/66 | 76 (43.9%) | - | - | - |
|  |  |  | RCAVF | FA | 69 | 66.3(13.63) | 51/18 | 31 (44.9%) | - | - | - |
|  |  |  | SBF (snuffbox) | - | 55 | 65.67(21.3) | 37/18 | 36 (65.5%) | - | - | - |
|  |  |  | AVG | LL | 419 | 67.67(14.88) | 267/152 | 173 (41.3%) | - | - | - |
| Akoh 2018 | UK | RC | AVF | - | 19 | 64.44 (16.3) | 12/7 | - | - | - | - |
|  |  |  | AVG | UA | 18 | 76.16(15.5) | 11/7 | - | - | - | - |
| Arhuidese 2018 | USA | RC | AVF | - | 73883 | 64.1 (15.1) | 47729/26154 | - | - | - | - |
|  |  |  | AVG | - | 16533 | 65.5(14.9) | 7374/9159 | - | - | - | - |
| Drouven 2018 | The Netherlands | RC | BaAVF | UA | 55 | 58.3 (13.9) | 30/25 | - | 30 (54.5) | - | 38 (69.1) |
|  |  |  | AVG | FA | 75 | 62.6 (14.9) | 39/36 | - | 35 (46.7) | - | 55 (73.3) |
| Arhuidese 2019 | USA | RC | AVF | - | 19173 | 82.0 (6.4) | 12,422/6751 | - | - | - | - |
|  |  |  | AVG | - | 4840 | 82.2(6.3) | 2199/2641 | - | - | - | - |
| Hicks 2019 | USA | RC | AVF | - | 66489 | 63.7(63) | 38950/27539 | - | - | - | - |
|  |  |  | AVG | - | 18831 | 63.67(60.1) | 8420/10411 | - | - | - | - |
| Itoga 2019 | USA | RC | AVF | - | 278 | 57.7(16.6) | 175/103 | - | - | - | 30 (11) |
|  |  |  | AVG | FA | 38 | 65.2(13.8) | 25/13 | - | - | - | 4 (11) |
| Cheng 2020 | Taiwan | RC | BBAVF | UA | 20 | 67.50 (9.72) | 8/12 | - | 19 (95.0) | - | 19 (95.0) |
|  |  |  | AVG | FA/UA | 119 | 68.35 (11.77) | 53/66 | - | 25 (22.1) | - | 95 (79.8) |
| Tayebi 2020 | Iran | RCT | BBAVF | UA | 30 | 66.3(12.05) | 10/20 | - | - | - | - |
|  |  |  | AVG | UA | 30 | 63.4(17.37) | 18/12 | - | - | - | - |
| Voorzaat 2020 | The Netherlands | RC | RcAVF | FA | 587 | 62.76(15.0) | 392/195 | - | - | - | - |
|  |  |  | AVF | UA | 378 | 63.4(14.4) | 199/179 | - | - | - | - |
|  |  |  | AVG | FA/UA | 106 | 65.4(14.0) | 44/62 | - | - | - | - |
| Srikuea 2024 | Thailand | RC | BBAVF | UA | 28 | 61 (13) | 15/13 | - | 11 (39) | - | 18 (64) |
|  |  |  | AVG | UA | 74 | 63 (14) | 40/34 | - | 7 (9) | - | 44 (59) |
| Allemang 2014 | USA | RC | AVF | - | 390 | 58.3(16.4) | 143/128 | - | 90(33.2) | - | 151(55.7) |
|  |  |  | AVG | - | 265 | 60.8(16.6) | 51/102 | - | 109(71.2) | - | 126(82.3) |
| Asif 2005 | USA | PC | AVF | - | 101 | 55.9 (12.9) | 63/95 | 81 (51%) | - | - | - |
|  |  |  | AVG | LL/UA | 122 |  |  |  | - | - | - |
| Bacchini 2001 | Italy | RC | AVF | - | 404 | 65(14) | 172/232 | - | - | 50(53) | - |
|  |  |  | AVG | UA | 53 | 68(11) | 18/35 | - | - | 70(65) | - |
| Charlton-Ouw 2012 | USA | RC | BaAVF | UA | 64 | 53.2 (19.2) | 34/30 | - | - | - | - |
|  |  |  | AVG (synthetic) | UA | 21 | 51.9(12.4) | 16/15 | - | - | - | - |
|  |  |  | Heparin-bonded AVG | UA | 21 | 61.7 (15.6) | 10/11 | - | - | - | - |
| Danese 2006 | USA | RC | AVF | - | 22339 | - | 15413/6925 | 6702(30) | - | - | - |
|  |  |  | AVG | - | 70316 | - | 37267/33049 | 21095(30) | - | - | - |
| Falk 2016 | USA | RCT | AVF | - | 143 | 62.6 (13.6) | 73/70 | - | - |  | - |
|  |  |  | AVG | FA/UA | 132 | 61.1 (13.5) | 68/64 | - | - |  | - |
| Galal 2024 | Egypt | RCT | AVF | LL | 25 | 50.96(4.6) | 14/11 | - | - | - | - |
|  |  |  | AVG | - | 25 | 53.4(5.12) | 18/7 | - | - | - | - |
| Ghaffarian 2018 | USA | RC | AVF UA | UA | 52 | 51.4 (18.6) | 23/29 | - | 17 (33) | - | - |
|  |  |  | AVF FA | FA | 16 | 48.5 (17.9) | 12/4 | - | 5 (31) | - | - |
|  |  |  | AVG | FA/UA | 136 | 53.1 (19.1) | 42/94 | - | 61 (45) | - | - |
| Jadlowiec 2015 | USA | RC | AVF | - | 70 | 58.1 (10.2) | 34/36 | - | - | - | - |
|  |  |  | AVG | - | 70 | 58.9 (9.0) | 37/33 | - | - | - | - |
| kherlakian 1986 | USA | RC | AVF | - | 100 | 50 | 61/39 | - | - | - | - |
|  |  |  | AVG | FA/UA | 100 | 53 | 42/58 | - | - | - | - |
| kim 2021 | Korea | RC | BrAVF | UA | 19 | 61.1(13.1) | 9/10 | 7 (36.8%) | 1 (5.3%) | - | - |
|  |  |  | AVG | FA | 53 | 67.7(13.9) | 30/23 | 6 (11.3%) | 19 (35.8%) | - | - |
| ladenheim 2017 | USA | RC | AVF | - | 18 | 54.0 (13.7) | 13/5 | - | - | - | - |
|  |  |  | AVG | LL | 40 | 55.9 (14.6) | 17/23 | - | - | - | - |
| Lok 2013 | Canada | RC | AVF | - | 1012 | 59.8 (16.4) | 682/330 | - | - | 301.5(402.6) | - |
|  |  |  | AVG | FA/UA | 128 | 61.3 (14.9) | 52/76 | - | - | 299.7(303.3) | - |
| Simoni 2013 | USA | PC | AVF | - | 28 | 62.75 (13.4) | 38/34 |  |  |  |  |
|  |  |  | AVG | FA/UA | 44 |  |  |  |  |  |  |
| Staramos 2000 | Greece | RC | AVF | - | 68 | - | - | - | - | - | - |
|  |  |  | AVG | FA/UA/LL | 67 | - | - | - | - | - | - |
| Wang 2017 | USA | RC | AVF | - | 185 | 60.16 (14.8) | 116/89 | - | - | - | - |
|  |  |  | AVG | FA/UA | 24 | 63.96 (16.0) | 7/17 | - | - | - | - |

USA: United States of America; UK: United Kingdom; RCT: randomized controlled trial; AVF: arteriovenous fistula; AVG: arteriovenous graft; BaAVF: basilic vein AVF; BBAVF: brachiobasilic AVF; BrAVF: brachial vein AVF; RCAVF: radiocephalic AVF; UA: upper-arm; FA: forearm; LL: lower limb; tAVF: transposed AVF: nAVF: non-transposed AVF; M/F: male/female; SD: standard deviation; m: mean.

**Supplementary Table 3.** A summary of the medical conditions of patients receiving either AVF or AVG during hemodialysis

| *Study ID* | *Arm* | *Hypertension* | *Diabetes* | *CAD* | *PAD* | *PVD* | *Stroke/ TIA* | *CHF* | *Cancer* | *Current smoking* |
| --- | --- | --- | --- | --- | --- | --- | --- | --- | --- | --- |
| Coburn 1994 | BaAVF | - | 28 (48) | 29 (49) | - | 26 (46) | - | - | - | - |
|  | AVG | - | 20 (44) | 20 (44) | - | 13 (27) | - | - | - | - |
| Matsuura1998 | BaAVF | 20 (66) | 19 (64) | 13 (43) | - | 7 (23) | - | - | - | 10 (33) |
|  | AVG | 48 (70) | 32 (47) | 37 (54) | - | 22 (32) | - | - | - | 25 (37) |
| Ascher 1999 | nAVF | 150(61) | 135(55) | 98(40) | - | - | - | - | - | - |
|  | AVG |  |  |  | - | - | - | - | - | - |
| Kalman 1999 | AVF | - | 154(40) | - | - | - | - | - | - | - |
|  | AVG | - |  | - | - | - | - | - | - | - |
| Gibson 2001 | nAVF | - | 1211 (53.9) | - | - | - | - | - | - | 306 (13.6) |
|  | AVG | - |  | - | - | - | - | - | - |  |
| OLIVER 2001 | BBAVF | - | 28(48) | 19(32.2) | - | - | - | - | - | 19(32.2) |
|  | BCAVF | - | 24(43) | 20(25) | - | - | - | - | - | 13(16.3) |
|  | AVG | - | 55(69) | 14(25) | - | - | - | - | - | 20(35.7) |
| Dhingra 2001 | AVF | 360(26.9) | 358(26.7) | 496(37) | - | 300(22.4) | - | 520(38.8) | 109(8.1) | 537(40.1) |
|  | AVG | 951(30.4) | 1023(32.7) | 1458(46.4) | - | 932(29.8) | - | 1374(43.9) | 316(10.1) | 1267(40.5) |
| Lee 2004 | BBAVF | 3(15) | 31 (62) | 0(0.00) | - | - | - | - | - | - |
|  | AVG | 27 (54) | 2(10) | 1 (2) | - | - | - | - | - | - |
| Fitzgerald 2005 | nAVF | 21 (24) | 53 (62) | - | - | 23 (27) | - | - | - | 22 (26) |
|  | AVG | 12 (20) | 37 (62) | - | - | 9 (15) | - | - | - | 16 (27) |
| Kawecka 2005 | BBAVF | 439(61) | 66(9) | - | - | - | - | - | - | - |
|  | BCAVF |  |  | - | - | - | - | - | - | - |
|  | RCAVF |  |  | - | - | - | - | - | - | - |
|  | AVG |  |  |  |  |  |  |  |  |  |
| Keuter 2007 | BBAVF | 21 (40) | 20(38) | 21 (40) | 12(23) | - | 3(6) | - | - | - |
|  | AVG | 27 (51) | 25(47) | 12 (23) | 14(26) | - | 9(16) | - | - | - |
| Kakkos 2007 | BBAVF | - | - | - | - | - | - | - | - | - |
|  | AVG | - | - | - | - | - | - | - | - | - |
| Lee 2007 | AVF | - | 32 (54) | - | - | 17 (30) | - | - | - | - |
|  | AVG | - | 30 (59) | - | - | 11 (22) | - | - | - | - |
| Woo 2007 | tAVF | - | 97 (51) | - | - | - | - | - | - | - |
|  | AVG | - | 74 (45) | - | - | - | - | - | - | - |
| Weale 2007 | BBAVF | - | 33 (46.5) | - | - | - | - | - | - | - |
|  | AVG | - | 50 (43.9) | - | - | - | - | - | - | - |
| Chemla 2008 | BaAVF | - | 9(26) | - | - | - | - | - | - | - |
|  | AVG | - | 24(56) | - | - | - | - | - | - | - |
| MILBURN 2008 | BBAVF | - | 21 (40) | - | - | - | - | - | - | - |
|  | AVG | - | 15 (39) | - | - | - | - | - | - | - |
| Pflederer 2008 | Transposed | - | 73(45) | - | - | - | - | - | - | - |
|  | Non-transposed | - | 138(43) | - | - | - | - | - | - | - |
|  | RCAVF | - | 52(44) | - | - | - | - | - | - | - |
|  | BCAVF | - | 86(42) | - | - | - | - | - | - | - |
|  | AVG | - | 120(42) | - | - | - | - | - | - | - |
| Snyder 2008 | AVG | - | 32(60.7) | 25(46.4) | - | - | - | - | - | 22(41) |
|  | AVF | - | 14(61.7) | 7(31.9) | - | - | - | - | - | 7(31.9) |
| TORINA 2008 | BCAVF | 3 (23%) | 8 (62%) | - | - | - | - | - | - | - |
|  | BVAVF | 7 (17%) | 25 (60%) | - | - | - | - | - | - | - |
|  | AVG | 21 (22%) | 53 (56%) | - | - | - | - | - | - | - |
| Maya 2009 | BBAVF | 55 (82%) | 39 (58%) | 22 (33%) | 8 (12%) | - | 9 (13%) | - | - | - |
|  | BCAVF | 299 (93%) | 172 (53%) | 95 (30%) | 51 (16%) | - | 30 (9%) | - | - | - |
|  | AVG | 259 (90%) | 143 (49%) | 80 (28%) | 55 (19%) | - | 31 (11%) | - | - | - |
| Basel 2011 | BaAVF | 65(66.3) | 60(61.2) | 42(42.8) | - | 22(22.4) | - | - | - | 48(48.97) |
|  | PBG | 37(75.5) | 35(71.4) | 27(55.1) | - | 15(30.6) | - | - | - | 27(55.1) |
| Lioupis 2011 | BBAVF | 35(78) | 18(40) | 26(58) | - | 5(11) | - | - | - | - |
|  | BrAVF | 11(73) | 9(60) | 6(40) | - | 6(40) | - | - | - | - |
|  | AVG | 38(79) | 19(40) | 16(33) | - | 8(17) | - | - | - | - |
| Kim 2011 | AVF | 63 (68.5%) | 42 (45.7%) | 7 (7.6%) | 4 (4.4%) | - | 13 (14.1%) | 5 (5.4%) | - | - |
|  | AVG | 16 (43.2%) | 31 (83.8%) | 8 (21.6%) | 1 (2.7%) | - | 8 (21.6%) | 2 (5.4%) | - | - |
| Morosetti 2011 | AVG | - | 2 (7.4) | - | - | - | - | - | - | - |
|  | AVF | - | 8 (26.6) | - | - | - | - | - | - | - |
| Yan 2013 | tAVF | - | - | - | - | - | - | - | - | - |
|  | NAVF | - | - | - | - | - | - | - | - | - |
|  | AVG | - | - | - | - | - | - | - | - | - |
| Davoudi 2013 | BaAVF | - | - | - | - | - | - | - | - | - |
|  | AVG | - | - | - | - | - | - | - | - | - |
| Cui 2015 | AVF | 129 (93.5) | 65 (47.1) | 77 (55.8) | - | - | - | - | - | - |
|  | AVG | 41 (93.2) | 25 (56.8) | 23 (52.3) | - | - | - | - | - | - |
| Pham 2015 | BVAVF | 26 (90) | 21 (72) | - | - | - | - | - | - | - |
|  | AVG | 28 (88) | 23 (63) | - | - | - | - | - | - | - |
| Jadlowiec 2015 | AVF | 140 (94.0) | 85 (57.0) | 109 (73.2) | - | - | - | - | - | 68 (45.6) |
|  | AVG | 33 (89.2) | 22 (59.5) | 28 (75.7) | - | - | - | - | - | 19 (51.4) |
| Marques 2015 | BBAVF | 112(82) | 46(34) | 44(32) | - | 23(17) | 12(9) | - | - | 62(46) |
|  | AVG | 95(93) | 45(44) | 333(32) | - | 19(19) | 8(8) | - | - | 48(48) |
| Yuo 2015 | AVF | 8880(28.2) | 14455(45.9) | 8787(27.9) | - | 5858(18.6) | 3685(11.7) | 12975(41.2) | 2614(8.3) | 1921(6.1) |
|  | AVG | 3137(29.9) | 4983(47.5) | 2885(27.5) | - | 1972(18.8) | 1374(13.1) | 4417(42.1) | 871(8.3) | 535(5.1) |
| Chue 2016 | BBAVF | 71 (87.7%) | 50 (61.7%) | 21 (25.9%) | - | - | - | - | - | - |
|  | AVG | 36 (87.8%) | 23 (56.1%) | 13 (31.7%) | - | - | - | - | - | - |
| Harms 2016 | AVF | 560 (93) | 307 (51) | 184 (31) | - | 106 (18) | 120 (20) | 188 (31) | - | - |
|  | AVG |  |  |  | - |  |  |  | - | - |
| Park 2016 | AVF | - | 433 (58.0) | 103 (13.8) | - | 64 (8.5) | - | - | - | - |
|  | AVG | - | 143 (71.9) | 30 (15.1) | - | 23 (11.6) | - | - | - | - |
| Buggs 2017 | AVF | - | - | - | - | - | - | - | - | - |
|  | AVG | - | - | - | - | - | - | - | - | - |
| Dumaine 2017 | BBAVF | - | 46 (52.9%) | 51 (58.6%) | - | 9 (10.3%) | - | - | - | - |
|  | BCAVF | - | 98 (56.6%) | 95 (54.9%) | - | 28 (16.2%) | - | - | - | - |
|  | RCAVF | - | 35 (50.7%) | 30 (43.5%) | - | 7 (10.1%) | - | - | - | - |
|  | SBF (snuffbox) | - | 29 (52.7%) | 24 (43.6%) | - | 8 (14.5%) | - | - | - | - |
|  | AVG | - | 228 (54.4%) | 224 (53.5%) | - | 57 (13.6%) | - | - | - | - |
| Akoh 2018 | AVF | - | 8 (25%) | - | - | - | - | - | - | - |
|  | AVG | - | 6 (19.4%) | - | - | - | - | - | - | - |
| Arhuidese 2018 | AVF | 88.7 | 52.1 | 21 | 12.5 | - | 8.4 | 25.3 | 6.7 | 5.9 |
|  | AVG | 87.4 | 55.7 | 21 | 14.6 | - | 11.3 | 29.5 | 6.9 | 5.7 |
| Drouven 2018 | BaAVF | 44 (80) | 15 (27.3) | - | - | - | - | - | - | - |
|  | AVG | 66 (88) | 35 (46.7) | - | - | - | - | - | - | - |
| Arhuidese 2019 | AVF | 17,051 (88.9) | 8240 (43.0) | 5652 (29.5) | 3002 (15.7) | - | 1832 (9.6) | 6199 (32.3) | 2242 (11.7) | 496 (2.6) |
|  | AVG | 4226 (87.3) | 2324 (48.0) | 1356 (28.0) | 832 (17.2) | - | 561 (11.6) | 1784 (36.9) | 519 (10.7) | 108 (2.2) |
| Hicks 2019 | AVF | - | - | - | - | - | - | - | - | - |
|  | AVG | - | - | - | - | - | - | - | - | - |
| Itoga 2019 | AVF | 230 (82) | - | 48 (17) | - | - | - | - | - | 23 (8) |
|  | AVG | 37 (97) | - | 11 (29) | - | - | - | - | - | 3 (8) |
| Cheng 2020 | BBAVF | 15 (75.0) | 13 (65.0) | 7 (35.0) | - | - | 5 (25.0) | - | - | - |
|  | AVG | 96 (80.7) | 86 (72.3) | 49 (42.2) | - | - | 27 (22.7) | - | - | - |
| Tayebi 2020 | BBAVF | 15(50) | 13(43.3) | - | - | - | - | 11(36.7) | - | - |
|  | AVG | 12(40) | 13(43.3) | - | - | - | - | 9(30) | - | - |
| Voorzaat 2020 | RcAVF | - | 205 (36.8%) | 153 (27.5%) | - | 105 (18.9%) | 83 (14.9%) | - | - | - |
|  | AVF | - | 150 (39.7%) | 105 (27.8%) | - | 76 (20.1%) | 58 (15.3%) | - | - | - |
|  | AVG | - | 66 (62.3%) | 32 (30.2%) | - | 21 (19.8%) | 15 (14.2%) | - | - | - |
| Srikuea 2024 | BBAVF | 24 (86) | 11 (39) | - | - | - | - | - | - | 10 (35) |
|  | AVG | 68 (92) | 41 (55) | - | - | - | - | - | - | 17 (23) |
| Allemang 2014 | AVF | 257 (95) | 82 (30) | 69 (26) | 35 (13) | - | - | - | - | 123 (45) |
|  | AVG | 148 (97) | 54 (35) | 46 (30) | 20 (13) | - | - | - | - | 47 (31) |
| Asif 2005 | AVF | 79 (49%) | - | - | - | - | - | - | - | - |
|  | AVG |  | - | - | - | - | - | - | - | - |
| Bacchini 2001 | AVF | - | - | - | - | - | - | - | - | - |
|  | AVG | - | - | - | - | - | - | - | - | - |
| Charlton-Ouw 2012 | BaAVF | 60 (95.2) | 30(47.6) | - | - | - | - | - | - | - |
|  | AVG (synthetic) | 21 (100) | 15(71.4) | - | - | - | - | - | - | - |
|  | Heparin-bonded AVG | 19(90.5) | 13(61.9) | - | - | - | - | - | - | - |
| Danese 2006 | AVF | 6255(28) | 8265(37) | - | - | - | - | - | - | - |
|  | AVG | 18985(28) | 29533(42) | - | - | - | - | - | - | - |
| Falk 2016 | AVF | 122 (92.4) | 90 (62.9) | 44 (30.8) | - | 17 (11.9) | 7 (4.9) | 39 (27.3) | - | - |
|  | AVG | 136 (95.1) | 91 (68.9) | 53 (40.2) | - | 14 (10.6) | 5 (3.8) | 37 (28.0) | - | - |
| Galal 2024 | AVF | 9(36) | 7(28) | 7(28) | 5(20) | - | - | - | - | 10(40) |
|  | AVF | 8(32) | 7(28) | 7(28) | 6(24) | - | - | - | - | 10(40) |
| Ghaffarian 2018 | AVF UA | - | 28 (54) | 15 (29) | - | - | - | 17 (33) | - | - |
|  | AVF FA | - | 8 (50) | 9 (56) | - | - | - | 6 (38) | - | - |
|  | AVG | - | 86 (63) | 81 (60) | - | - | - | 50 (37) | - | - |
| Jadlowiec 2015 | AVF | 10 (14.3) | 41 (58.6) | - | - | - | - | - | - | 25 (35.7) |
|  | AVG | 11 (15.7) | 32 (45.7) | - | - | - | - | - | - | 23 (32.9) |
| kherlakian 1986 | AVF | 69(69) | 23(23) | - | - | 19(19) | - | - | - | - |
|  | AVG | 79(79) | 27(27) | - | - | 42(42) | - | - | - | - |
| kim 2021 | BrAVF | 12 (63.2%) | 8 (42.1%) | 3 (15.8%) | - | - | - | - | - | - |
|  | AVG | 29 (54.7%) | 26 (49.1%) | 13 (24.5%) | - | - | - | - | - | - |
| ladenheim 2017 | AVF | 16 (89) | 10 (56) | - | - | - | - | - | - | - |
|  | AVG | 35 (88) | 28 (70) | - | - | - | - | - | - | - |
| Lok 2013 | AVF | 727 (71.8) | 390 (38.5) | 237 (23.4) | - | 79 (7.8) | 92 (9.0) | 123 (12.1) | - | - |
|  | AVG | 98 (76.6) | 73 (57.0) | 37 (28.9) | - | 11 (8.6) | 15 (11.7) | 25 (19.5) | - | - |
| Simoni 2013 | AVF | 25 (35) | 17 (24) | 18 (25) | 5 (7) | - | 9 (12) | - | - | - |
|  | AVG |  |  |  |  | - |  | - | - | - |
| Staramos 2000 | AVF | - | - | - | - | - | - | - | - | - |
|  | AVG | - | - | - | - | - | - | - | - | - |
| Wang 2017 | AVF | 184 (99) | 99 (54) | 94 (51) | - | - | - | - | - | - |
|  | AVG | 23 (96) | 18 (75) | 14 (58) | - | - | - | - | - | - |

Data are presented as numbers (percentage). AVF: arteriovenous fistula; AVG: arteriovenous graft; BaAVF: basilic vein AVF; BBAVF: brachiobasilic AVF; BrAVF: brachial vein AVF; RCAVF: radiocephalic AVF; UA: upper-arm; FA: forearm; LL: lower limb; tAVF: transposed AVF: nAVF: non-transposed AVF; CAD: coronary artery disease; PAD: peripheral artery disease; PVD: peripheral vascular disease; CHF: chronic heart failure.

**Supplementary Table 4.** A summary of the methodological quality of included observational studies using the NIH tool

| Study | Q1 | Q2 | Q3 | Q4 | Q5 | Q6 | Q7 | Q8 | Q9 | Q10 | Q11 | Q12 | Q13 | Q14 | Total score | Quality |
| --- | --- | --- | --- | --- | --- | --- | --- | --- | --- | --- | --- | --- | --- | --- | --- | --- |
| Coburn 1994 | 1 | 1 | 2 | 1 | 0 | 1 | 2 | 2 | 2 | 2 | 2 | 1 | 2 | 0 | 19 | Fair |
| Matsuura1998 | 1 | 1 | 2 | 2 | 0 | 1 | 2 | 2 | 2 | 2 | 2 | 1 | 2 | 0 | 20 | Fair |
| Ascher 1999 | 2 | 1 | 2 | 1 | 0 | 1 | 2 | 2 | 2 | 2 | 2 | 1 | 2 | 0 | 20 | Fair |
| Kalman 1999 | 2 | 1 | 2 | 1 | 0 | 1 | 2 | 2 | 2 | 2 | 2 | 1 | 2 | 2 | 22 | Good |
| Gibson 2001 | 1 | 1 | 2 | 2 | 0 | 1 | 2 | 2 | 2 | 2 | 2 | 1 | 2 | 2 | 22 | Good |
| OLIVER 2001 | 1 | 1 | 1 | 2 | 0 | 1 | 2 | 2 | 2 | 1 | 2 | 1 | 2 | 0 | 18 | Fair |
| YOUNG 2001 | 1 | 1 | 2 | 2 | 0 | 1 | 2 | 1 | 2 | 1 | 2 | 1 | 2 | 0 | 18 | Fair |
| Lee 2004 | 1 | 1 | 2 | 2 | 0 | 1 | 2 | 2 | 2 | 1 | 2 | 1 | 2 | 0 | 19 | Fair |
| Fitzgerald 2005 | 2 | 1 | 2 | 2 | 2 | 1 | 2 | 2 | 2 | 2 | 2 | 1 | 2 | 0 | 23 | Good |
| Kawecka 2005 | 1 | 1 | 2 | 1 | 0 | 1 | 2 | 2 | 2 | 2 | 2 | 1 | 2 | 0 | 19 | Fair |
| Kakkos 2007 | 2 | 1 | 2 | 1 | 0 | 2 | 2 | 2 | 2 | 2 | 2 | 1 | 2 | 0 | 21 | Good |
| Lee 2007 | 1 | 1 | 2 | 2 | 0 | 1 | 2 | 2 | 2 | 1 | 2 | 1 | 2 | 0 | 19 | Fair |
| Woo 2007 | 1 | 1 | 2 | 1 | 0 | 1 | 2 | 2 | 2 | 2 | 2 | 1 | 2 | 0 | 19 | Fair |
| Weale 2007 | 1 | 1 | 2 | 1 | 0 | 1 | 2 | 2 | 2 | 2 | 2 | 1 | 2 | 2 | 21 | Good |
| Chemla 2008 | 1 | 1 | 2 | 2 | 0 | 2 | 2 | 1 | 2 | 2 | 2 | 1 | 2 | 0 | 20 | Fair |
| MILBURN 2008 | 2 | 1 | 2 | 1 | 0 | 2 | 2 | 1 | 2 | 2 | 2 | 1 | 2 | 0 | 20 | Fair |
| Pflederer 2008 | 2 | 1 | 2 | 1 | 0 | 1 | 2 | 2 | 2 | 2 | 2 | 1 | 2 | 0 | 20 | Fair |
| Snyder 2008 | 1 | 1 | 2 | 1 | 0 | 1 | 2 | 2 | 2 | 2 | 2 | 1 | 2 | 0 | 19 | Fair |
| TORINA 2008 | 1 | 1 | 2 | 1 | 0 | 1 | 2 | 1 | 2 | 1 | 2 | 1 | 2 | 0 | 17 | Fair |
| Maya 2009 | 2 | 1 | 2 | 2 | 0 | 1 | 2 | 2 | 2 | 1 | 2 | 1 | 2 | 0 | 20 | Fair |
| Basel 2011 | 2 | 1 | 2 | 1 | 0 | 1 | 2 | 2 | 2 | 2 | 2 | 0 | 2 | 0 | 19 | Fair |
| Lioupis 2011 | 2 | 1 | 2 | 1 | 0 | 2 | 2 | 2 | 2 | 2 | 2 | 1 | 1 | 0 | 20 | Fair |
| Kim 2011 | 1 | 1 | 2 | 1 | 0 | 1 | 2 | 2 | 2 | 2 | 2 | 1 | 2 | 0 | 19 | Fair |
| Clark 2013 | 2 | 1 | 2 | 1 | 0 | 1 | 2 | 2 | 2 | 2 | 2 | 1 | 2 | 0 | 20 | Fair |
| Cui 2015 | 2 | 1 | 1 | 1 | 0 | 1 | 2 | 2 | 2 | 1 | 2 | 1 | 1 | 0 | 17 | Fair |
| D. Pham 2015 | 2 | 1 | 1 | 2 | 0 | 1 | 2 | 2 | 2 | 2 | 2 | 1 | 1 | 0 | 19 | Fair |
| Jadlowiec 2015 | 1 | 1 | 1 | 2 | 0 | 1 | 2 | 2 | 2 | 2 | 2 | 1 | 1 | 2 | 20 | Fair |
| Marques 2015 | 1 | 1 | 2 | 1 | 0 | 1 | 2 | 1 | 2 | 2 | 2 | 1 | 2 | 0 | 18 | Fair |
| Yuo 2015 | 1 | 1 | 1 | 1 | 0 | 1 | 2 | 2 | 2 | 1 | 2 | 1 | 1 | 0 | 16 | Fair |
| Chue 2016 | 1 | 1 | 2 | 2 | 0 | 1 | 2 | 1 | 2 | 1 | 2 | 1 | 1 | 0 | 17 | Fair |
| Harms 2016 | 1 | 1 | 0 | 0 | 0 | 1 | 2 | 1 | 2 | 1 | 2 | 1 | 0 | 0 | 12 | Fair |
| Park 2016 | 2 | 1 | 2 | 2 | 0 | 2 | 2 | 2 | 2 | 1 | 2 | 1 | 2 | 2 | 23 | Good |
| Buggs 2017 | 2 | 1 | 2 | 1 | 0 | 1 | 2 | 1 | 2 | 1 | 2 | 1 | 1 | 0 | 17 | Fair |
| Dumaine 2017 | 2 | 1 | 2 | 2 | 0 | 1 | 2 | 2 | 2 | 2 | 2 | 1 | 2 | 0 | 21 | Good |
| Akoh 2018 | 1 | 1 | 1 | 2 | 0 | 1 | 2 | 2 | 2 | 1 | 2 | 1 | 1 | 0 | 17 | Fair |
| Arhuidese 2018 | 1 | 1 | 2 | 1 | 0 | 1 | 2 | 2 | 2 | 2 | 2 | 1 | 2 | 0 | 19 | Fair |
| Drouven 2018 | 1 | 1 | 0 | 1 | 0 | 1 | 2 | 2 | 2 | 2 | 2 | 1 | 2 | 0 | 17 | Fair |
| Arhuidese 2019 | 2 | 1 | 2 | 1 | 0 | 2 | 2 | 2 | 2 | 2 | 2 | 1 | 2 | 0 | 21 | Good |
| Hicks 2019 | 1 | 1 | 1 | 1 | 0 | 1 | 2 | 2 | 2 | 0 | 2 | 1 | 0 | 2 | 16 | Fair |
| Itoga 2019 | 2 | 1 | 2 | 2 | 0 | 1 | 2 | 2 | 2 | 2 | 2 | 1 | 2 | 0 | 21 | Good |
| Cheng 2020 | 1 | 1 | 2 | 2 | 0 | 2 | 2 | 2 | 2 | 2 | 2 | 1 | 1 | 0 | 20 | Fair |
| Voorzaat 2020 | 2 | 1 | 1 | 1 | 0 | 2 | 2 | 2 | 2 | 2 | 2 | 1 | 1 | 0 | 19 | Fair |
| Srikuea 2024 | 2 | 1 | 2 | 2 | 0 | 2 | 2 | 2 | 2 | 1 | 2 | 1 | 2 | 0 | 21 | Good |
| Allemang 2014 | 1 | 1 | 2 | 1 | 0 | 1 | 2 | 1 | 2 | 1 | 2 | 1 | 1 | 2 | 18 | Fair |
| Asif 2005 | 2 | 0 | 1 | 0 | 0 | 1 | 0 | 2 | 2 | 1 | 2 | 1 | 1 | 0 | 13 | Fair |
| Bacchini 2001 | 1 | 1 | 1 | 0 | 0 | 1 | 0 | 2 | 2 | 2 | 2 | 1 | 2 | 0 | 15 | Fair |
| Charlton-Ouw 2012 | 2 | 0 | 1 | 1 | 0 | 1 | 2 | 2 | 2 | 1 | 2 | 1 | 1 | 0 | 16 | Fair |
| Danese 2006 | 1 | 1 | 2 | 1 | 0 | 1 | 2 | 2 | 2 | 2 | 2 | 1 | 2 | 0 | 19 | Fair |
| Ghaffarian 2018 | 2 | 1 | 2 | 0 | 0 | 1 | 1 | 2 | 2 | 1 | 1 | 1 | 2 | 2 | 18 | Fair |
| Kherlakian 1986 | 0 | 0 | 2 | 0 | 0 | 1 | 2 | 1 | 2 | 2 | 2 | 1 | 2 | 0 | 15 | Fair |
| Kim 2021 | 1 | 1 | 2 | 1 | 0 | 1 | 1 | 1 | 2 | 1 | 2 | 1 | 2 | 2 | 18 | Fair |
| Ladenheim 2017 | 2 | 1 | 2 | 1 | 0 | 1 | 2 | 1 | 2 | 2 | 2 | 1 | 2 | 0 | 19 | Fair |
| Lok 2013 | 2 | 1 | 2 | 1 | 0 | 1 | 1 | 2 | 2 | 1 | 2 | 1 | 2 | 0 | 18 | Fair |
| Simoni 2013 | 2 | 1 | 2 | 1 | 0 | 2 | 2 | 2 | 2 | 1 | 2 | 1 | 2 | 0 | 20 | Fair |
| Staramos 2000 | 2 | 1 | 2 | 1 | 0 | 2 | 2 | 2 | 2 | 2 | 2 | 1 | 2 | 0 | 21 | Good |
| Jadlowiec 2015 | 2 | 1 | 2 | 1 | 0 | 2 | 2 | 2 | 2 | 2 | 2 | 1 | 2 | 0 | 21 | Good |
| Wang 2017 | 2 | 1 | 2 | 1 | 0 | 2 | 2 | 2 | 2 | 2 | 2 | 1 | 2 | 0 | 21 | Good |

Q1. Was the research question or objective in this paper clearly stated?
Q2. Was the study population clearly specified and defined?
Q3. Was the participation rate of eligible persons at least 50%?
Q4. Were all the subjects selected or recruited from the same or similar populations (including the same time period)? Were inclusion and exclusion criteria for being in the study prespecified and applied uniformly to all participants?
Q5. Was a sample size justification, power description, or variance and effect estimates provided?
Q6. For the analyses in this paper, were the exposure(s) of interest measured prior to the outcome(s) being measured?
Q7. Was the timeframe sufficient so that one could reasonably expect to see an association between exposure and outcome if it existed?
Q8. For exposures that can vary in amount or level, did the study examine different levels of the exposure as related to the outcome (e.g., categories of exposure, or exposure measured as continuous variable)?
Q9. Were the exposure measures (independent variables) clearly defined, valid, reliable, and implemented consistently across all study participants?
Q10. Was the exposure(s) assessed more than once over time?
Q11. Were the outcome measures (dependent variables) clearly defined, valid, reliable, and implemented consistently across all study participants?
Q12. Were the outcome assessors blinded to the exposure status of participants?
Q13. Was loss to follow-up after baseline 20% or less?
Q14. Were key potential confounding variables measured and adjusted statistically for their impact on the relationship between exposure(s) and outcome(s)?

**Supplementary Table 5.** A summary of complications reported with AVF vs. AVG use in hemodialysis patient stratified by complication type and AVF type

| Complication | Measures | BaAVF vs. AVG | BBAVF vs. AVG | BCAVF vs. AVG | BrAVF vs. AVG | RCAVF vs. AVG | UA-AVF vs. AVG | Not mentioned vs. AVG |
| --- | --- | --- | --- | --- | --- | --- | --- | --- |
| AV access malfunction | Studies (N) | 1 | - | - | 1 | - | - | - |
|  | OR (95% CI) | 6.83 (0.27, 171.21) | - | - | 7 (0.13, 367.64) | - | - | - |
|  | I^2^ (p) | NA | - | - | NA | - | - | - |
| Aneurysm | Studies (N) | 2 | 2 | - | 1 | - | - | 1 |
|  | OR (95% CI) | 0.41 (0.10, 1.68) | 1.11 (0.08, 15.910 | - | 0.42 (0.02, 8.58) | - | - | 0.41 (0.08, 2.08) |
|  | I^2^ (p) | 0 (0.89) | 49.35 (0.16) | - | NA | - | - | NA |
| Bleeding | Studies (N) | 3 | 2 | 1 | - | - | - | 1 |
|  | OR (95% CI) | 1.69 (0.55, 5.18) | 3.53 (0.86, 14.53) | 1.44 (0.09, 23.46) | - | - | - | 4.50 (0.54, 37.81) |
|  | I^2^ (p) | 0 (0.50) | 0 (0.81) | NA | - | - | - | NA |
| Hematoma | Studies (N) | 1 | 4 | - | 2 | - | - | - |
|  | OR (95% CI) | 7.15 (0.72, 70.92) | 3.33 (0.74, 14.98) | - | 13.93 (1.76, 110.36) | - | - | - |
|  | I^2^ (p) | NA | 9.64 (0.27) | - | 0 (0.55) | - | - | - |
| Infection | Studies (N) | 5 | 8 | 1 | 3 | 1 | 2 | 4 |
|  | OR (95% CI) | 0.19 (0.08, 0.45) | 0.18 (0.08, 0.43) | 0.13 (0.02, 1.02) | 0.37 (0.06, 2.13) | 0.14 (0.05, 0.37) | 0.35 (0.17, 0.74) | 0.66 (0.14, 3.12) |
|  | I^2^ (p) | 0 (0.69) | 0 (0.99) | NA | 0 (0.99) | NA | 0 (0.69) | 68.78 (0.02) |
| Limb edema | Studies (N) | 1 | 3 | 1 | 1 | - | - | - |
|  | OR (95% CI) | 0.24 (0.02, 2.74) | 0.96 (0.22, 4.11) | 0.61 (0.18, 2.08) | 7.23 (0.61, 86.15) | - | - | - |
|  | I^2^ (p) | NA | 47.02 (0.15) | NA | NA | - | - | - |
| Non-maturation | Studies (N) | - | 3 | 1 | 1 | - | 1 | 3 |
|  | OR (95% CI) | - | 2.37 (0.24, 23.55) | 20.72 (1.14, 375.82) | 14.59 (0.77, 276.62) | - | 3.58 (0.17, 75.91) | 29.98 (5.91, 152.09) |
|  | I^2^ (p) | - | 54.27 (0.11) | NA | NA | - | NA | 0 (0.48) |
| Pseudoaneurysm | Studies (N) | 2 | 1 | - | 1 | - | - | - |
|  | OR (95% CI) | 0.82 (0.24, 2.82) | 0.17 (0.01, 3.64) | - | 0.60 (0.03, 11.53) | - | - | - |
|  | I^2^ (p) | 0 (0.89) | NA | - | NA | - | - | - |
| Seroma | Studies (N) | 1 | 1 | 1 | 1 | - | - | - |
|  | OR (95% CI) | 0.24 (0.01, 4.50) | 0.12 (0.01, 2.13) | 0.12 (0.01, 2.24) | 0.74 (0.04, 14.62) | - | - | - |
|  | I^2^ (p) | NA | NA | NA | NA | - | - | - |
| Steal syndrome | Studies (N) | 4 | 5 | 1 | 3 | - | 1 | 3 |
|  | OR (95% CI) | 0.61 (0.27, 1.42) | 1.23 (0.46, 3.29) | 1.25 (0.40, 3.95) | 0.52 (0.10, 2.57) | - | 0.69 (0.13, 3.52) | 1.45 (0.75, 2.79) |
|  | I^2^ (p) | 0 (0.53) | 14.88 (0.21) | NA | 0 (0.99) | - | NA | 0 (0.56) |
| Stenosis | Studies (N) | 2 | 5 | - | 1 | - | 1 | 3 |
|  | OR (95% CI) | 2.16 (1.04, 4.49) | 0.58 (0.27, 1.24) | - | 1.55 (0.47, 5.17) | - | 0.91 (0.40, 2.10) | 1.82 (0.34, 9.83) |
|  | I^2^ (p) | 0 (0.57) | 69.05 (0.00) | - | NA | - | NA | 46.46 (0.17) |
| Thrombosis | Studies (N) | 4 | 8 | - | 3 | 1 | 2 | 4 |
|  | OR (95% CI) | 0.32 (0.15, 0.67) | 0.27 (0.09, 0.80) | - | 0.71 (0.33, 1.52) | 0.36 (0.22, 0.58) | 0.24 (0.16, 0.38) | 0.43 (0.13, 1.46) |
|  | I^2^ (p) | 55.92 (0.08) | 85.03 (0.00) | - | 0 (0.29) | NA | 0 (0.55) | 78.64 (0.00) |
| Vascular hypertension | Studies (N) | 1 | 3 | - | - | - | - | - |
|  | OR (95% CI) | 3.10 (0.12, 79.23) | 1.72 (0.25, 11.91) | - | - | - | - | - |
|  | I^2^ (p) | NA | 64.09 (0.06) | - | - | - | - | - |

- indicates no available data. AV: arteriovenous; AVF: arteriovenous fistula; AVG: arteriovenous graft; OR: odds ratio; CI: confidence interval; NA: Not applicable; BaAVF: basilic vein AVF; BBAVF: brachiobasilic AVF; BrAVF: brachial vein AVF; RCAVF: radiocephalic AVF; UA-AVF: upper-arm AVF.

**
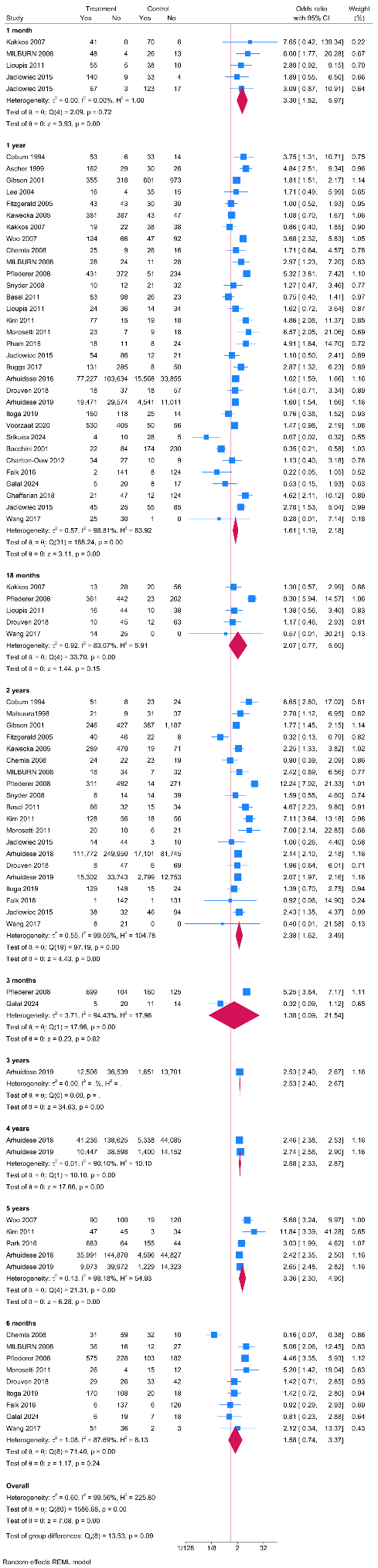
**

**Figure S1. Primary patency by time.**

**
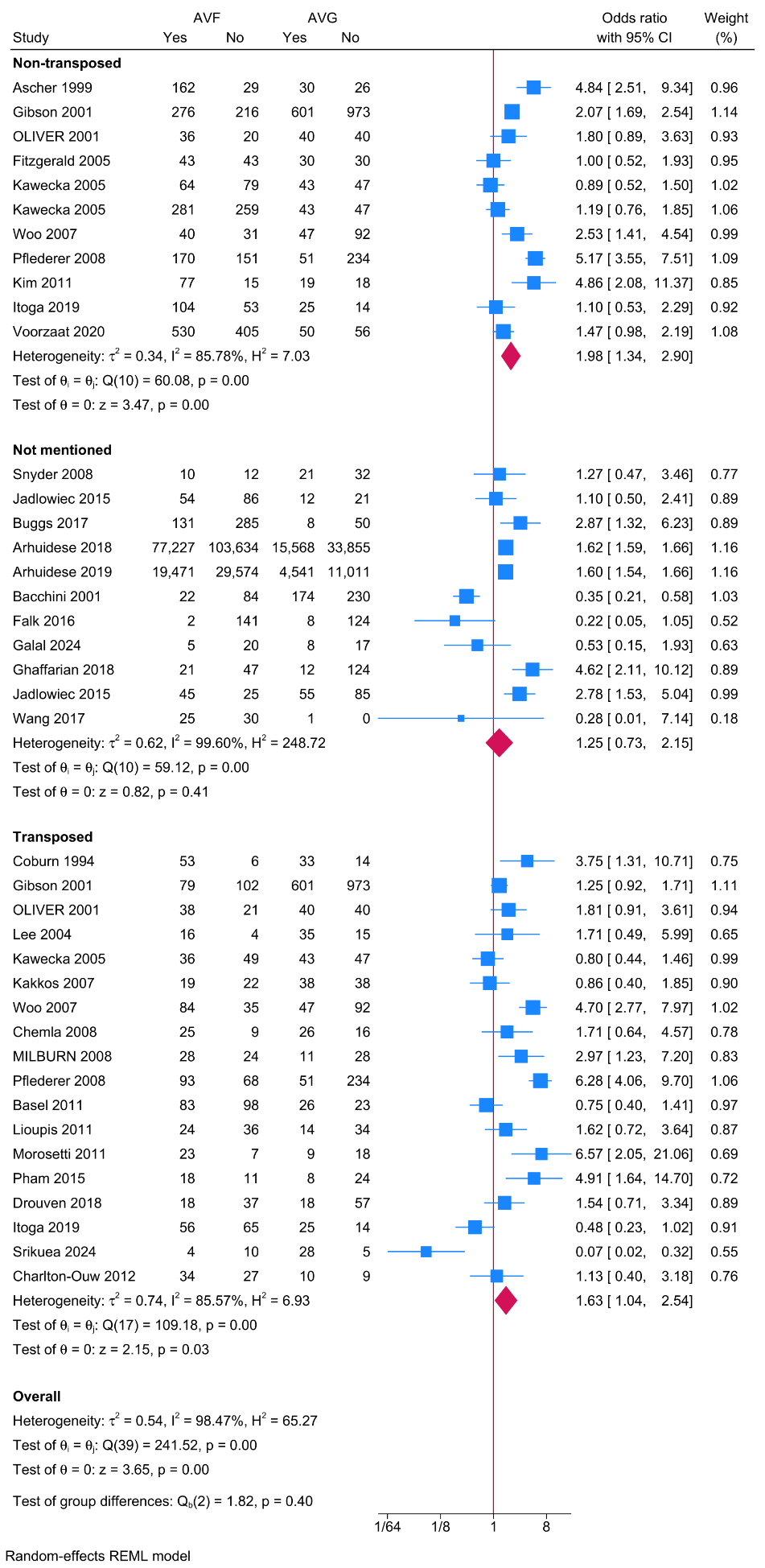
**

**Figure S2. Primary patency by AVF transposition status (1 year).**

**
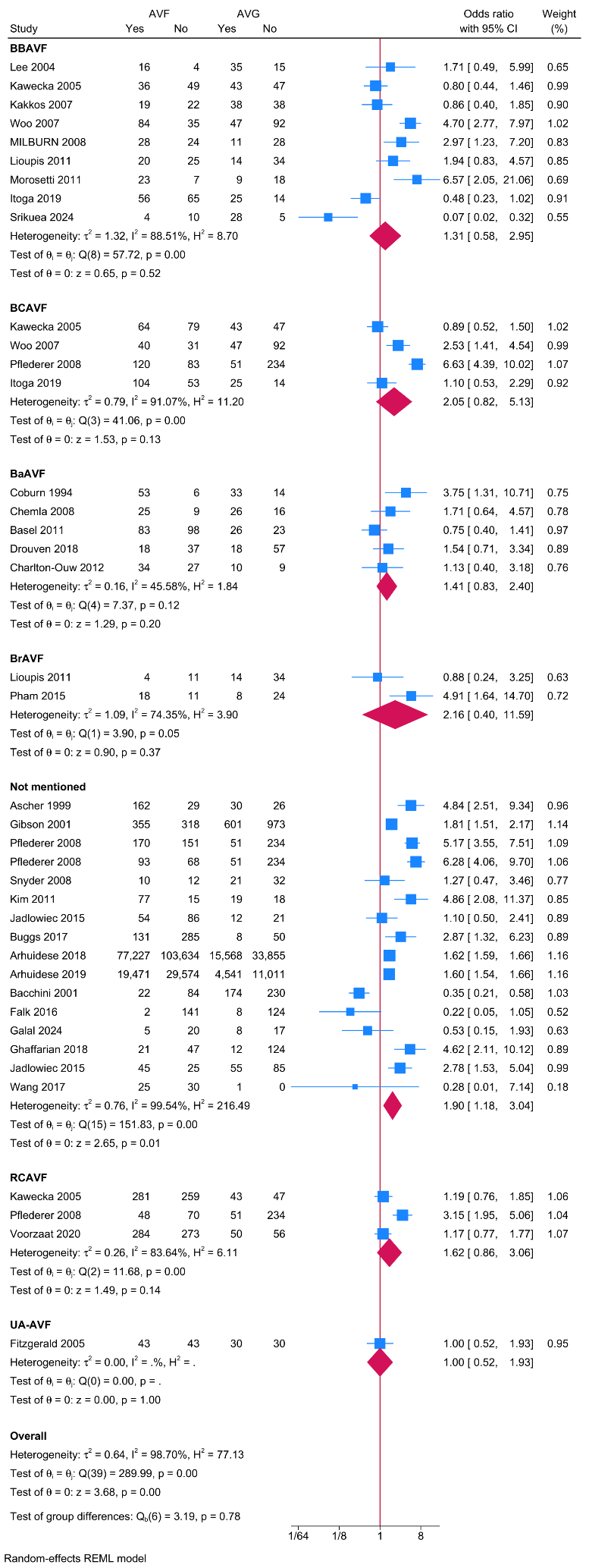
**

**Figure S3. Primary patency by AVF type (1 year).**

**
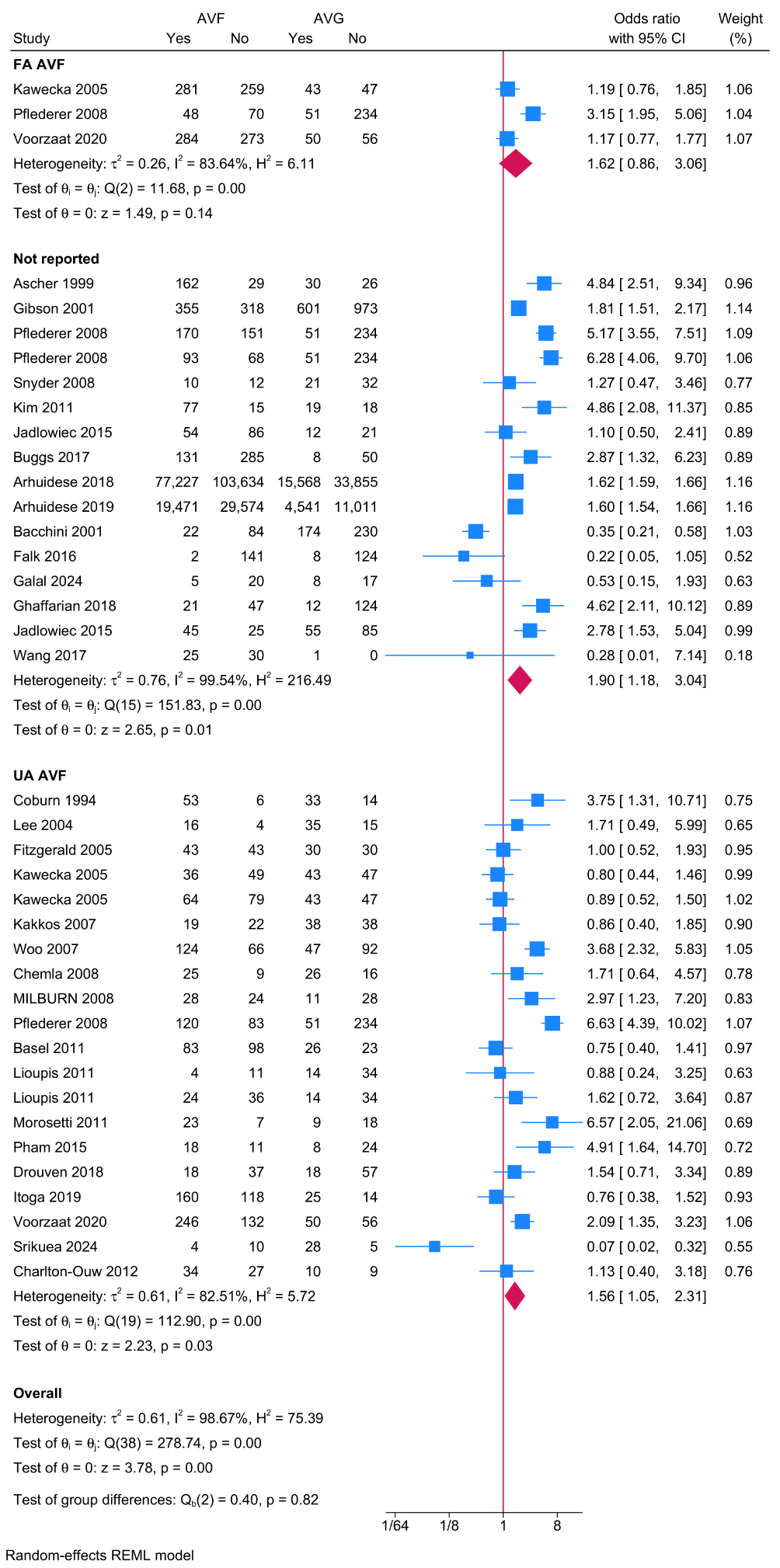
**

**Figure S4. Primary patency by AVF site (1 year).**

**
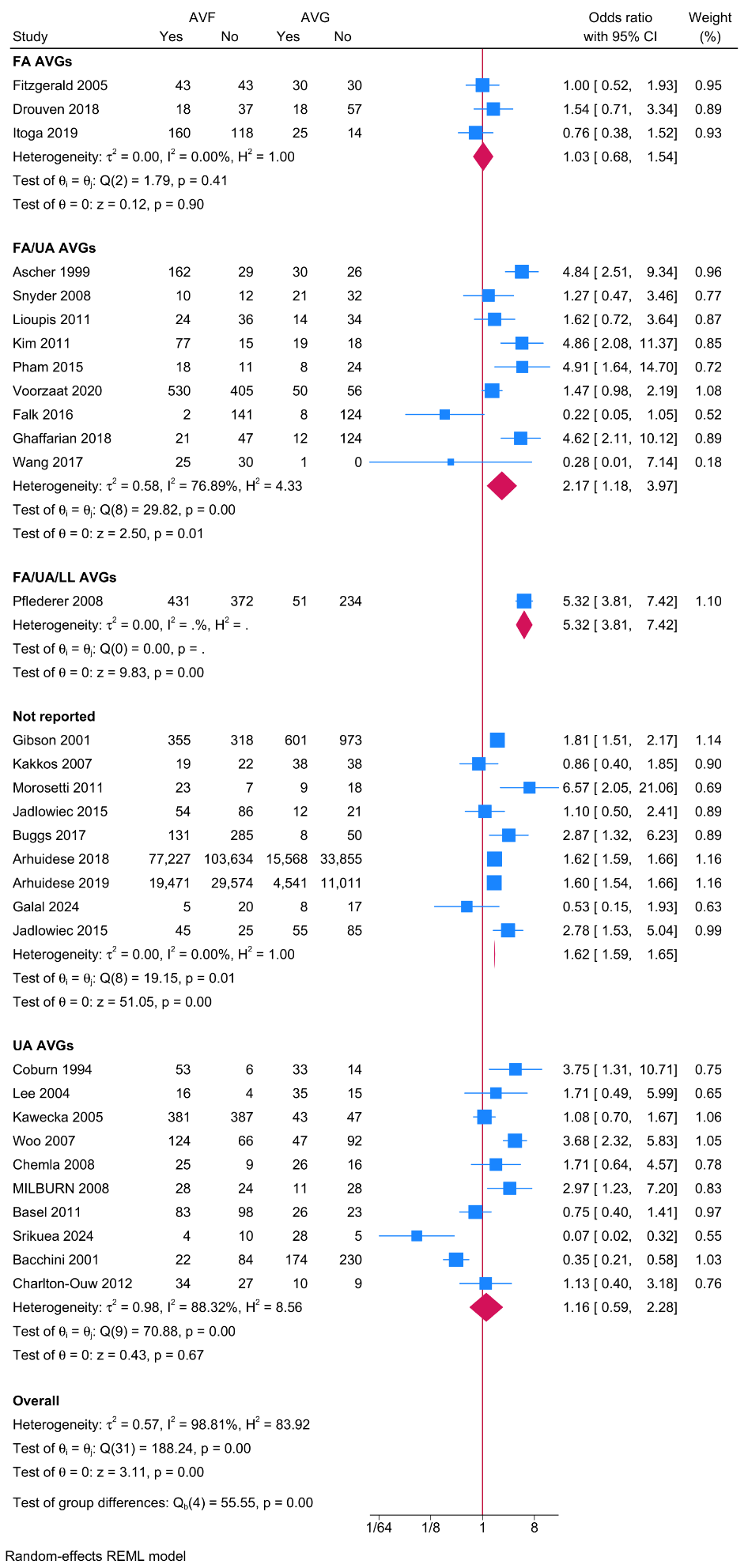
**

**Figure S5. Primary patency by AVG site (1 year).**

**
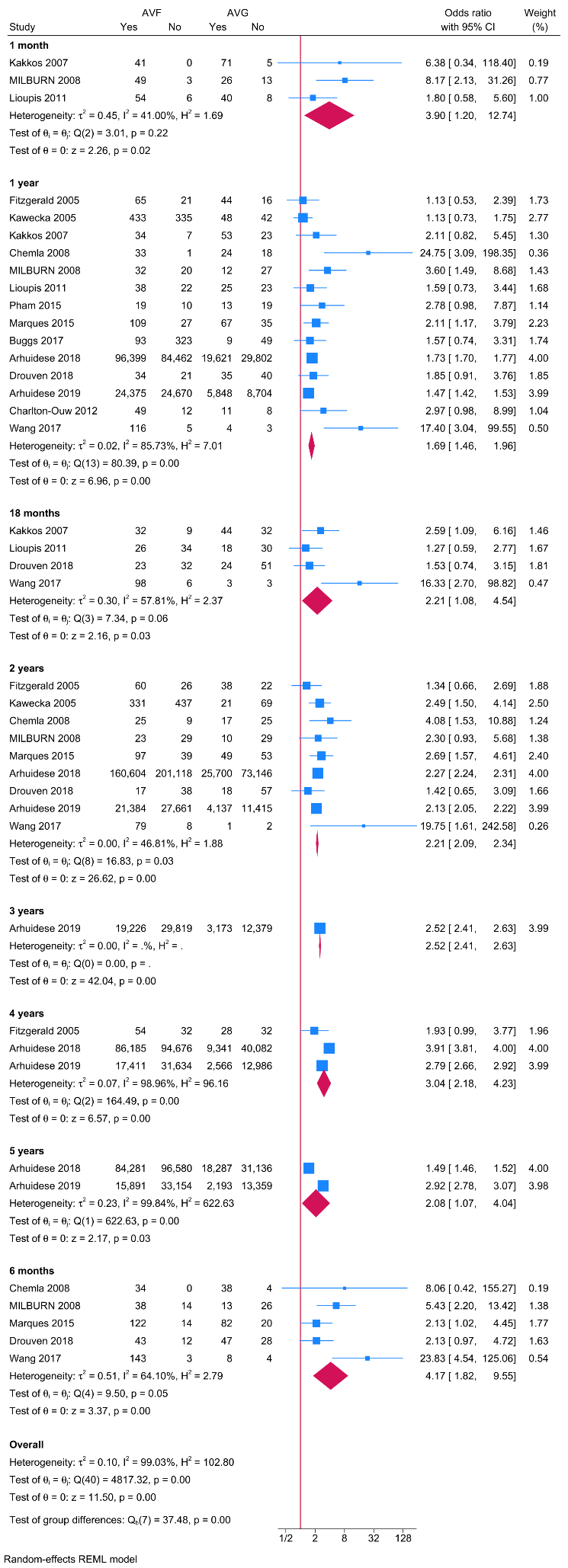
**

**Figure S6. Primary-assisted patency by time.**

**
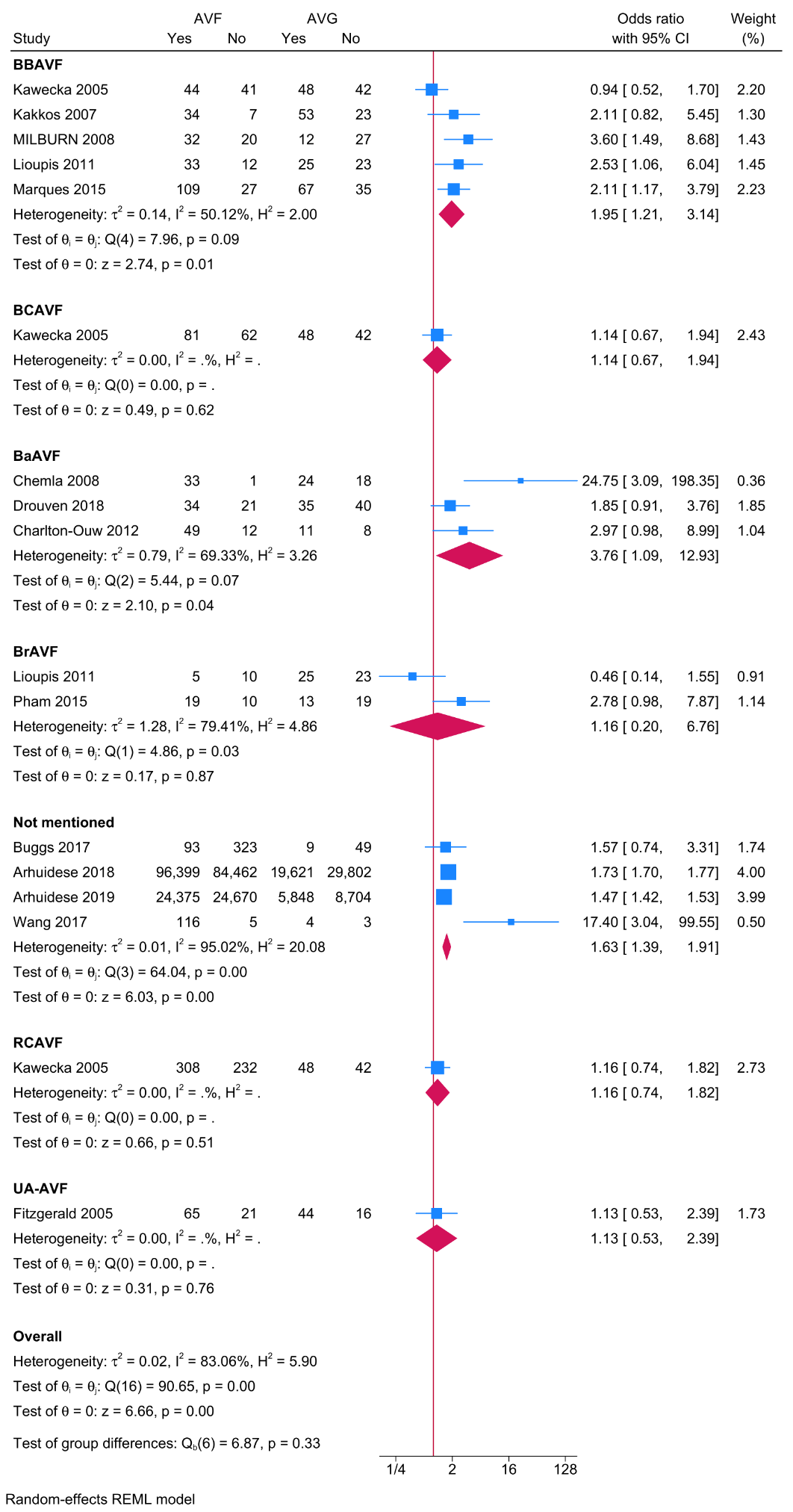
**

**Figure S7. Primary-assisted patency by AVF type (1 year).**

**
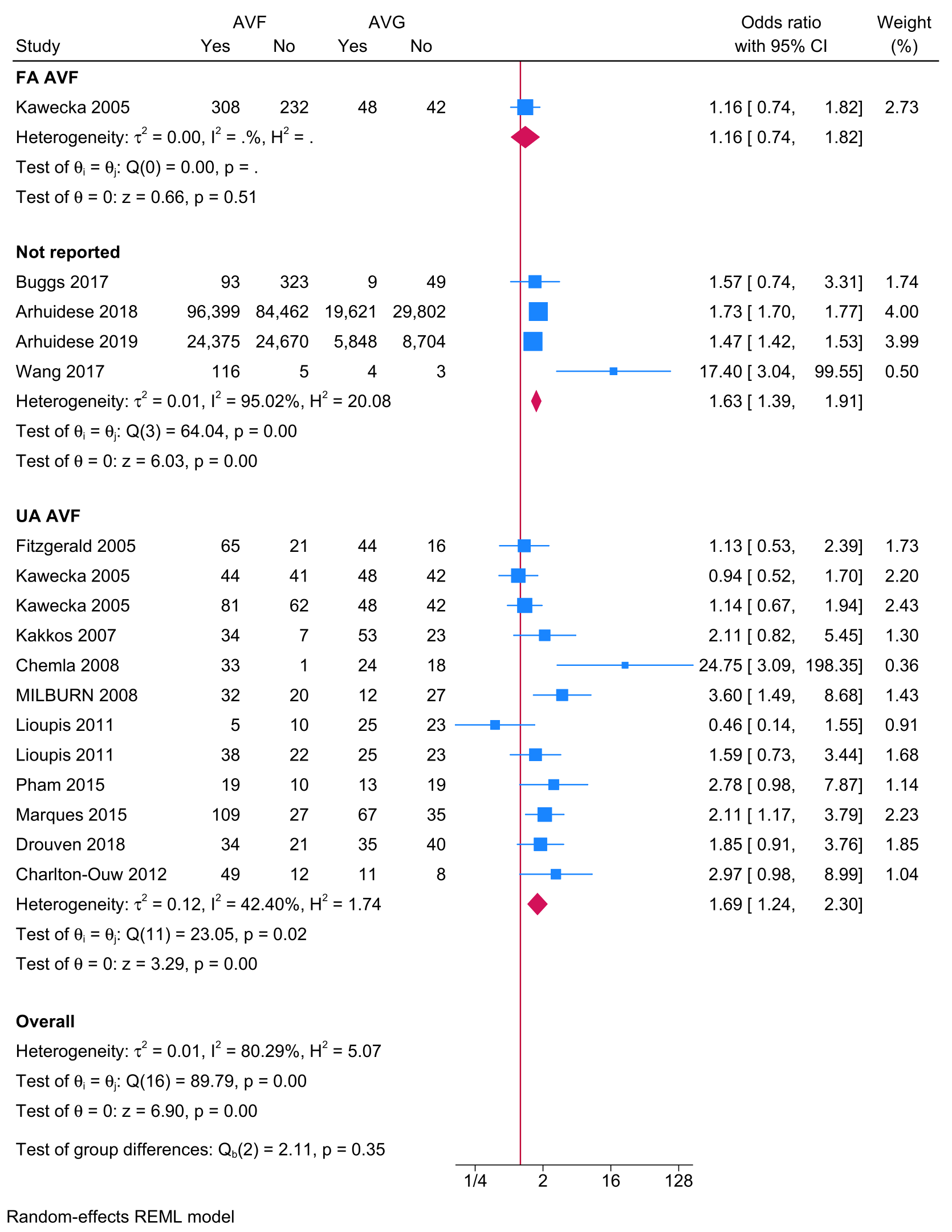
**

**Figure S8. Primary-assisted patency by AVF site (1 year).**

**
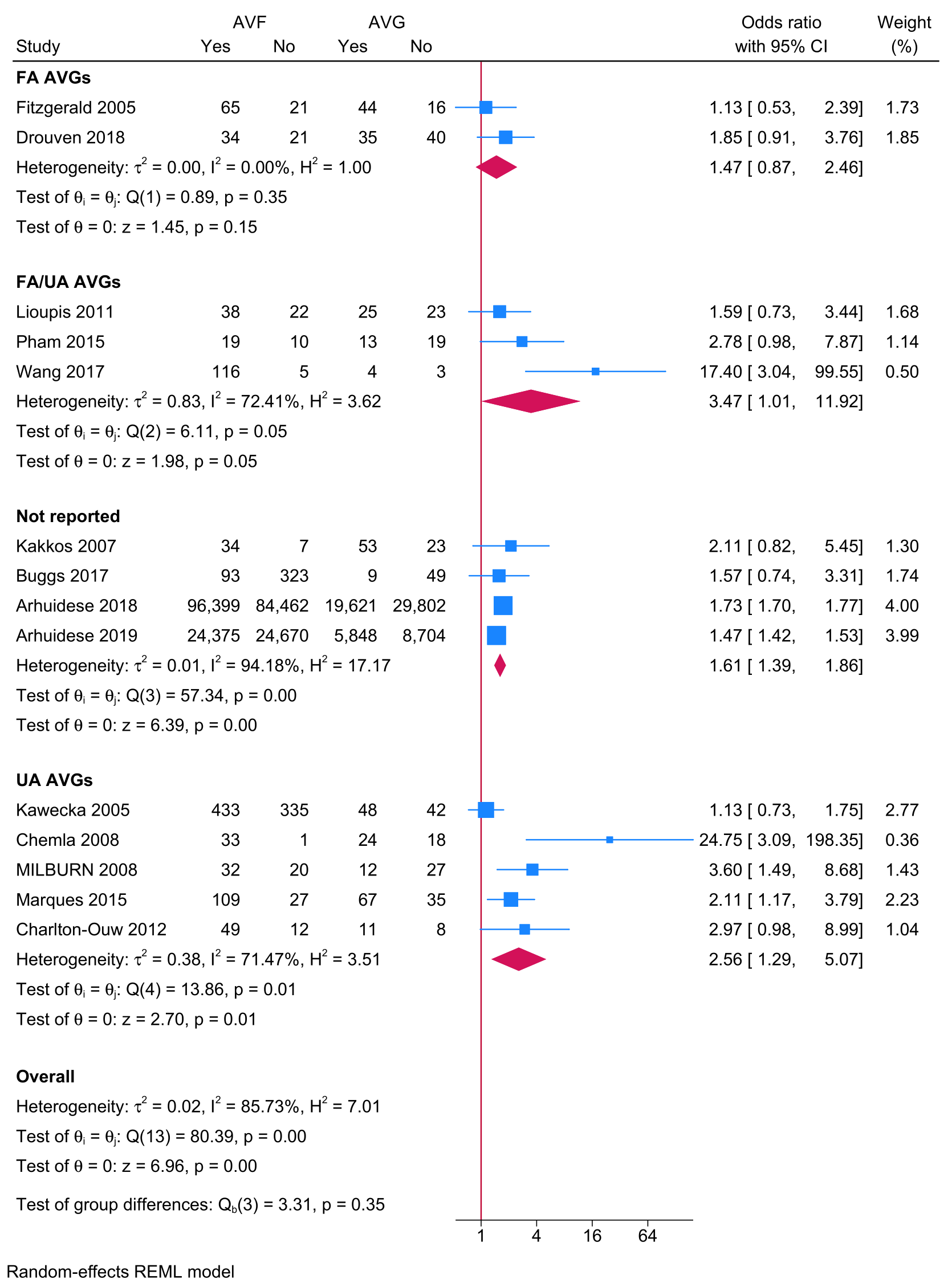
**

**Figure S9. Primary-assisted patency by AVG site (1 year).**

**
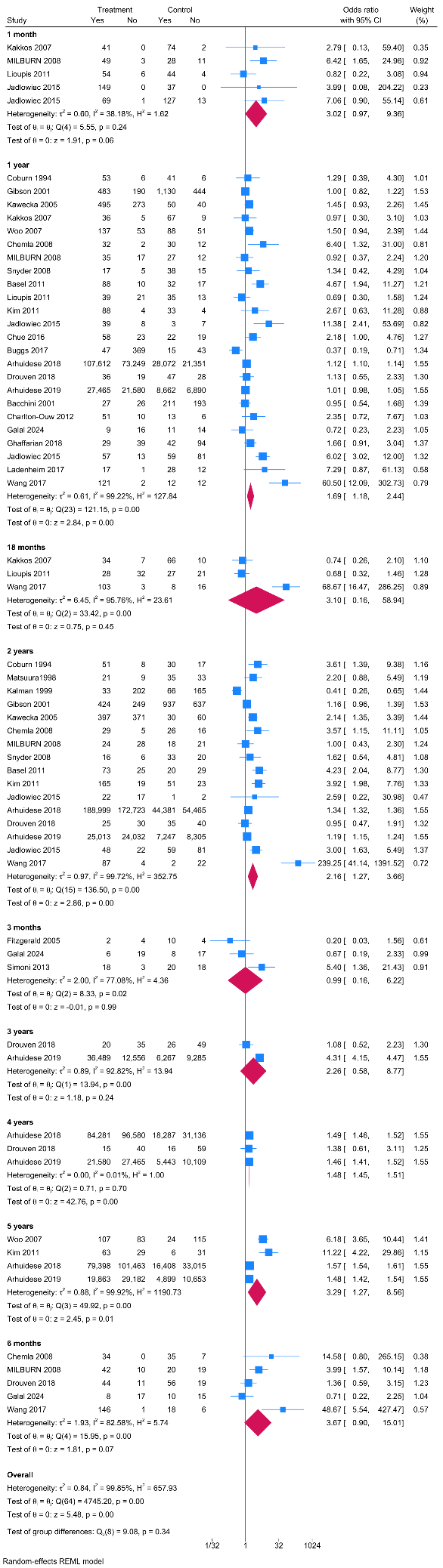
**

**Figure S10. Secondary patency by time.**

**
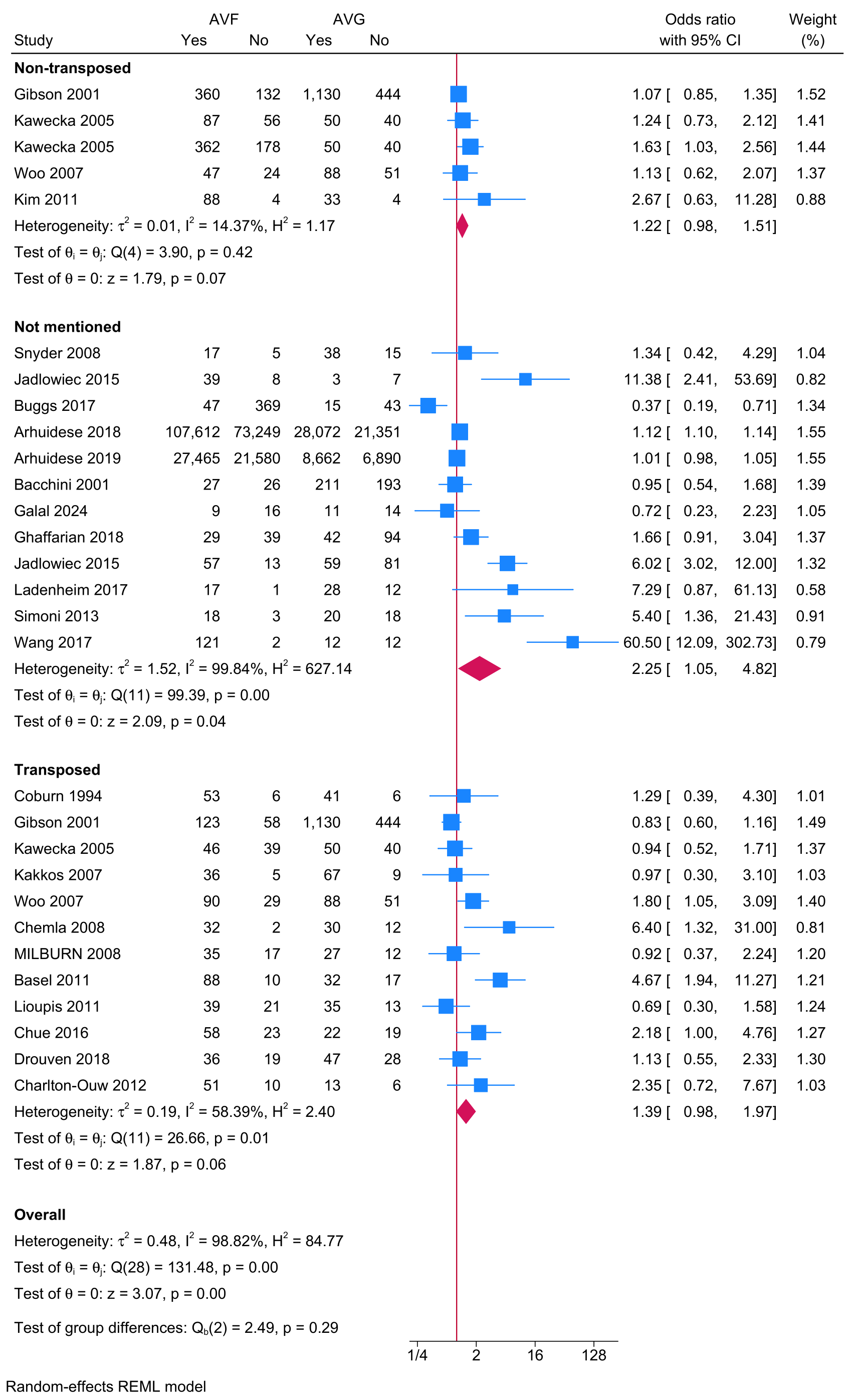
**

**Figure S11. Secondary patency by AVF transposition status (1 year).**

**
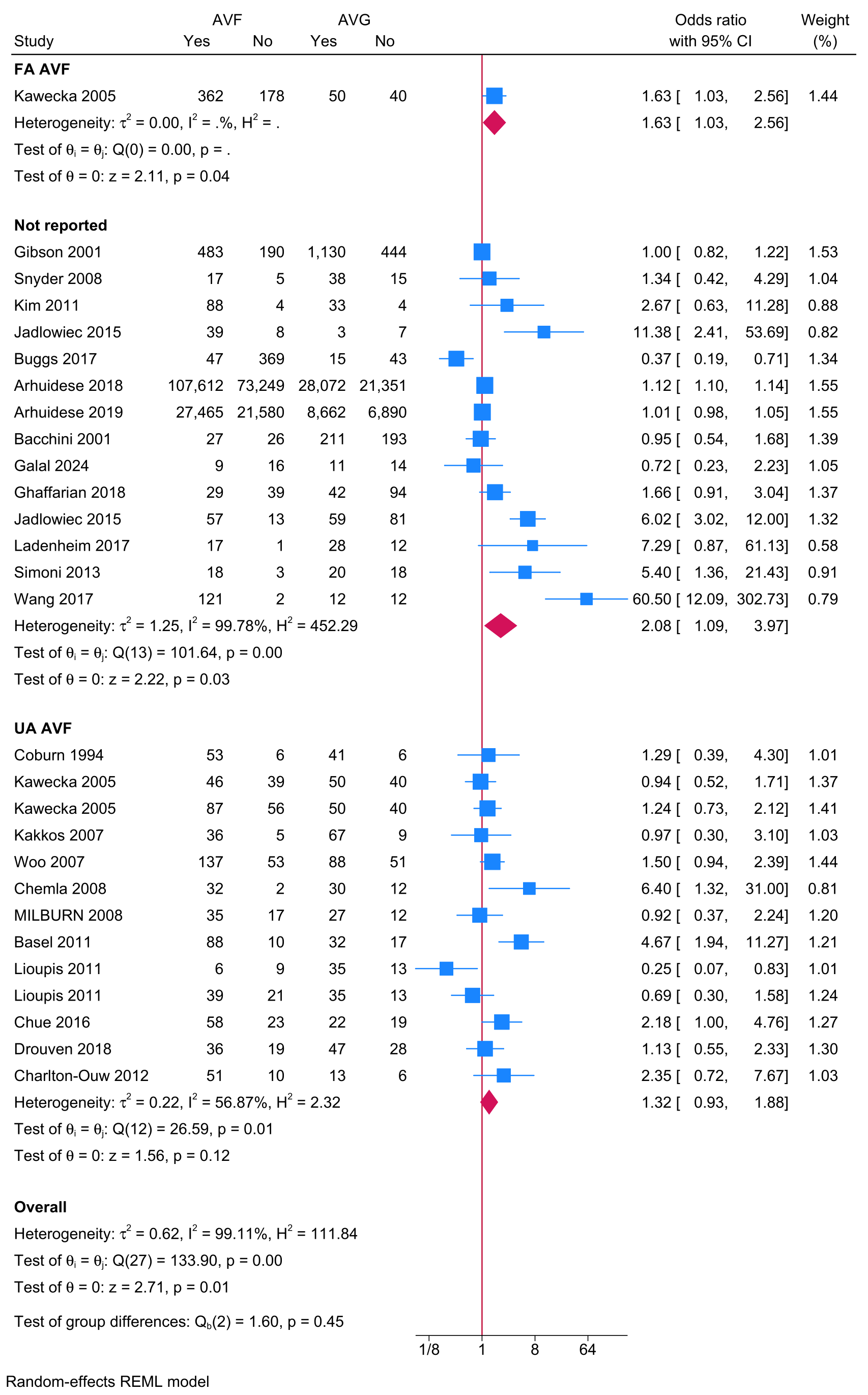
**

**Figure S12. Secondary patency by AVF site (1 year).**

**
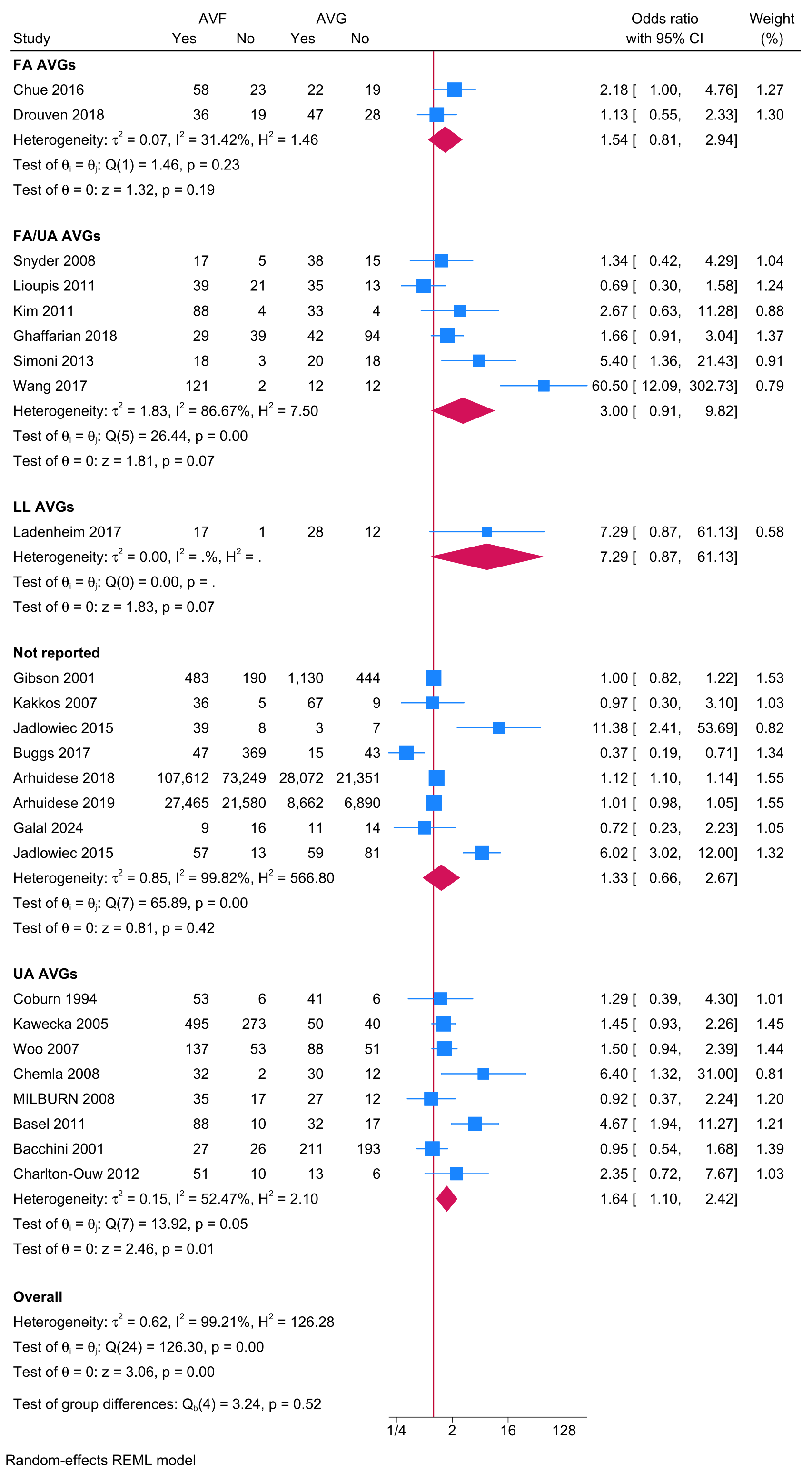
**

**Figure S13. Secondary patency by AVG site (1 year).**

**
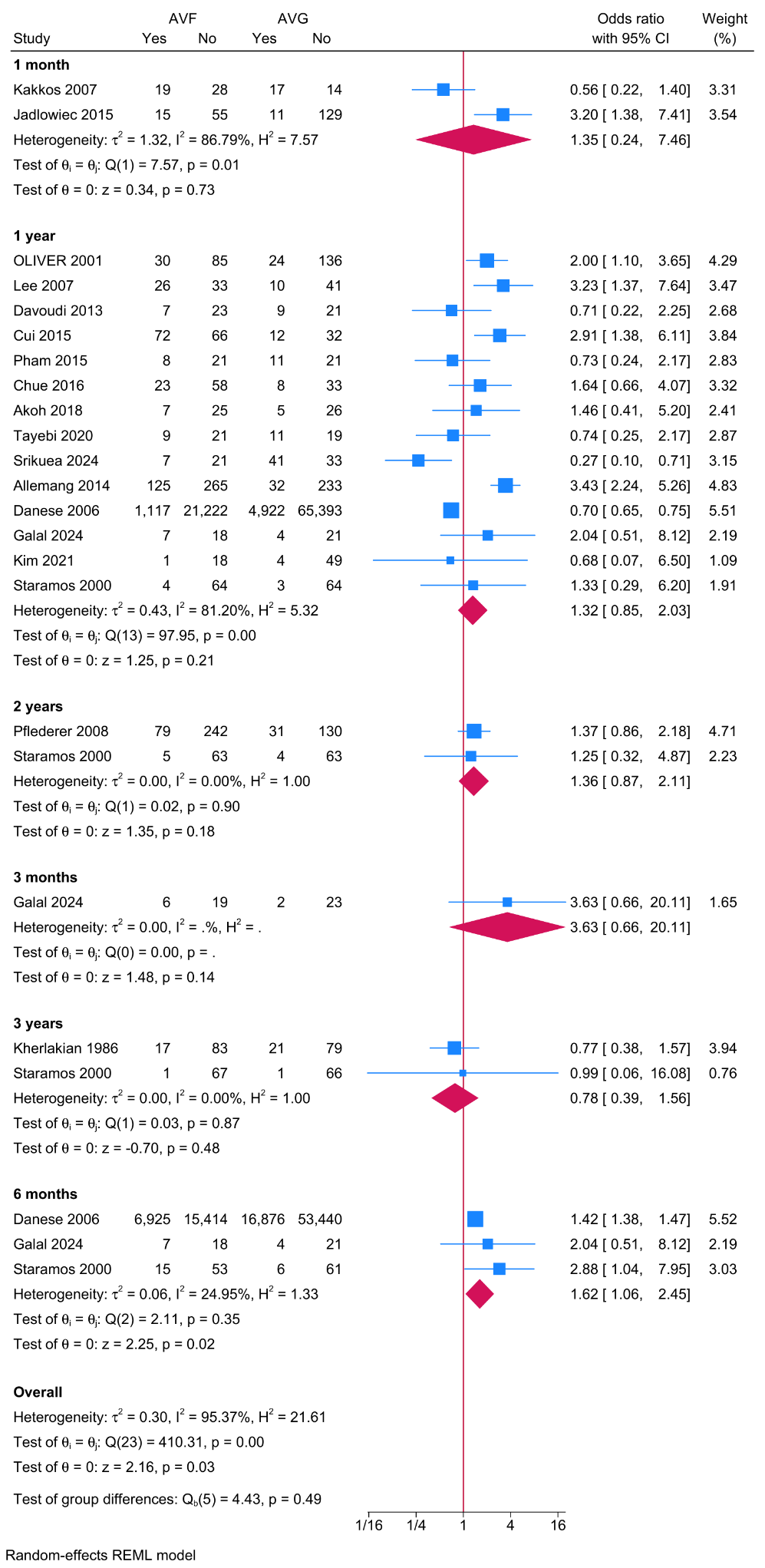
**

**Figure S14. Primary failure by time.**

**
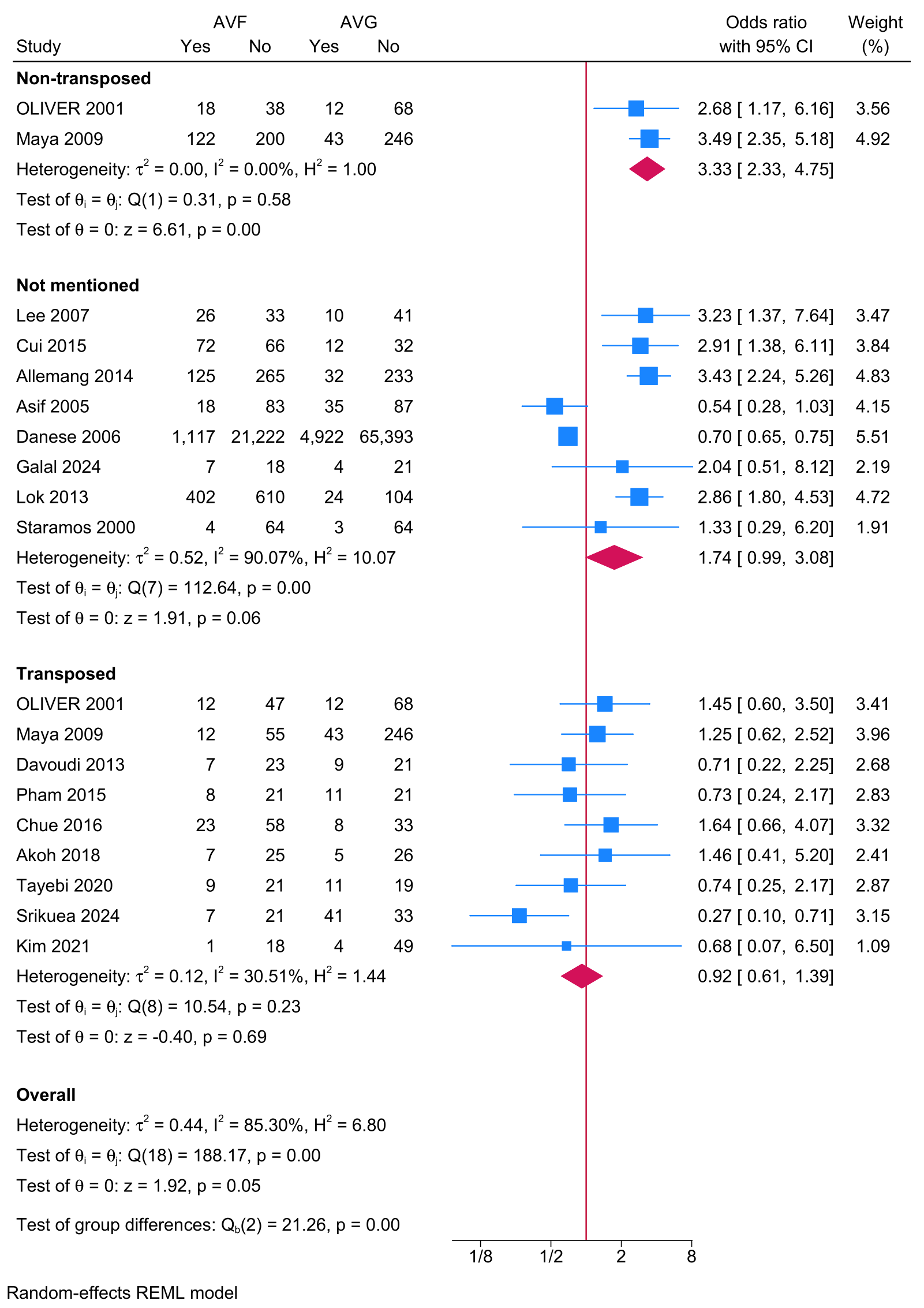
**

**Figure S15. Primary failure by AVF transposition status (1 year).**

**
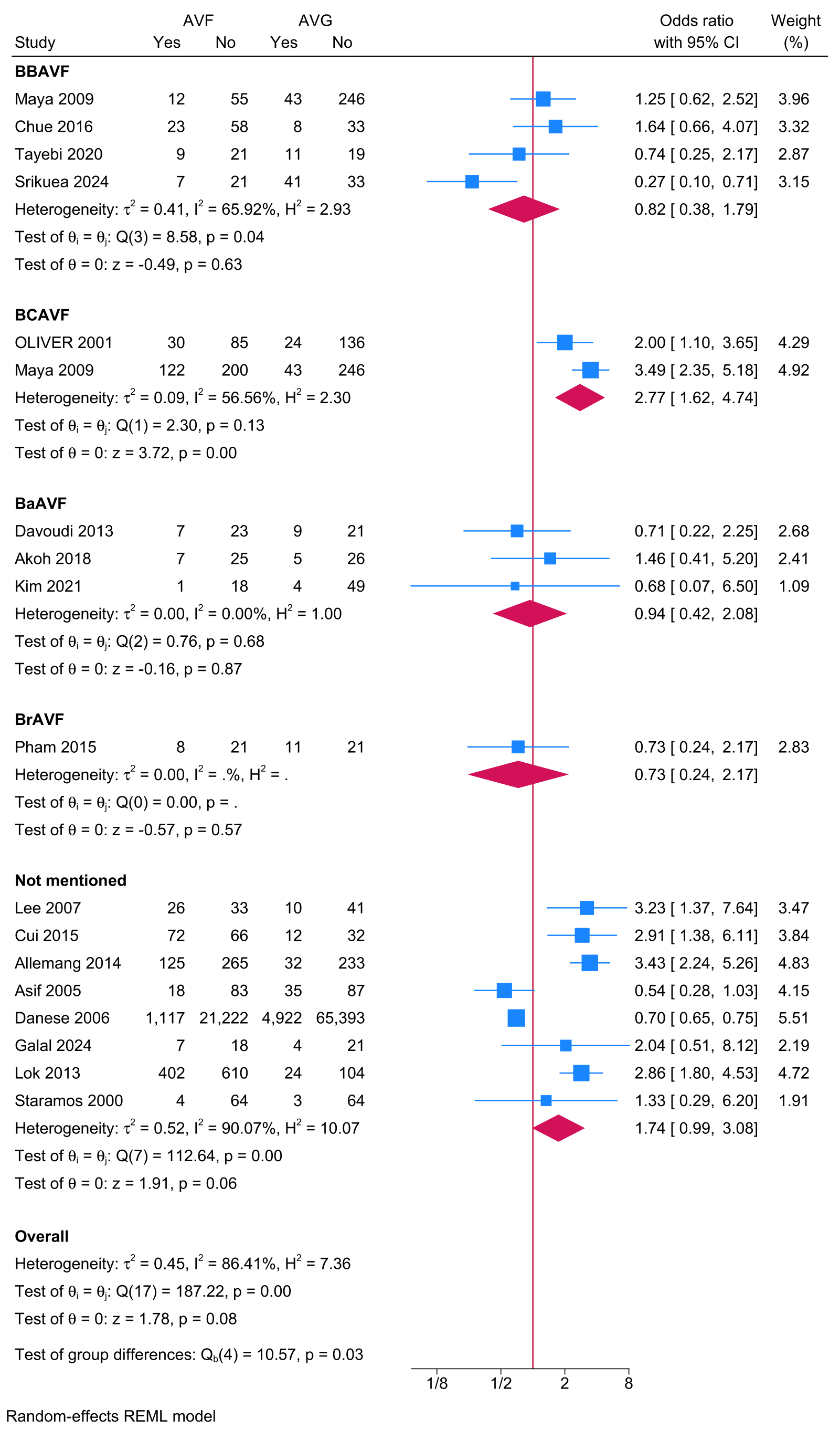
**

**Figure S16. Primary failure by AVF type (1 year).**

**
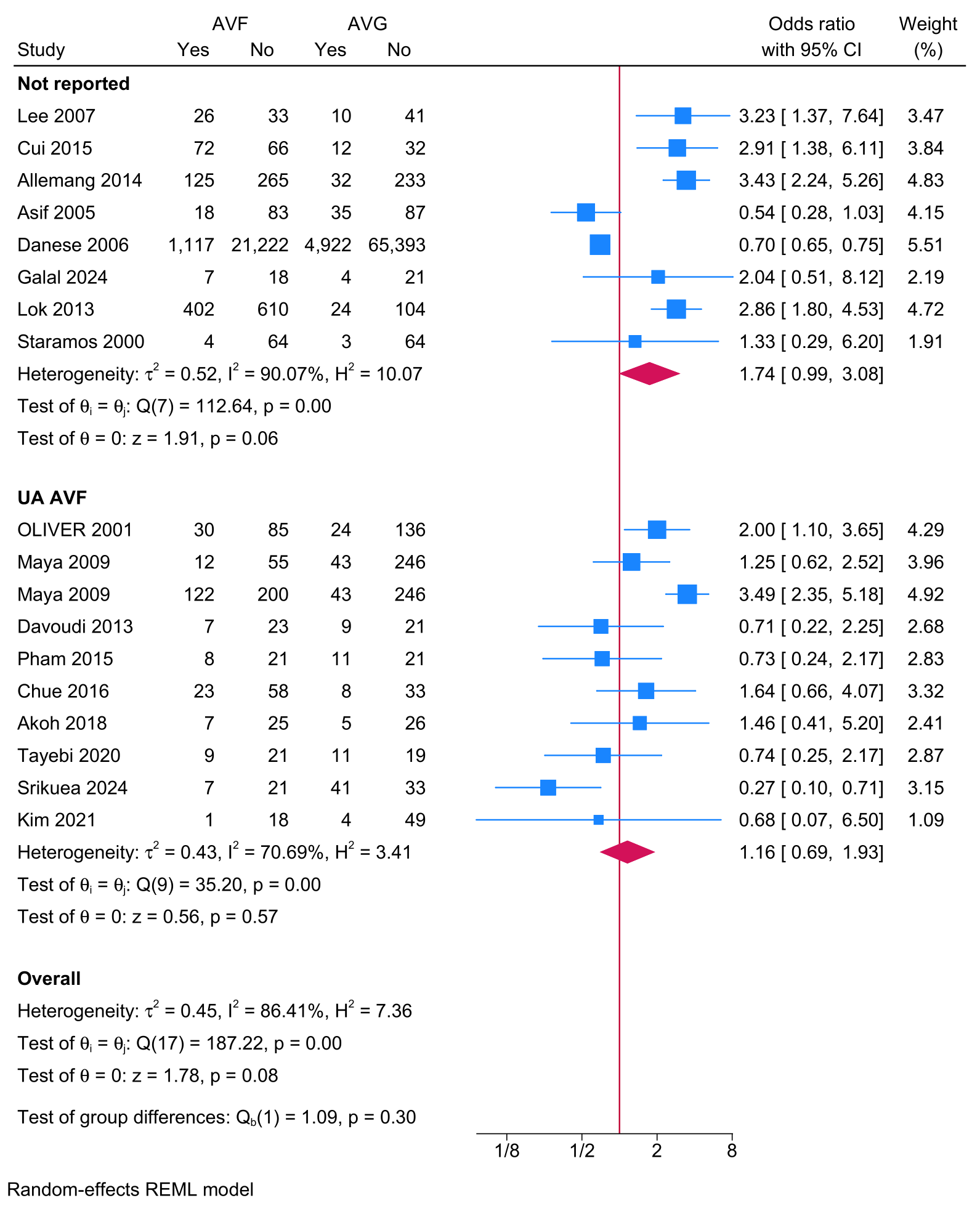
**

**Figure S17. Primary failure by AVF site (1 year).**

**
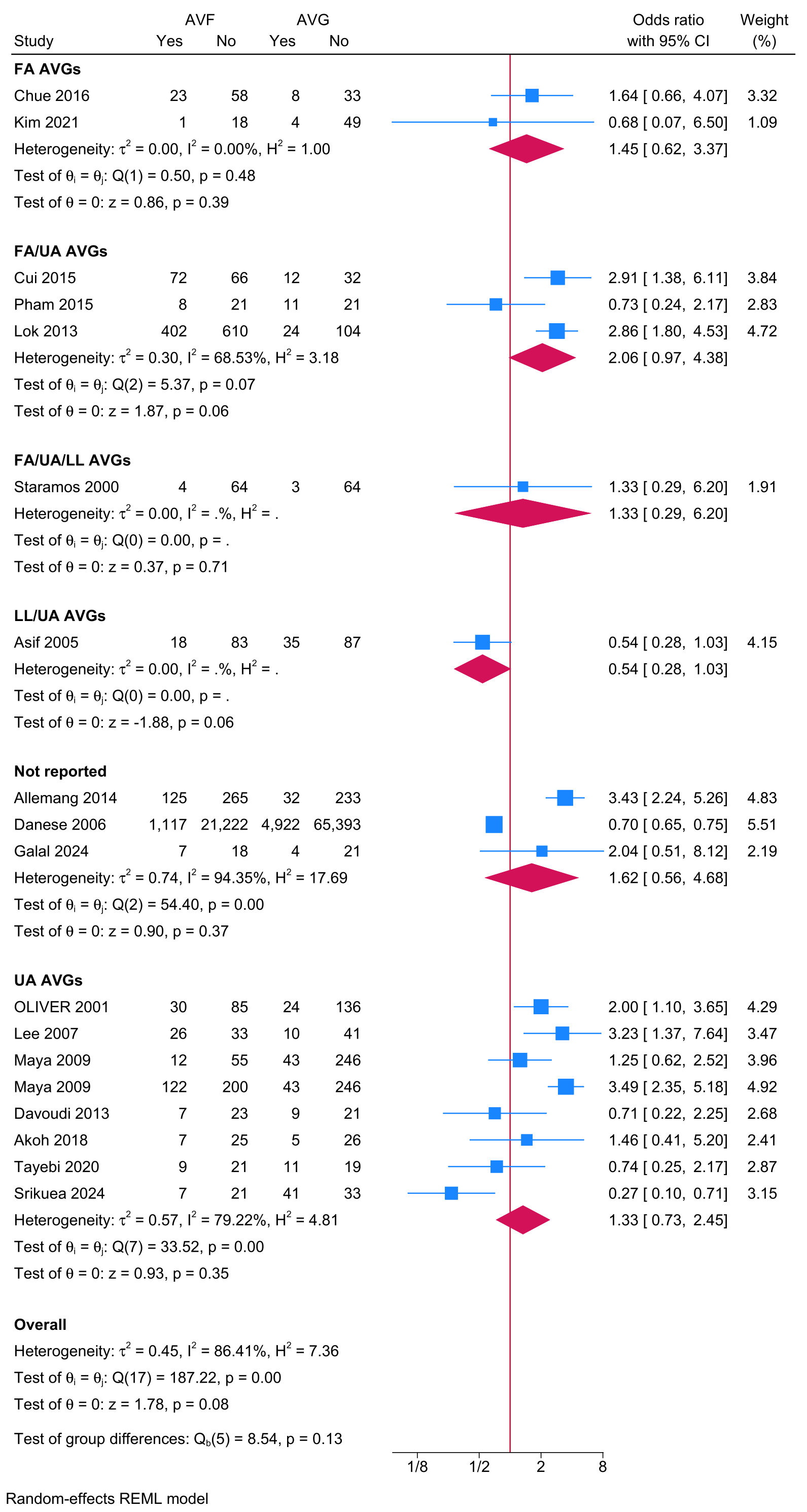
**

**Figure S18. Primary failure by AVG site (1 year).**

**
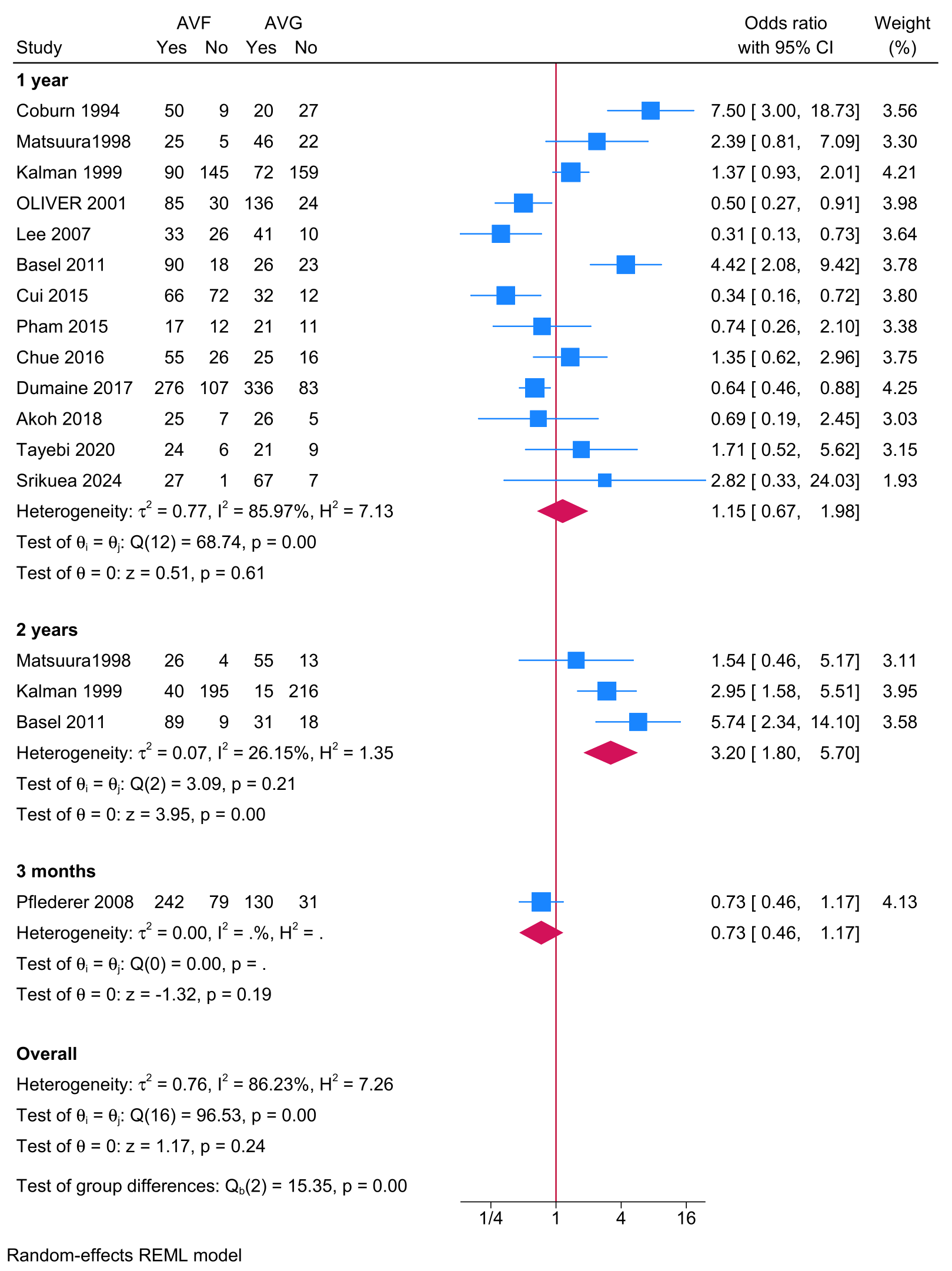
**

**Figure S19. Success by time.**

**
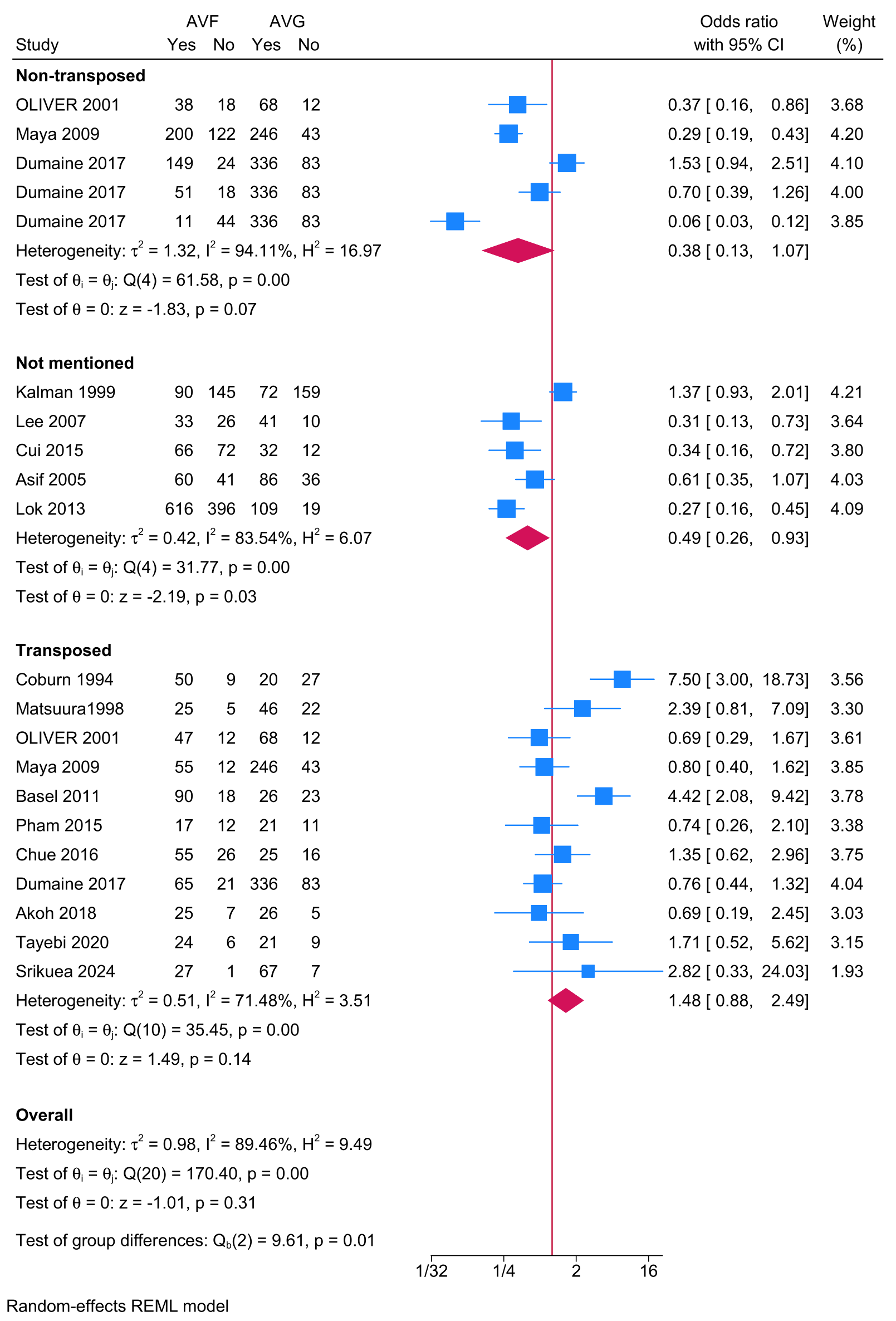
**

**Figure S20. Success by AVF transposition status (1 year).**

**
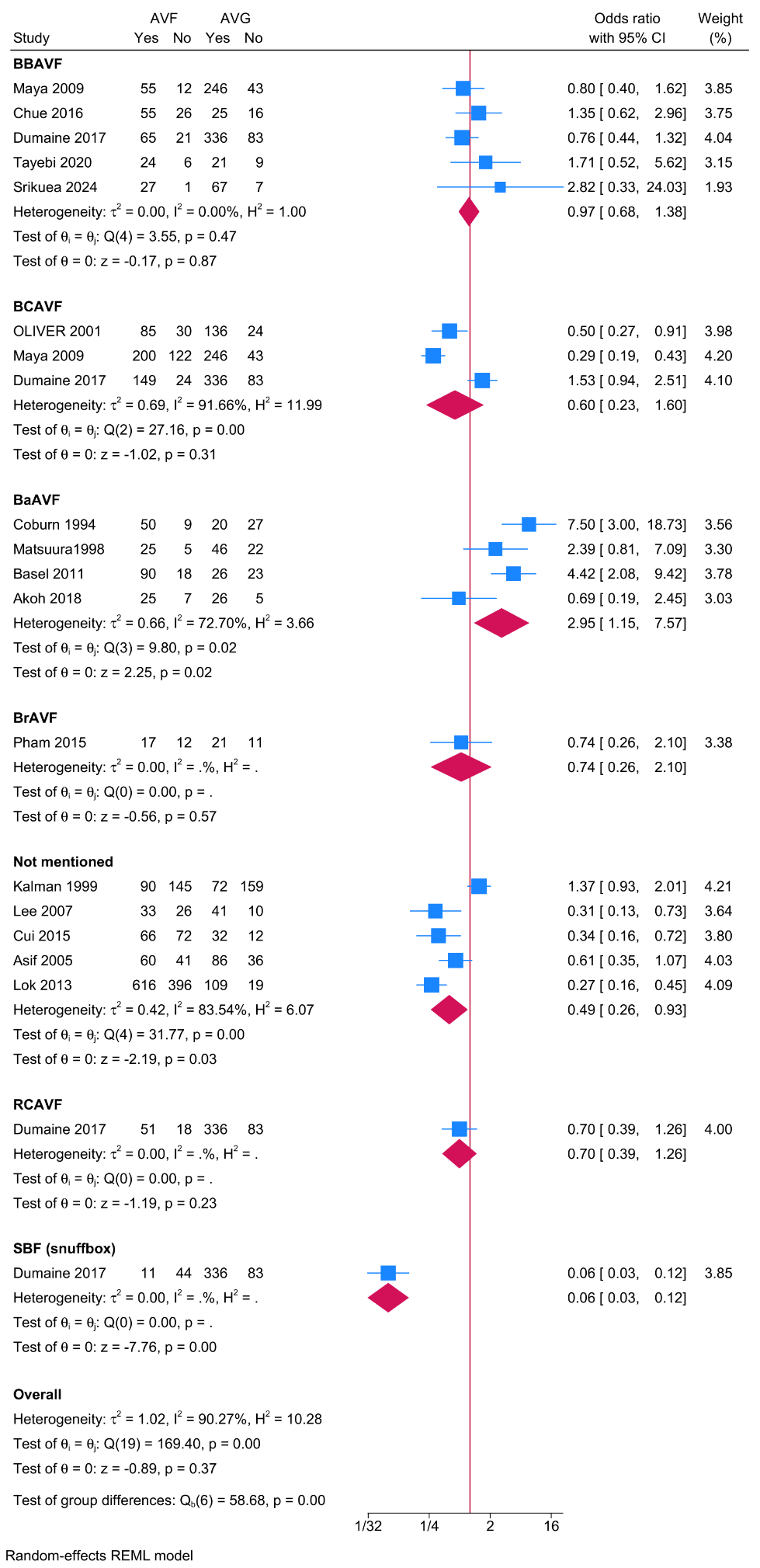
**

**Figure S21. Success by AVF type (1 year).**

**
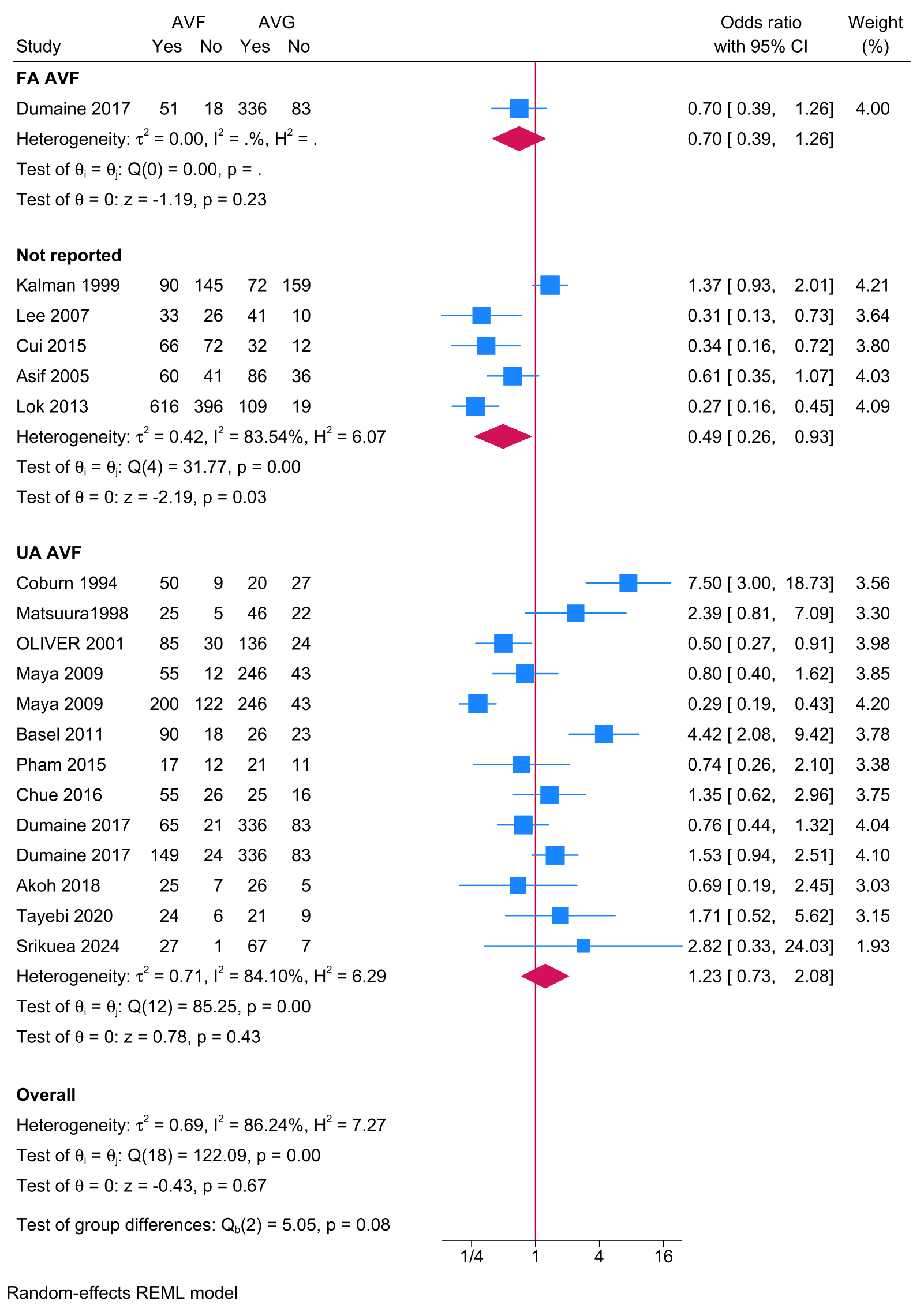
**

**Figure S22. Success by AVF site (1 year).**

**
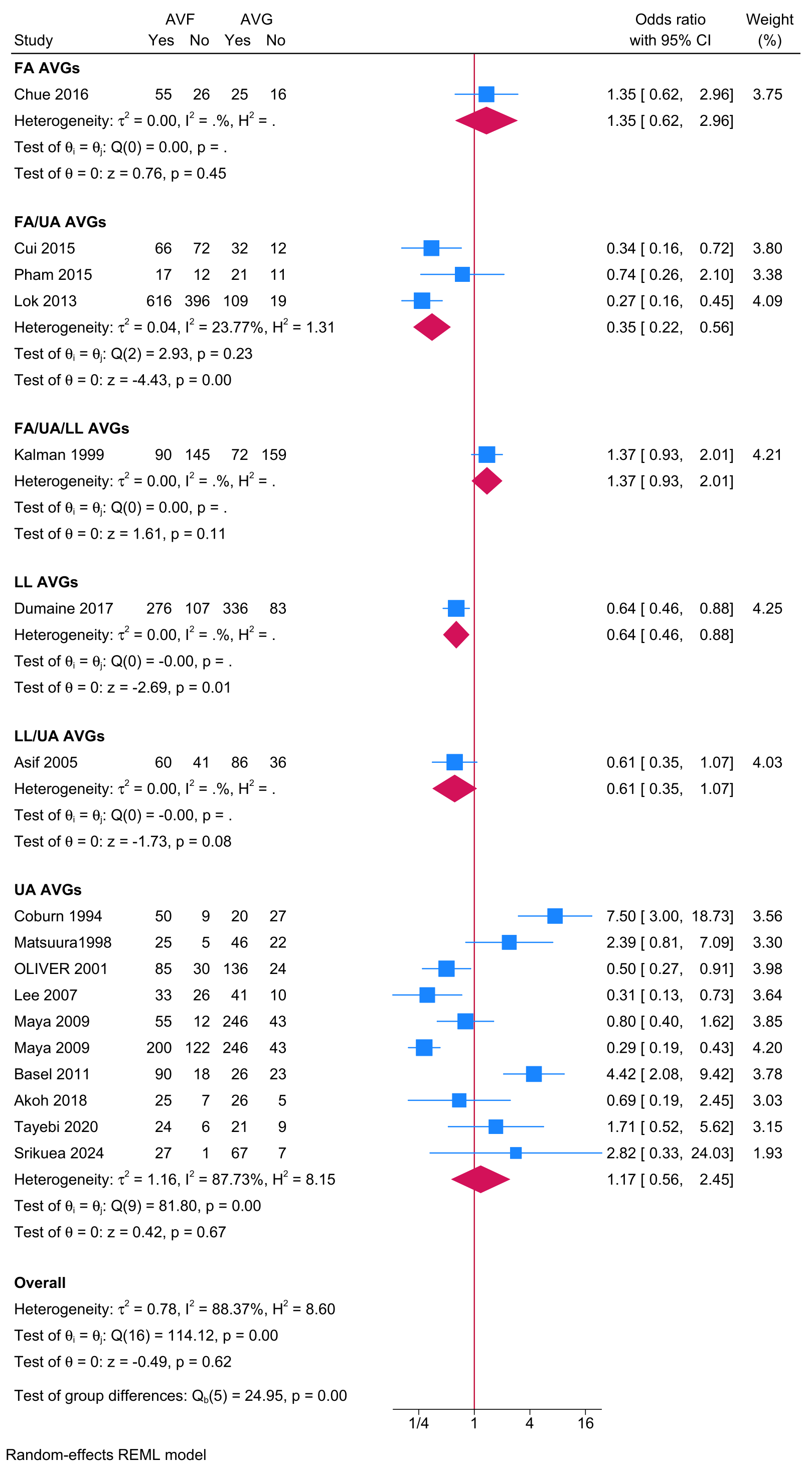
**

**Figure S23. Success by AVG site (1 year).**

**
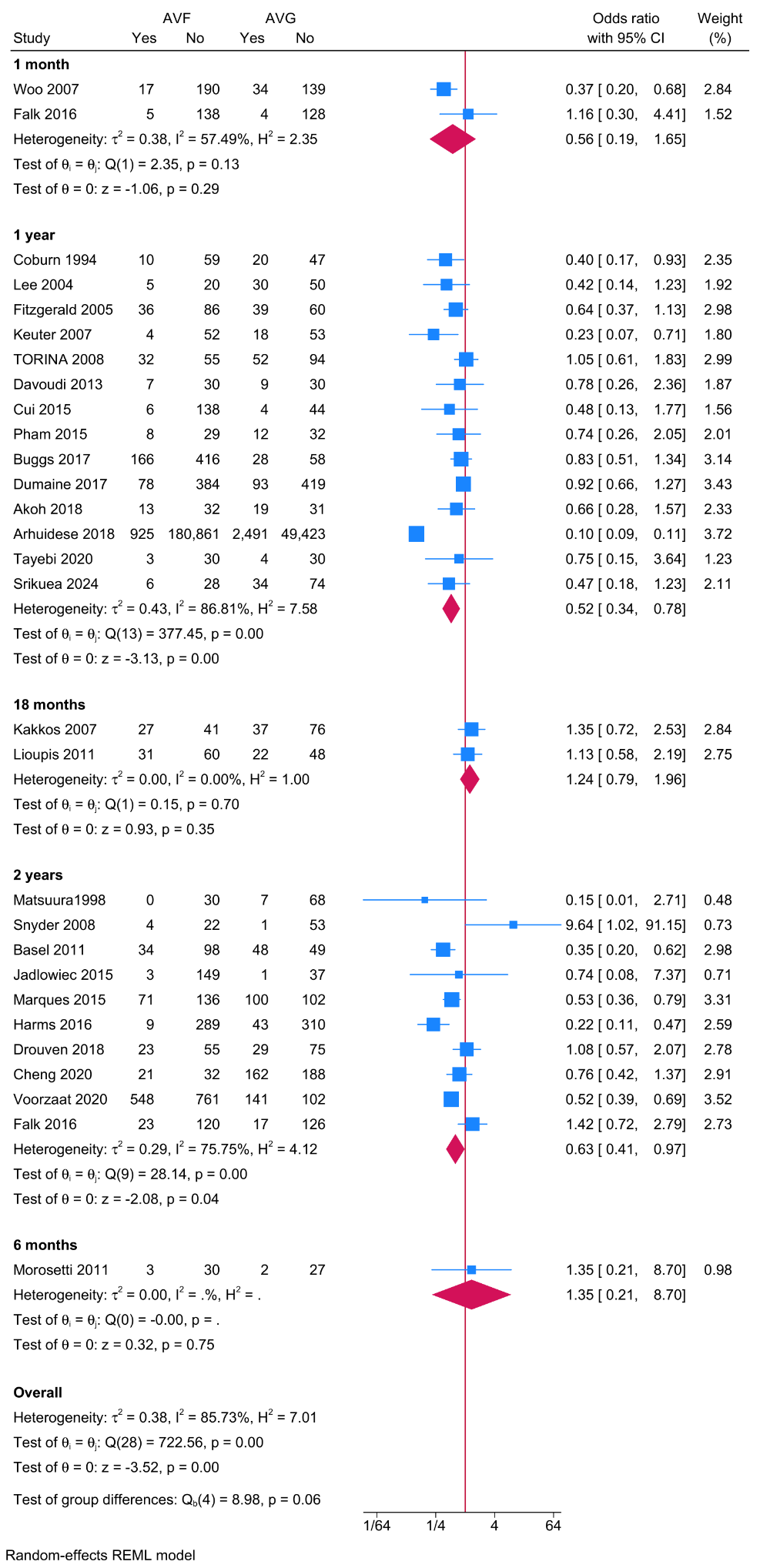
**

**Figure S24. Complications by time.**

**
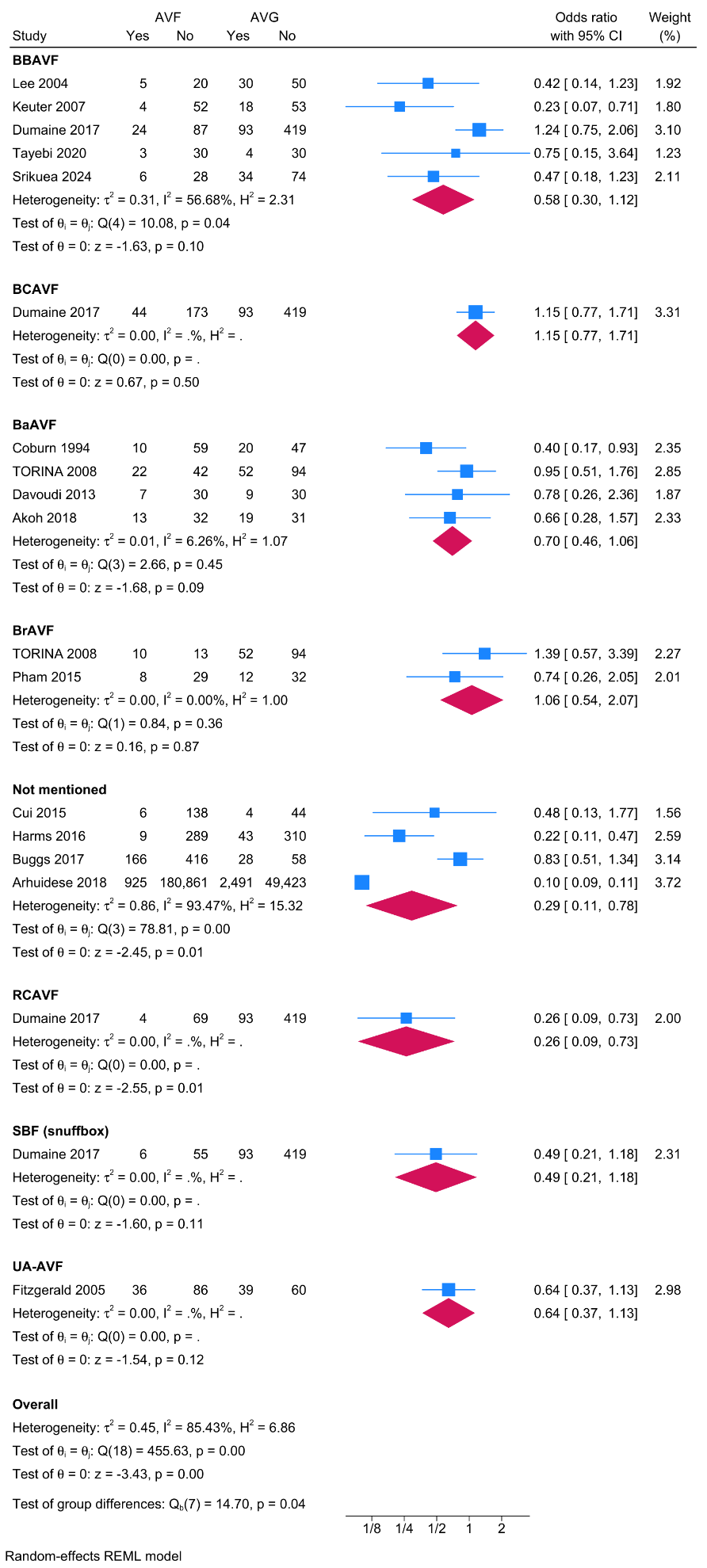
**

**Figure S25. Complications by AVF type (1 year).**

**
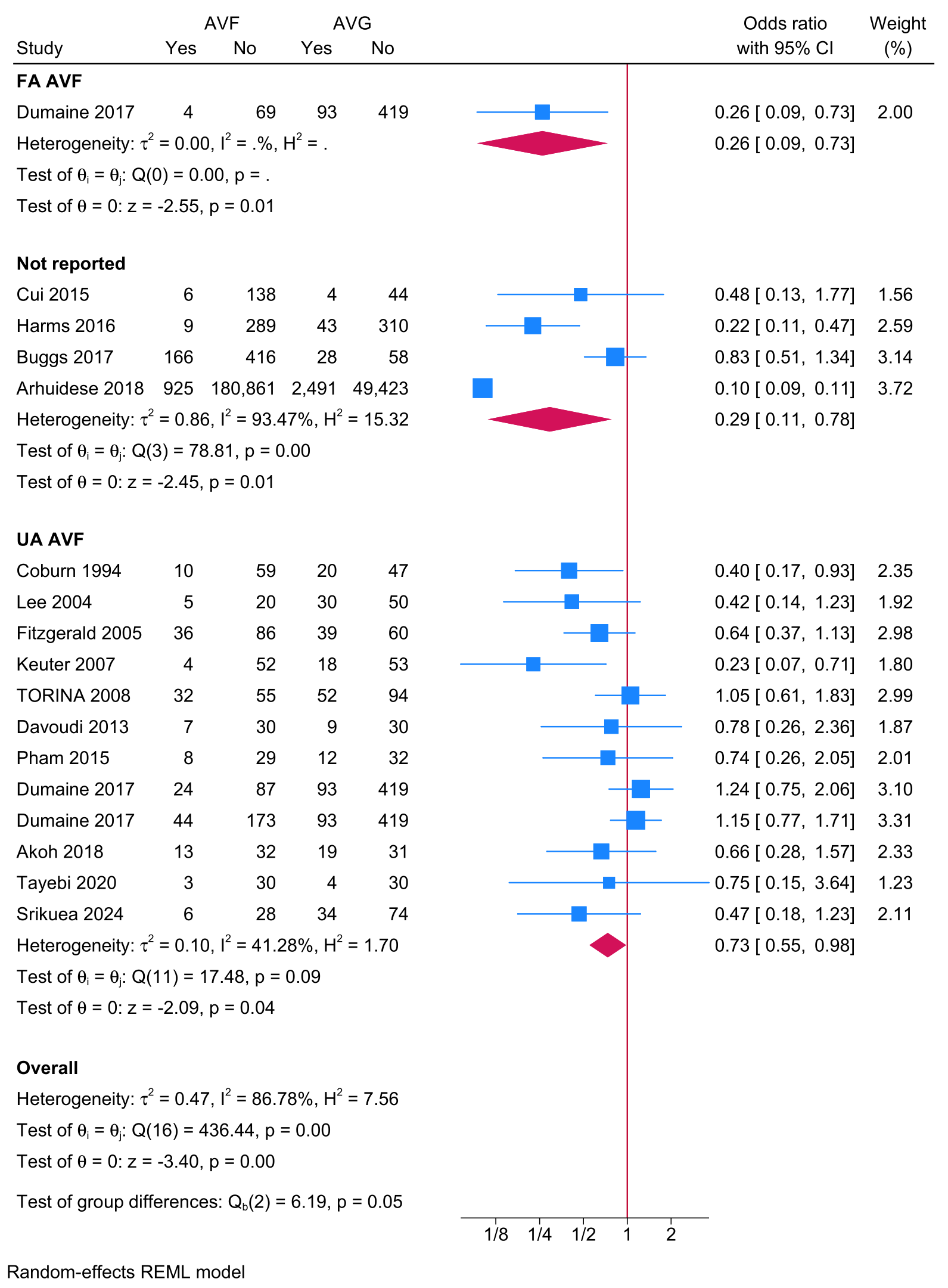
**

**Figure S26. Complications by AVF site (1 year).**

**
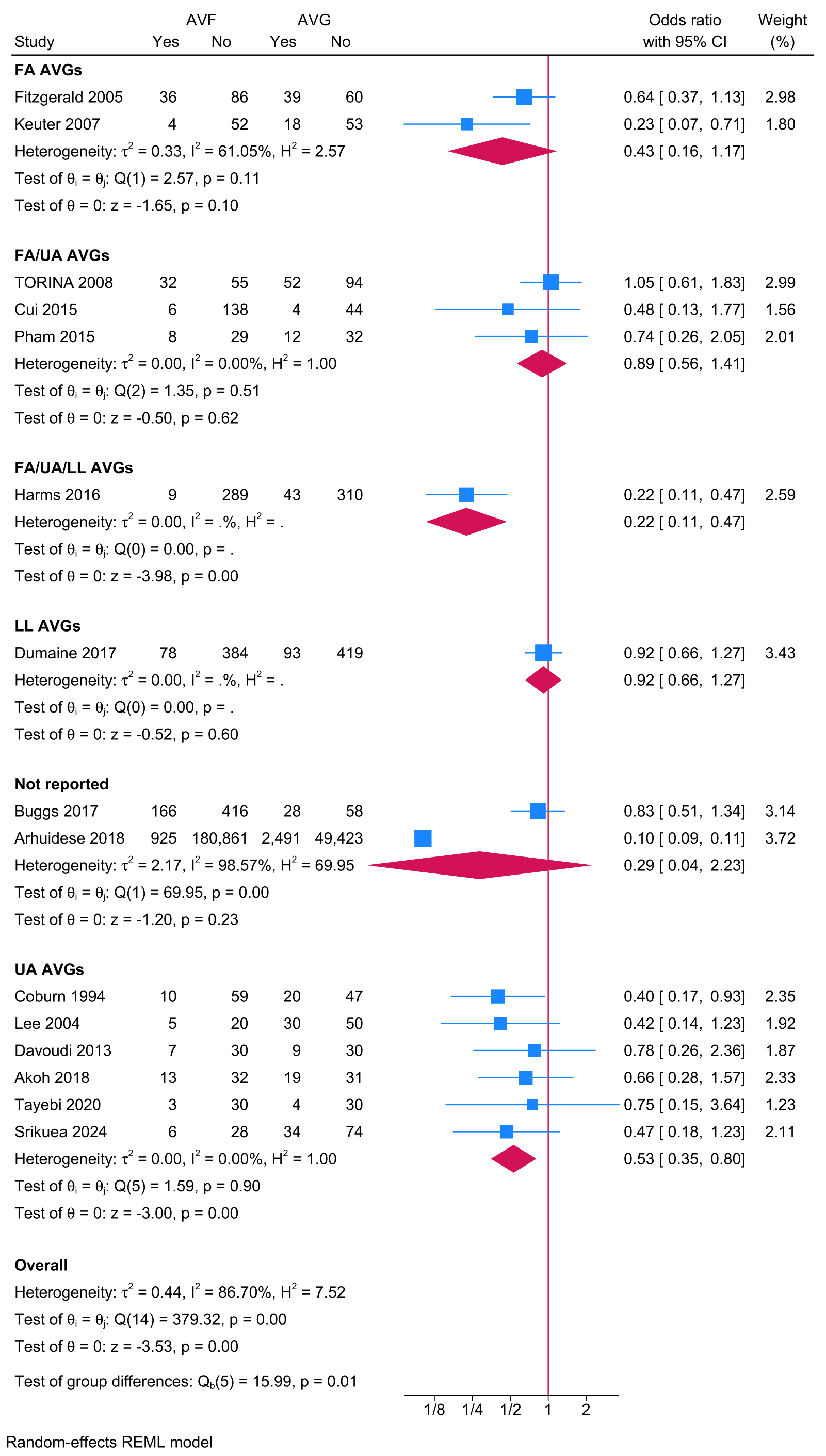
**

**Figure S27. Complications by AVG site (1 year).**

**
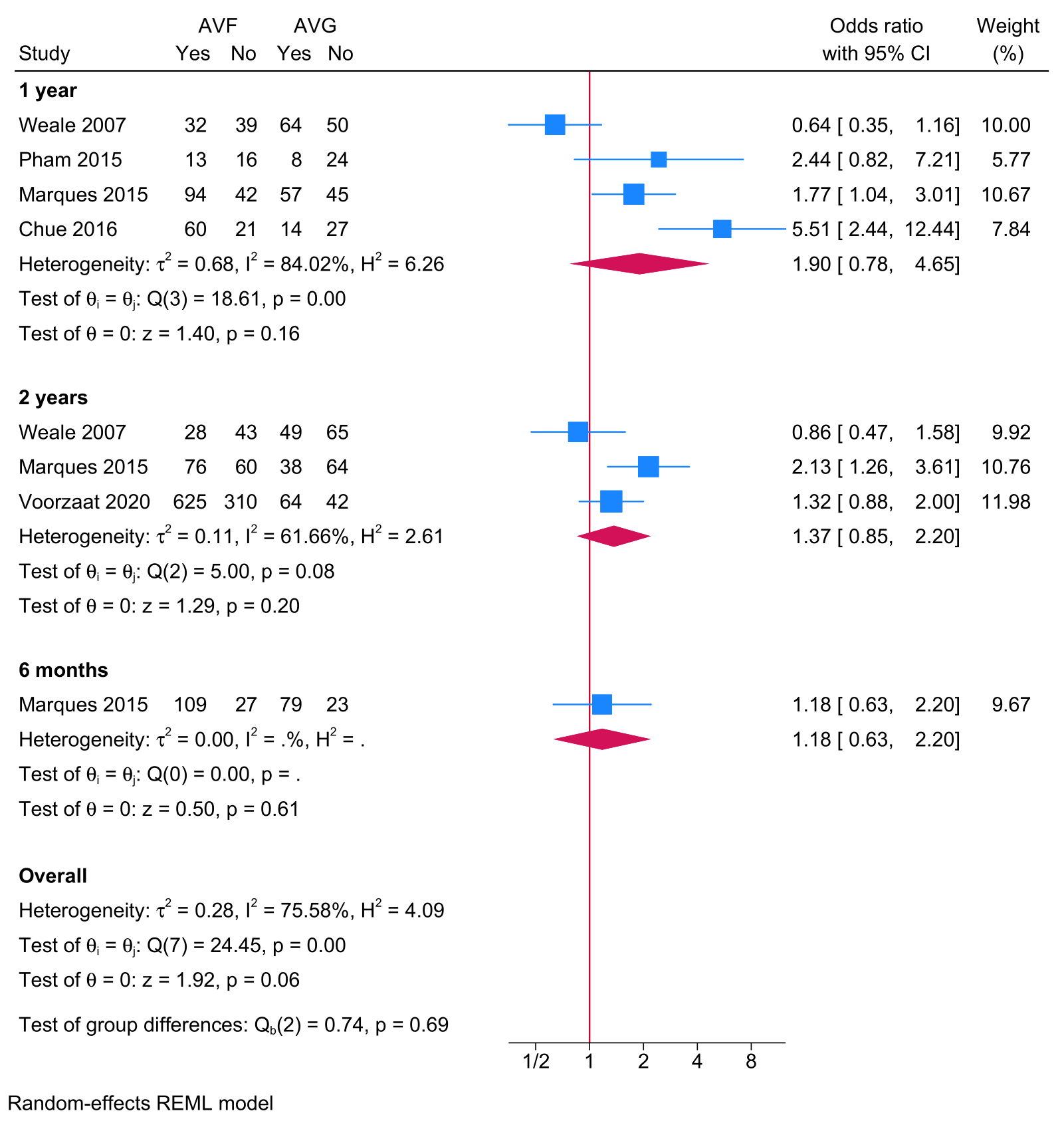
**

**Figure S28. Primary functional patency by time.**

**
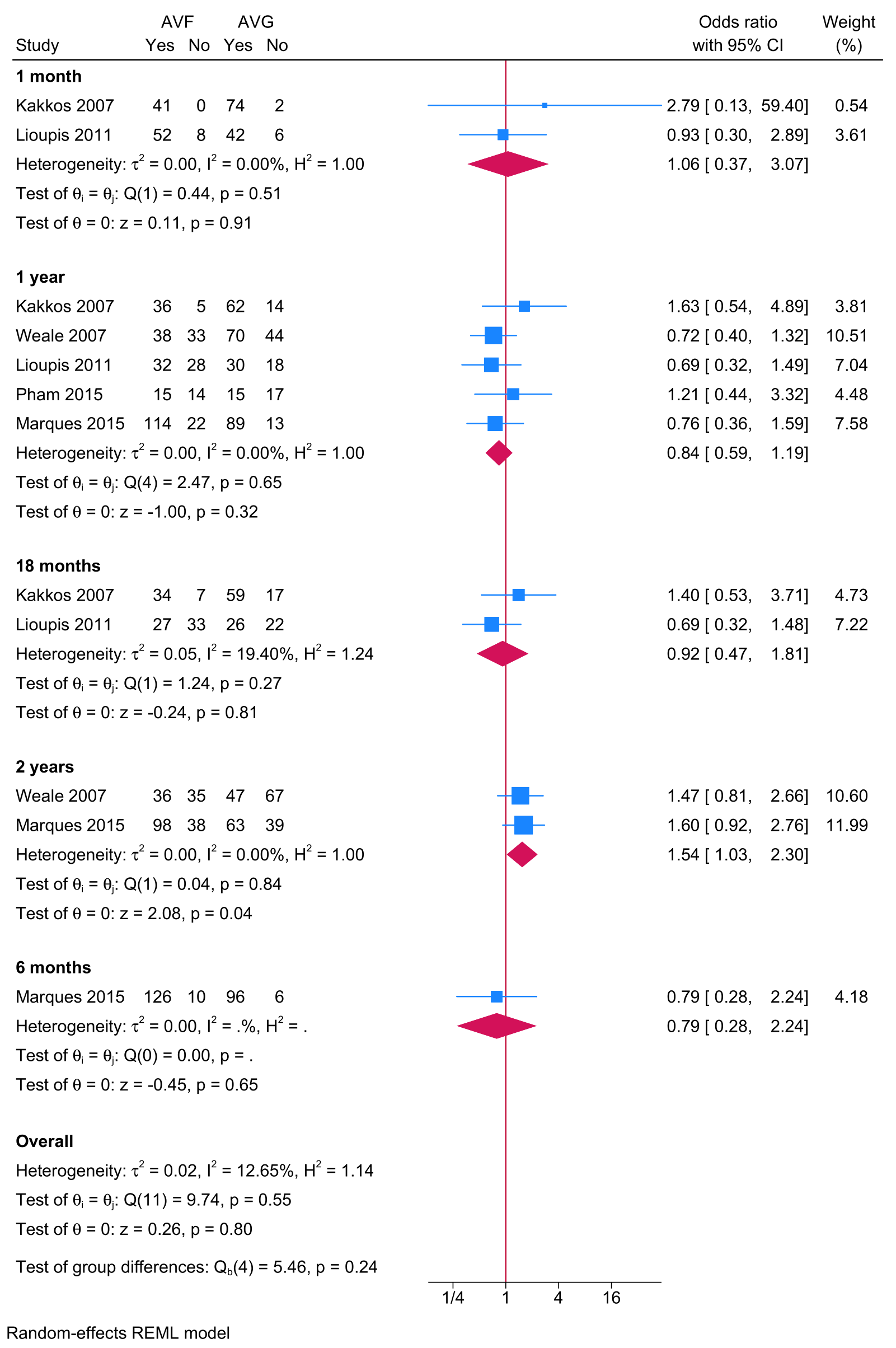
**

**Figure S29. Secondary functional patency by time.**

**
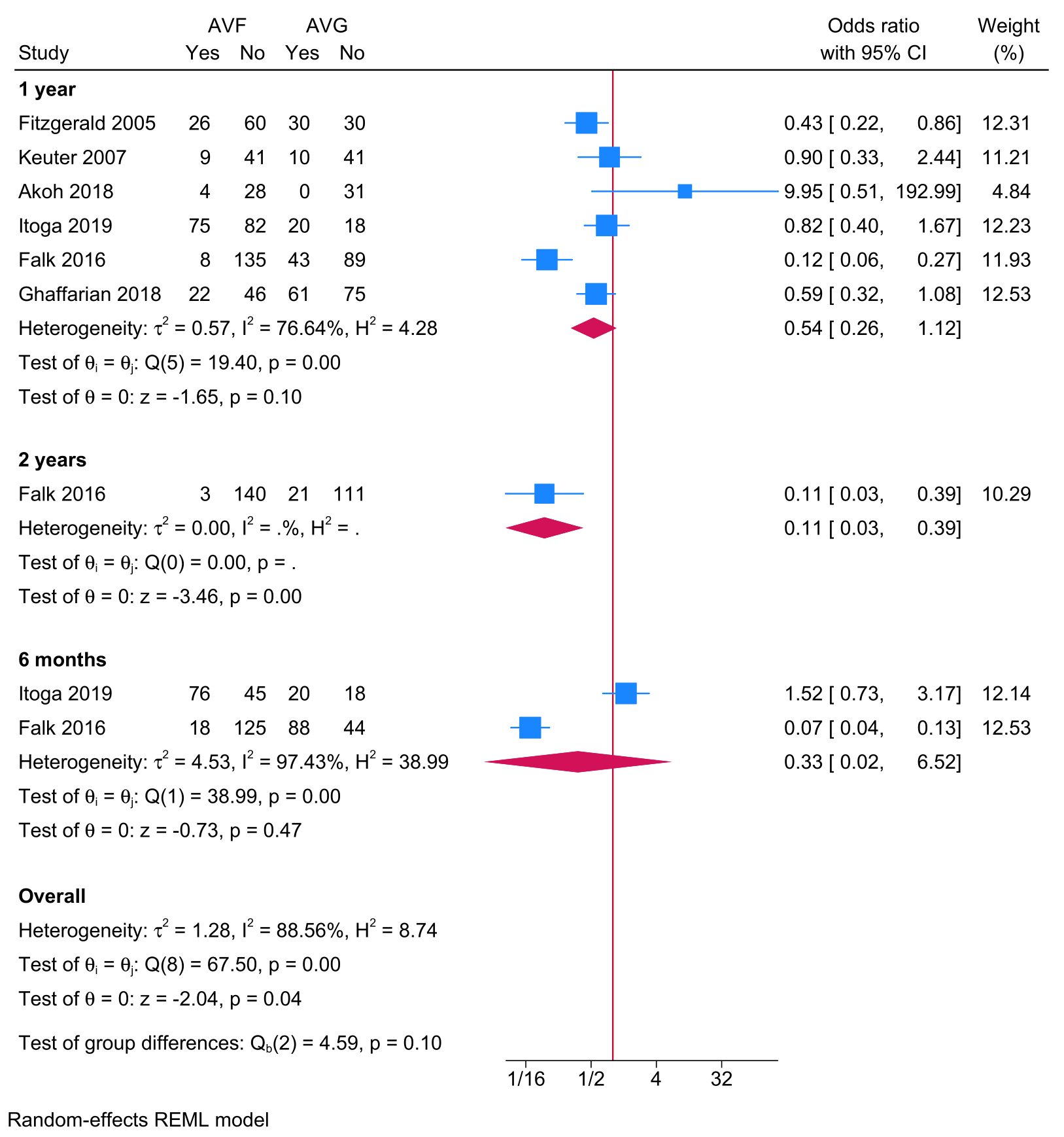
**

**Figure S30. Revision surgery by time.**

**References**

1. Akoh JA. Adoption of transposed basilic vein as access for hemodialysis. Saudi journal of kidney diseases and transplantation : an official publication of the Saudi Center for Organ Transplantation, Saudi Arabia. 2018;29(2):381-5.

2. Arhuidese IJ, Cooper MA, Rizwan M, Nejim B, Malas MB. Vascular access for hemodialysis in the elderly. Journal of vascular surgery. 2019;69(2):517-25.e1.

3. Arhuidese IJ, Orandi BJ, Nejim B, Malas M. Utilization, patency, and complications associated with vascular access for hemodialysis in the United States. Journal of vascular surgery. 2018;68(4):1166-74.

4. Ascher E, Gade P, Hingorani A, Mazzariol F, Gunduz Y, Fodera M, et al. Changes in the practice of angioaccess surgery: impact of dialysis outcome and quality initiative recommendations. Journal of vascular surgery. 2000;31(1 Pt 1):84-92.

5. Basel H, Ekim H, Odabasi D, Kiymaz A, Aydin C, Dostbil A. Basilic vein transposition fistulas versus prosthetic bridge grafts in patients with end-stage renal failure. Annals of vascular surgery. 2011;25(5):634-9.

6. Buggs J, Tanious A, Camba V, Albertson C, Rogers E, Lahiff D, et al. Effective arteriovenous fistula alternative for hemodialysis access. American journal of surgery. 2018;216(6):1144-7.

7. Chemla ES, Morsy MA. Is basilic vein transposition a real alternative to an arteriovenous bypass graft? A prospective study. Seminars in dialysis. 2008;21(4):352-6.

8. Cheng CT, Chang YC, Tam KW, Yen YC, Ko YC. Comparison Between Transposed Brachiobasilic Fistula and Arteriovenous Graft for Upper Limb Arteriovenous Access in Patients on Hemodialysis. Vascular and endovascular surgery. 2021;55(2):164-70.

9. Chue KM, Thant KZ, Luo HD, Soh YH, Ho P. Comprehensive Comparison of the Performance of Autogenous Brachial-Basilic Transposition Arteriovenous Fistula and Prosthetic Forearm Loop Arteriovenous Graft in a Multiethnic Asian Hemodialysis Population. BioMed research international. 2016;2016:8693278.

10. Coburn MC, Carney WI, Jr. Comparison of basilic vein and polytetrafluoroethylene for brachial arteriovenous fistula. Journal of vascular surgery. 1994;20(6):896-902; discussion 3-4.

11. Cui J, Steele D, Wenger J, Kawai T, Liu F, Elias N, et al. Hemodialysis arteriovenous fistula as first option not necessary in elderly patients. Journal of vascular surgery. 2016;63(5):1326-32.

12. Davoudi M, Tayebi P, Beheshtian A. Primary patency time of basilic vein transposition versus prosthetic brachioaxillary access grafts in hemodialysis patients. The journal of vascular access. 2013;14(2):111-5.

13. Dhingra RK, Young EW, Hulbert-Shearon TE, Leavey SF, Port FK. Type of vascular access and mortality in U.S. hemodialysis patients. Kidney international. 2001;60(4):1443-51.

14. Drouven JW, de Bruin C, van Roon AM, Oldenziel J, Zeebregts CJ. Outcomes of basilic vein transposition versus polytetrafluoroethylene forearm loop graft as tertiary vascular access. Journal of vascular surgery. 2019;69(4):1180-6.

15. Dumaine C, Espino-Hernandez G, Romann A, Luscombe R, Kiaii M. Femoral Arteriovenous Grafts for Hemodialysis: Retrospective Comparison With Upper Extremity Grafts and Fistulas. Canadian journal of kidney health and disease. 2017;4:2054358117719747.

16. Fitzgerald JT, Schanzer A, McVicar JP, Chin AI, Perez RV, Troppmann C. Upper arm arteriovenous fistula versus forearm looped arteriovenous graft for hemodialysis access: a comparative analysis. Annals of vascular surgery. 2005;19(6):843-50.

17. Gibson KD, Gillen DL, Caps MT, Kohler TR, Sherrard DJ, Stehman-Breen CO. Vascular access survival and incidence of revisions: a comparison of prosthetic grafts, simple autogenous fistulas, and venous transposition fistulas from the United States Renal Data System Dialysis Morbidity and Mortality Study. Journal of vascular surgery. 2001;34(4):694-700.

18. Harms JC, Rangarajan S, Young CJ, Barker-Finkel J, Allon M. Outcomes of arteriovenous fistulas and grafts with or without intervention before successful use. Journal of vascular surgery. 2016;64(1):155-62.

19. Hicks CW, Wang P, Kernodle A, Lum YW, Black JH, 3rd, Makary MA. Assessment of Use of Arteriovenous Graft vs Arteriovenous Fistula for First-time Permanent Hemodialysis Access. JAMA surgery. 2019;154(9):844-51.

20. Itoga NK, Virgin-Downy W, Mell MW. Forearm loop arteriovenous grafts preserve and may create new upper arm access sites. The journal of vascular access. 2019;20(6):691-6.

21. Jadlowiec CC, Mannion EM, Lavallee M, Brown MG. Hemodialysis Access in the Elderly: Outcomes among Patients Older than Seventy. Annals of vascular surgery. 2016;31:77-84.

22. Kakkos SK, Andrzejewski T, Haddad JA, Haddad GK, Reddy DJ, Nypaver TJ, et al. Equivalent secondary patency rates of upper extremity Vectra Vascular Access Grafts and transposed brachial-basilic fistulas with aggressive access surveillance and endovascular treatment. Journal of vascular surgery. 2008;47(2):407-14.

23. Kalman PG, Pope M, Bhola C, Richardson R, Sniderman KW. A practical approach to vascular access for hemodialysis and predictors of success. Journal of vascular surgery. 1999;30(4):727-33.

24. Kawecka A, Debska-Slizień A, Prajs J, Król E, Zdrojewski Z, Przekwas M, et al. Remarks on surgical strategy in creating vascular access for hemodialysis: 18 years of one center's experience. Annals of vascular surgery. 2005;19(4):590-8.

25. Keuter XH, De Smet AA, Kessels AG, van der Sande FM, Welten RJ, Tordoir JH. A randomized multicenter study of the outcome of brachial-basilic arteriovenous fistula and prosthetic brachial-antecubital forearm loop as vascular access for hemodialysis. Journal of vascular surgery. 2008;47(2):395-401.

26. Kim DS, Kim SW, Kim JC, Cho JH, Kong JH, Park CR. Clinical analysis of hemodialysis vascular access: comparision of autogenous arterioveonus fistula & arteriovenous prosthetic graft. The Korean journal of thoracic and cardiovascular surgery. 2011;44(1):25-31.

27. Lee CH, Ko PJ, Liu YH, Hsieh HC, Liu HP. Brachiobasilic fistula as a secondary access procedure: an alternative to a dialysis prosthetic graft. Chang Gung medical journal. 2004;27(11):816-23.

28. Lee T, Barker J, Allon M. Comparison of survival of upper arm arteriovenous fistulas and grafts after failed forearm fistula. Journal of the American Society of Nephrology : JASN. 2007;18(6):1936-41.

29. Lioupis C, Mistry H, Rix T, Chandak P, Tyrrell M, Valenti D. Comparison among transposed brachiobasilic, brachiobrachial arteriovenous fistulas and Flixene™ vascular graft. The journal of vascular access. 2011;12(1):36-44.

30. Marques G, Sadaghianloo N, Fouilhé L, Jean-Baptiste E, Declemy S, Clément C, et al. Higher patency of transposed brachio-basilic arteriovenous fistulas compared to brachio-axillary grafts for hemodialysis patients. The journal of vascular access. 2015;16(6):486-92.

31. Matsuura JH, Rosenthal D, Clark M, Shuler FW, Kirby L, Shotwell M, et al. Transposed basilic vein versus polytetrafluorethylene for brachial-axillary arteriovenous fistulas. American journal of surgery. 1998;176(2):219-21.

32. Maya ID, O'Neal JC, Young CJ, Barker-Finkel J, Allon M. Outcomes of brachiocephalic fistulas, transposed brachiobasilic fistulas, and upper arm grafts. Clinical journal of the American Society of Nephrology : CJASN. 2009;4(1):86-92.

33. Milburn JA, Lo ST, Szucs ZJ, Humphrey A, Macaulay EM. Transposed brachiobasilic fistula or PTFE arm graft - alternative or complementary? The journal of vascular access. 2008;9(2):117-21.

34. Morosetti M, Cipriani S, Dominijanni S, Pisani G, Frattarelli D, Bruno F. Basilic vein transposition versus biosynthetic prosthesis as vascular access for hemodialysis. Journal of vascular surgery. 2011;54(6):1713-9.

35. Oliver MJ, McCann RL, Indridason OS, Butterly DW, Schwab SJ. Comparison of transposed brachiobasilic fistulas to upper arm grafts and brachiocephalic fistulas. Kidney international. 2001;60(4):1532-9.

36. Park HS, Kim WJ, Kim YK, Kim HW, Choi BS, Park CW, et al. Comparison of Outcomes with Arteriovenous Fistula and Arteriovenous Graft for Vascular Access in Hemodialysis: A Prospective Cohort Study. American journal of nephrology. 2016;43(2):120-8.

37. Pflederer TA, Kwok S, Ketel BL, Pilgram T. A comparison of transposed brachiobasilic fistulae with nontransposed fistulae and grafts in the Fistula First era. Seminars in dialysis. 2008;21(4):357-63.

38. Pham XD, Kim JJ, Ihenachor EJ, Parrish AB, Bleck JD, Kaji AH, et al. A comparison of brachial artery-brachial vein arteriovenous fistulas with arteriovenous grafts in patients with poor superficial venous anatomy. Journal of vascular surgery. 2017;65(2):444-51.

39. Snyder DC, Clericuzio CP, Stringer A, May W. Comparison of outcomes of arteriovenous grafts and fistulas at a single Veterans' Affairs medical center. American journal of surgery. 2008;196(5):641-6.

40. Srikuea K, Prajumsukh K, Orrapin S, Benyakorn T, Ho P, Rerkasem K, et al. One-staged brachial-basilic vein transposition versus arm straight arteriovenous graft for hemodialysis. Vascular. 2024:17085381241245068.

41. Tayebi P, Kazemzadeh G, Modaghegh MHS, Kamyar MM, Ravari H. Brachio-basilic upper arm transposition fistulas vs. prosthetic brachio-axillary vascular access grafts-Which one is preferred for hemodialysis? Hemodialysis international International Symposium on Home Hemodialysis. 2020;24(2):182-7.

42. Torina PJ, Westheimer EF, Schanzer HR. Brachial vein transposition arteriovenous fistula: is it an acceptable option for chronic dialysis vascular access? The journal of vascular access. 2008;9(1):39-44.

43. Voorzaat BM, Janmaat CJ, van der Bogt KEA, Dekker FW, Rotmans JI. Patency Outcomes of Arteriovenous Fistulas and Grafts for Hemodialysis Access: A Trade-Off between Nonmaturation and Long-Term Complications. Kidney360. 2020;1(9):916-24.

44. Weale AR, Bevis P, Neary WD, Lear PA, Mitchell DC. A comparison between transposed brachiobasilic arteriovenous fistulas and prosthetic brachioaxillary access grafts for vascular access for hemodialysis. Journal of vascular surgery. 2007;46(5):997-1004.

45. Woo K, Farber A, Doros G, Killeen K, Kohanzadeh S. Evaluation of the efficacy of the transposed upper arm arteriovenous fistula: a single institutional review of 190 basilic and cephalic vein transposition procedures. Journal of vascular surgery. 2007;46(1):94-9; discussion 100.

46. Yan Y, Clark TW, Mondschein JI, Shlansky-Goldberg RD, Dagli MS, Soulen MC, et al. Outcomes of percutaneous interventions in transposed hemodialysis fistulas compared with nontransposed fistulas and grafts. Journal of vascular and interventional radiology : JVIR. 2013;24(12):1765-72; quiz 73.

47. Yuo TH, Chaer RA, Dillavou ED, Leers SA, Makaroun MS. Patients started on hemodialysis with tunneled dialysis catheter have similar survival after arteriovenous fistula and arteriovenous graft creation. Journal of vascular surgery. 2015;62(6):1590-7.e2.

48. Allemang MT, Schmotzer B, Wong VL, Lakin RO, Woodside KJ, Schulak JA, et al. Arteriovenous grafts have higher secondary patency in the short term compared with autologous fistulae. The American Journal of Surgery. 2014;208(5):800-5.

49. Asif A, Gadalean FN, Merrill D, Cherla G, Cipleu CD, Epstein DL, et al. Inflow stenosis in arteriovenous fistulas and grafts: a multicenter, prospective study. Kidney international. 2005;67(5):1986-92.

50. Bacchini G, Del Vecchio L, Andrulli S, Pontoriero G, Locatelli F. Survival of prosthetic grafts of different materials after impairment of a native arteriovenous fistula in hemodialysis patients. ASAIO journal. 2001;47(1):30-3.

51. Charlton-Ouw KM, Nosrati N, Miller 3rd CC, Coogan SM, Safi HJ, Azizzadeh A. Outcomes of arteriovenous fistulae compared with heparin-bonded and conventional grafts for hemodialysis access. The journal of vascular access. 2012;13(2):163-7.

52. Danese M, Liu Z, Griffiths R, Dylan M, Yu H-T, Dubois R, et al. Catheter use is high even among hemodialysis patients with a fistula or graft. Kidney international. 2006;70(8):1482-5.

53. Falk A, Maya ID, Yevzlin AS, Investigators R. A prospective, randomized study of an expanded polytetrafluoroethylene stent graft versus balloon angioplasty for in-stent restenosis in arteriovenous grafts and fistulae: two-year results of the RESCUE study. Journal of Vascular and Interventional Radiology. 2016;27(10):1465-76.

54. Galal AM, Ismail MA, Abdrabo MS, Mahmoud AK. Saphenous vein versus synthetic graft in arteriovenous fistula for hemodialysis in patient with inaccessible veins. 2024.

55. Ghaffarian AA, Al-Dulaimi R, Kraiss LW, Sarfati M, Griffin CL, Smith BK, et al. Clinical effectiveness of open thrombectomy for thrombosed autogenous arteriovenous fistulas and grafts. Journal of vascular surgery. 2018;68(1):189-96.

56. Kherlakian GM, Roedershelmer LR, Arbaugh JJ, Newmark KJ, King LR. Comparison of autogenous fistula versus expanded polytetrafluoroethylene graft fistula for angioaccess in hemodialysis. The American journal of surgery. 1986;152(2):238-43.

57. Kim H, Ahn S, Kim M, Chung CTY, Choi Kw, Ko H, et al. Comparison between autogenous brachial–brachial upper-arm elevated direct arteriovenous fistulas and prosthetic brachial-antecubital indirect forearm arteriovenous grafts. The journal of vascular access. 2022;23(6):936-45.

58. Ladenheim ED, Lulic D, Lum C, Agrawal S. Primary and secondary patencies of transposed femoral vein fistulas are significantly greater than with the HeRO graft. The journal of vascular access. 2017;18(3):232-7.

59. Lok CE, Sontrop JM, Tomlinson G, Rajan D, Cattral M, Oreopoulos G, et al. Cumulative patency of contemporary fistulas versus grafts (2000–2010). Clinical Journal of the American Society of Nephrology. 2013;8(5):810-8.

60. Simoni E, Blitz L, Lookstein R. Outcomes of AngioJet® thrombectomy in hemodialysis vascular access grafts and fistulas: PEARL I Registry. The journal of vascular access. 2013;14(1):72-6.

61. Staramos DN, Lazarides MK, Tzilalis VD, Ekonomou CS, Simopoulos CE, Dayantas JN. Patency of autologous and prosthetic arteriovenous fistulas in elderly patients. European Journal of Surgery. 2000;166(10):777-81.

62. Wang S, Wang MS. Successful use of partial aneurysmectomy and repair approach for managing complications of arteriovenous fistulas and grafts. Journal of vascular surgery. 2017;66(2):545-53.

63. Jadlowiec CC, Lavallee M, Mannion EM, Brown MG. An outcomes comparison of native arteriovenous fistulae, polytetrafluorethylene grafts, and cryopreserved vein allografts. Annals of vascular surgery. 2015;29(8):1642-7.
